# Reactions of Platinum Terminal Polyynyl Complexes *trans*-(C_6_F_5_)(*p*-tol_3_P)_2_Pt(C≡C)_*n*_H (*n* = 2–4) and *n*-BuLi, Generation of Functional Equivalents of Pt(C≡C)_*n*_Li Species, and Derivatization with Organic and Inorganic Electrophiles

**DOI:** 10.1021/acs.organomet.4c00098

**Published:** 2024-04-24

**Authors:** Sourajit Dey Baksi, Joshua O. Aggrey, Nattamai Bhuvanesh, John A. Gladysz

**Affiliations:** † Department of Chemistry, Texas A&M University, PO Box 30012, College Station, Texas 77842-3012, United States; ‡ Department of Chemistry, East Tennessee State University, 1276 Gilbreath Drive, Johnson City, Tennessee 37614, United States

## Abstract

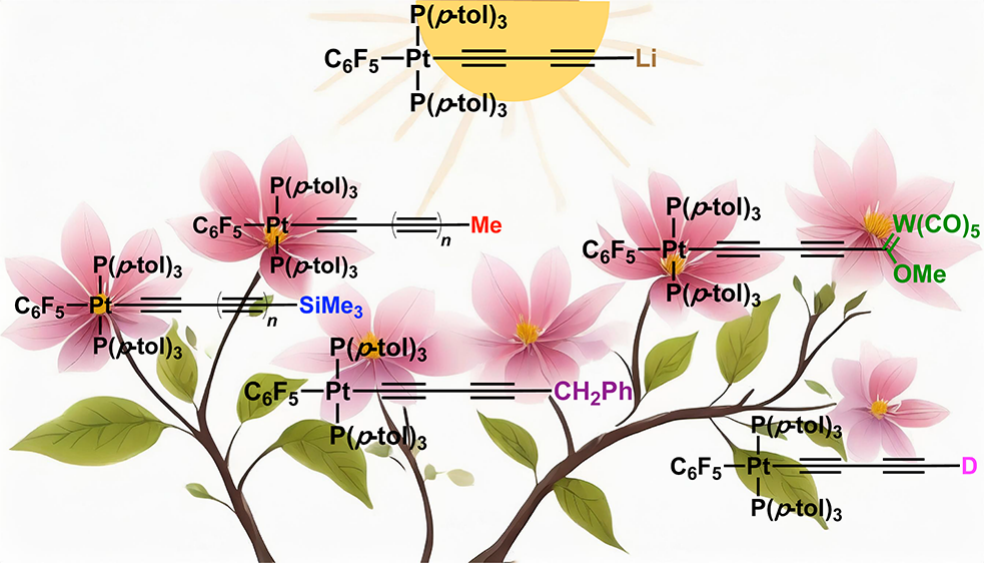

Reactions of the title complexes and *n*-BuLi (1.5 equiv, –45 °C) afford functional equivalents of the deprotonated species *trans*-(C_6_F_5_)(*p*-tol_3_P)_2_Pt(C≡C)_*n*_Li (*n* = 2–4), as assayed by subsequent additions of MeI or Me_3_SiCl to give *trans*-(C_6_F_5_)(*p*-tol_3_P)_2_Pt(C≡C)_*n*_Me (66–52%) or *trans*-(C_6_F_5_)(*p*-tol_3_P)_2_Pt(C≡C)_*n*_SiMe_3_ (63–49%). However, ^31^P NMR data suggest more complicated mechanistic scenarios, and small amounts of the hydride complex *trans*-(C_6_F_5_)(*p*-tol_3_P)_2_PtH (independently synthesized from the chloride complex, AgClO_4_, and NaBH_4_) are detected in most cases. Analogous sequences involving *trans*-(C_6_F_5_)(*p*-tol_3_P)_2_Pt(C≡C)_2_H and benzyl bromide, D_2_O, or W(CO)_6_/Me_3_O^+^ BF_4_^–^ similarly afford products with Pt(C≡C)_2_Bn, Pt(C≡C)_2_D, or Pt(C≡C)_2_C(OCH_3_)=W(CO)_5_ linkages. The crystal structures of the tungsten and corresponding SiMe_3_ adduct, the three Pt(C≡C)_*n*_Me species, and hydride complex are determined.

## Introduction

**Scheme 1:**
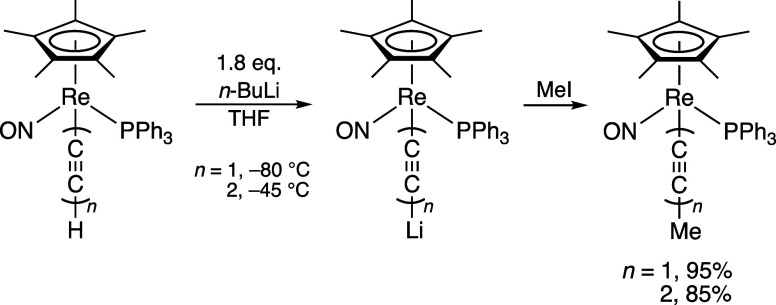
Early Examples of the Deprotonation of Transition Metal Terminal Polyynyl Complexes

The deprotonation of terminal acetylenes to alkali metal acetylides and their subsequent functionalization with electrophiles is one of the workhorse reaction sequences of synthetic organic chemistry.^1^ However, only in the 1990s were such protocols applied to transition metal complexes of terminal polyynyl ligands, L_*y*_M(C≡C)_*n*_H.^2,3^ Some early examples are depicted in Scheme 1.^2^ There is now a substantial body of more recent applications with other metal fragments.^4−6^ Similar deprotonations/functionalizations have been carried out with carbyne complexes of the formula L_*y*_M≡C(C≡C)_*n*_H,^7^ which have odd numbers of sp carbon atoms.

For some time, we have been studying the syntheses, structures, reactivities, and electronic properties of bimetallic polyynediyl complexes of the formula L_*y*_M(C≡C)_*n*_ML_*y*_.^8−10^ One objective has been to access adducts of very long sp carbon chains, for example with *n* > 25 (MC_>50_M). Parallel studies of extended polyynes with organic end groups have been carried out by other researchers.^11,12^ All parties have sought models for the elusive polymeric sp carbon allotrope, carbyne,^13^ and probed numerous physical properties as a function of chain length. Synthetic efforts have utilized various homocoupling and heterocoupling reactions of terminal alkynes. Our most recent endeavors have involved platinum(II) end group of the formula (C_6_F_5_)(*p*-tol_3_P)_2_Pt.^10^

**Scheme 2:**
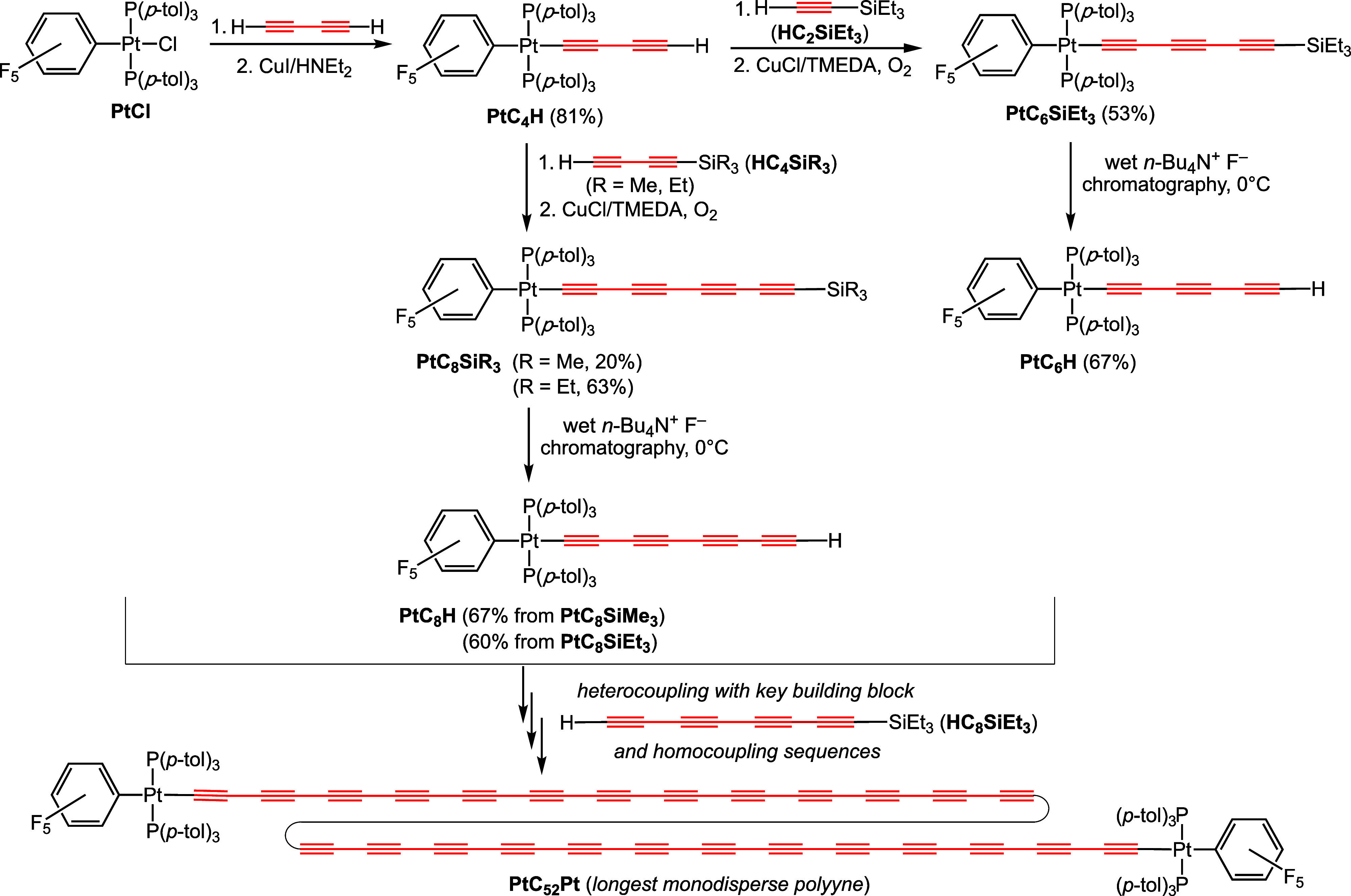
Background Platinum Chemistry; Sources of Key Starting Materials

As shown in Scheme 2, the platinum terminal polyynyl complexes *trans*-(C_6_F_5_)(*p*-tol_3_P)_2_Pt(C≡C)_*n*_H (*n* = 2, **PtC**_**4**_**H**;^10a^ 3, **PtC**_**6**_**H**;^10a^ 4, **PtC**_**8**_**H** can be accessed by condensation of butadiyne and the corresponding platinum chloride (**PtCl**), followed by heterocouplings with simple trialkylsilylalkynes and protodesilylation. These complexes become progressively more demanding to isolate, a trend seen for many series of terminal polyynes, and some previously unreported alternative syntheses are detailed in the Supporting Information. Higher homologues, at least through *n* = 9 or (**PtC**_**18**_**H**), can be generated at low temperature and trapped by click chemistry.^14^ Still higher homologues, such as **PtC**_**26**_**H**, can be trapped by under special oxidative homocoupling conditions to give diplatinum complexes such as **PtC**_**52**_**Pt** (Scheme 2).^10d^

In the course of optimizing routes to **PtC**_**52**_**Pt** and lower homologues, we sought to probe the feasibility of converting the polyynyl complexes **PtC**_*x*_**H** to organolithium derivatives *trans*-(C_6_F_5_)(*p*-tol_3_P)_2_Pt(C≡C)_*n*_Li (*n* = 2, **PtC**_**4**_**Li**; 3, **PtC**_**6**_**Li**; 4, **PtC**_**8**_**Li**). This might open up new families of coupling reactions with potentially improved sp chain extension efficiencies. In this paper, we report that functional equivalents of these targets are easily generated and can be derivatized with a variety of electrophiles to obtain heretofore inaccessible adducts. Crystal structures of several complexes, and some mechanistically intriguing ancillary observations, are also described. No portion of these data have been communicated.

## Results

### 1 Syntheses

**Scheme 3:**
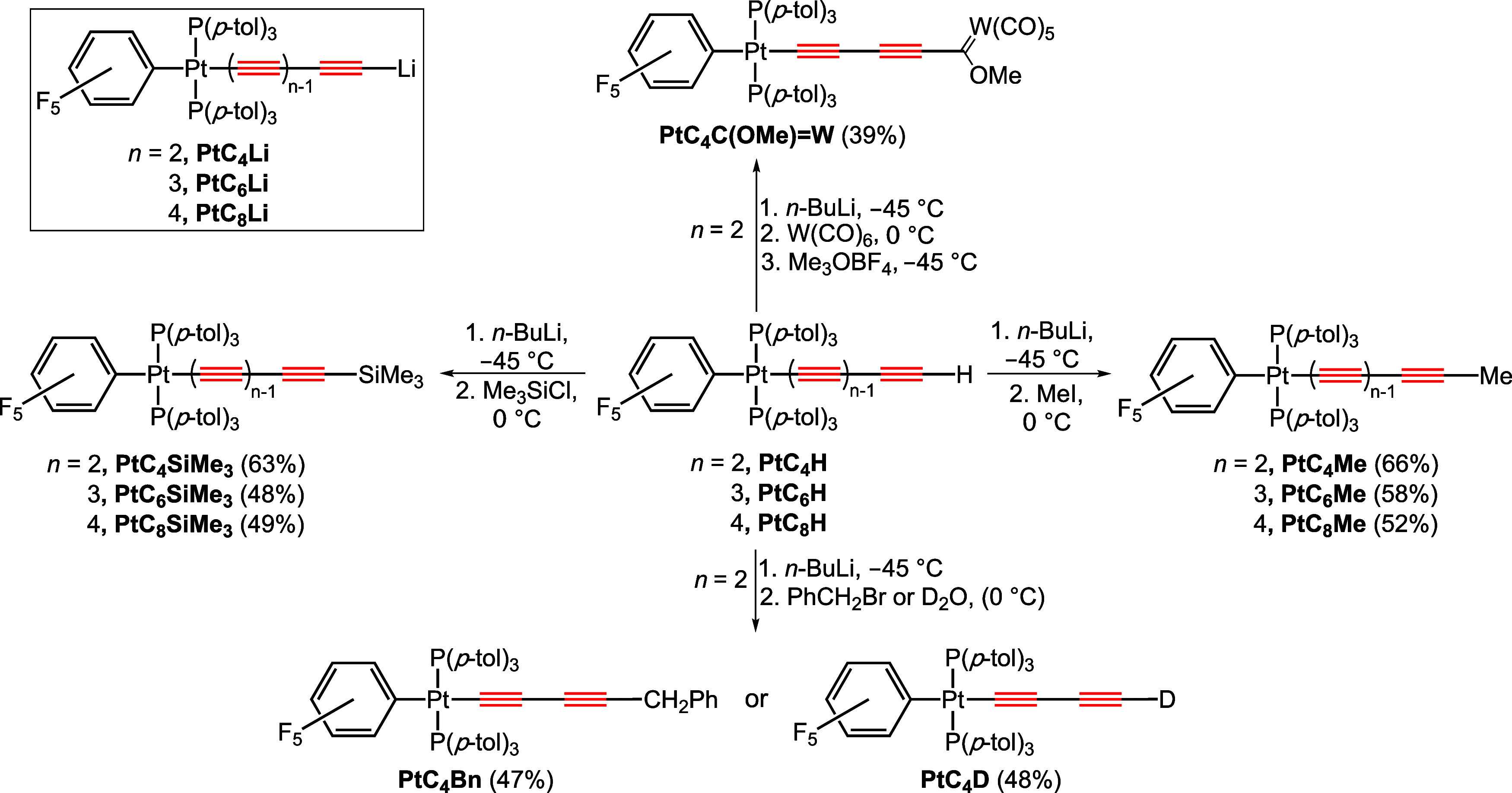
Deprotonation (−45 °C) and Further Reaction (0 °C) of *trans*-(C_6_F_5_)(*p*-tol_3_P)_2_Pt(C≡C)_*n*_H (**PtC**_*x*_**H**; *n* = 2–4)

**Figure 1:**
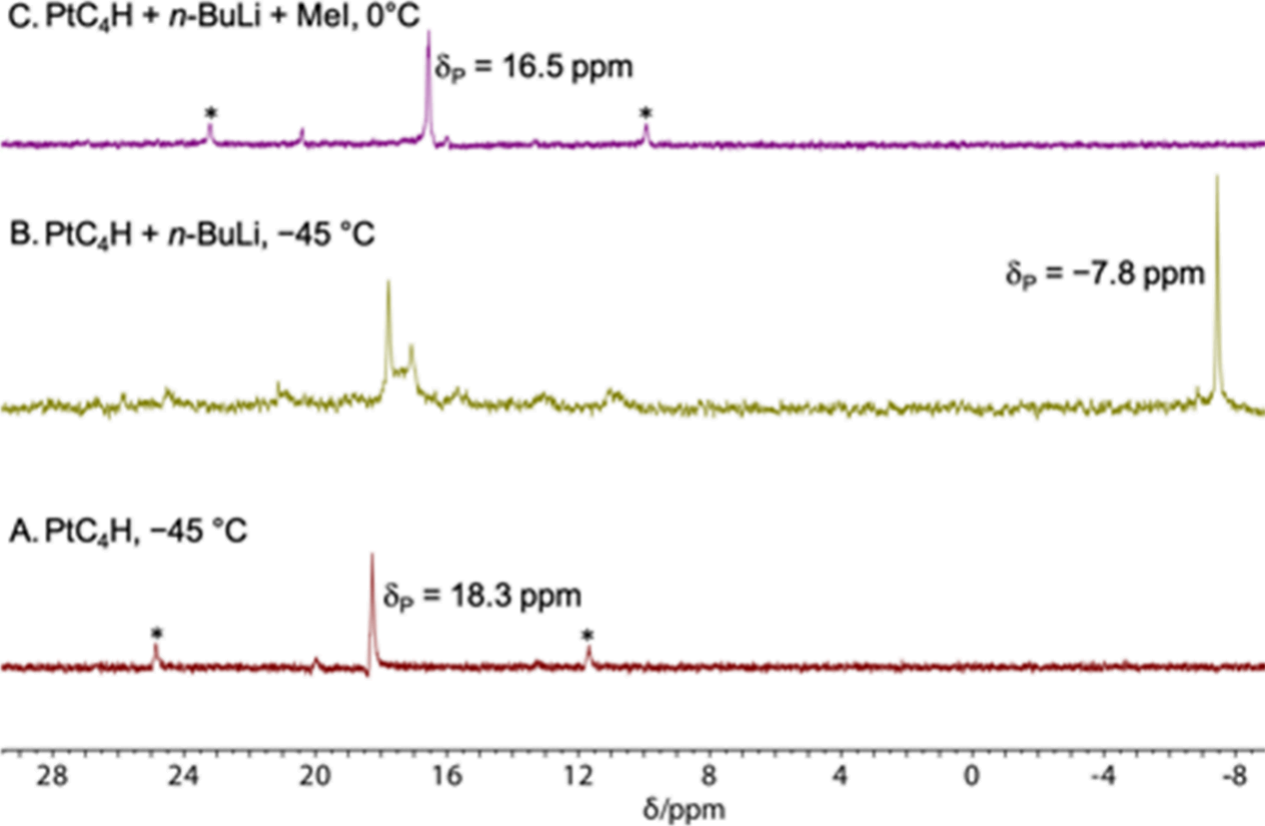
^31^P{^1^H} NMR monitoring of the deprotonation/methylation sequence for **PtC**_**4**_**H** (THF; Scheme 3); * denotes a satellite peak due to ^195^Pt coupling (33.8% abundance). Spectra at additional temperature/time intervals are supplied in Figure S1.

As shown in Scheme 3, a THF solution of **PtC**_**4**_**H** was treated with *n*-BuLi (1.5 equiv, 2.5 M in hexane) at −45 °C. The yellow solution turned orange. As depicted in Figure 1, the ^31^P{^1^H} NMR signal of **PtC**_**4**_**H** (18.3 ppm, ^1^*J*_PPt_ = 2644 Hz) was replaced by (i) a weaker, slightly shifted singlet overlapping with a less intense group of broad signals (17.7–17.1 ppm) immediately upfield, (ii) a sharp upfield signal (−7.8 ppm) noteworthy for the absence of ^195^Pt satellites, and (iii) several minor signals. The solution was then warmed to 0 °C and MeI (1.8 equiv) added. The ^31^P{^1^H} NMR spectrum was now dominated by a new ^195^Pt-coupled signal (16.5 ppm, ^1^*J*_PPt_ = 2684 Hz).

Workup of a similar preparative reaction gave a 63% yield of an air stable white solid with NMR and IR properties, as well as a microanalysis, consistent with the pentadiynyl complex *trans*-(C_6_F_5_)(*p*-tol_3_P)_2_Pt(C≡C)_2_Me (**PtC**_**4**_**Me**; ^31^P{^1^H} NMR (CDCl_3_) 17.2 ppm, ^1^*J*_PPt_ = 2672 Hz). In a one-shot experiment with *t*-BuLi, **PtC**_**4**_**Me** was isolated in 31% yield. These results were taken as evidence for the generation of a functional equivalent of **PtC**_**4**_**Li**, albeit with some complexities as further elaborated below.

Analogous preparative sequences were carried out with isolated **PtC**_**6**_**H**(10a) and **PtC**_**8**_**H**.^10c^ As shown in Scheme 3, **PtC**_**6**_**Me** and **PtC**_**8**_**Me** were obtained in 58–52% yields and similarly characterized. The methyl groups in **PtC**_*x*_**Me** were evidenced by ^1^H NMR signals that shifted downfield with sp chain length (δ 1.62, 1.81, 1.89 ppm, respectively; 3 × s). The structures were further confirmed crystallographically, as described in a following section. However, an analogous sequence involving **PtC**_**10**_**H**, generated in situ under conditions where click trapping is successful,^14^ did not yield detectable quantities of **PtC**_**10**_**Me**.

Next, solutions generated from **PtC**_*x*_**H** and *n*-BuLi at −45 °C were quenched (0 °C) with the silicon electrophile Me_3_SiCl (1.8 equiv). As shown in Scheme 3, *trans*-(C_6_F_5_)(*p*-tol_3_P)_2_Pt(C≡C)_*n*_SiMe_3_ (*n* = 2, **PtC**_**4**_**SiMe**_**3**_; 3, **PtC**_**6**_**SiMe**_**3**_;^10c^ 4, **PtC**_**8**_**SiMe**_**3**_(10c)) were isolated as air stable white to yellow solids in 63–48% yields. The last two complexes have been independently prepared by sequences similar to those in Scheme 2, but the first represents a “missing link”.

Solutions generated from the butadiynyl complex **PtC**_**4**_**H** and *n*-BuLi were similarly treated with other types of electrophiles. In the case of benzyl bromide, workup gave the new compound *trans*-(C_6_F_5_)(*p*-tol_3_P)_2_Pt(C≡C)_2_CH_2_Ph (**PtC**_**4**_**Bn**) in 47% yield. When solutions were quenched with D_2_O (99.9%), **PtC**_**4**_**D** was isolated in 48% yield. Integration of the residual C_4_H ^1^H NMR signal versus the aryl hydrogen atoms and mass spectrometric analyses indicated 85–82% deuterium labeling.

A number of lithiated terminal alkynes RC≡CLi have been shown to add to metal carbonyl complexes, and alkylation of the resulting adducts can afford Fischer carbene complexes.^15^ We have previously reported such sequences starting with the chiral rhenium complexes (η^5^-C_5_Me_5_)Re(NO)(PPh_3_)(C≡C)_*n*_Li (*n* = 1, 2) illustrated in Scheme 1.^16^ Accordingly, solutions generated from **PtC**_**4**_**H** and *n*-BuLi were treated with W(CO)_6_ (0 °C). The orange solution turned purple, and after cooling (−45 °C), Me_3_O^+^ BF_4_^–^ was added. Workup gave the red-brown bimetallic Fischer carbene complex *trans*-(C_6_F_5_)(*p*-tol_3_P)_2_Pt(C≡C)_2_C(OMe)=W(CO)_5_ (**PtC**_**4**_**C(OMe)=W**) in 39% yield. The C=W linkage was evidenced by a ^13^C{^1^H} NMR signal at 289.0 ppm with a ^1^*J*_CW_ value of 112.3 Hz (d, satellites). The CO ligands exhibited resonances at 198.2 ppm (more intense, *cis* to C=W) and 206.4 (*trans* to C=W) with ^1^*J*_CW_ values of 129–123 Hz.

The hexatriynyl and octatetraynyl complexes **PtC**_**6**_**H** and **PtC**_**8**_**H** were analogously reacted with *n*-BuLi, W(CO)_6_, and Me_3_O^+^ BF_4_^–^. NMR analyses of samples that had always been kept at ≤0 °C showed mainly starting material. The Bro̷nsted acidities of terminal polyynes increase with increasing numbers of triple bonds,^17^ so the conjugate bases should become less basic and nucleophilic. Thus, both thermodynamic and kinetic factors may be responsible for the diminished reactivity.

### 2. Mechanistically Relevant Observations

The preparative data in Scheme 3 convincingly establish that some functional equivalent of **PtC**_*x*_**Li** can be generated from **PtC**_*x*_**H** and *n*-BuLi. However, when similar lithiations of the Re(C≡C)_*n*_H species in Scheme 1 or cyclopentadienyl homologues are monitored by ^31^P{^1^H} NMR at −80 °C, the PPh_3_ signals shift 0–2 ppm downfield.^2b,2c^ In some cases, several closely spaced signals result, possibly reflecting different ion pairing or aggregation modes. Bruce has similarly reported that upon lithiation of (η^5^-C_5_Me_5_)Ru(dppe)(C≡C)_2_H, the ^31^P{^1^H} signal shifts downfield by 2 ppm.^6a^ For further calibration, when the cyclopentadienyl ligands of a variety of complexes of the formula (η^5^-C_5_H_5_)Re(NO)(PPh_3_)(X) are monolithiated, the ^31^P{^1^H} signals shift 3–5 ppm downfield.^18^

However, in Figure 1B, most of the new signals are *upfield* from that of **PtC**_**4**_**H**. The group of peaks at 17.7–17.1 ppm could represent unreacted **PtC**_**4**_**H** and/or a set of lithiated species. The sharp upfield singlet at −7.8 ppm, visually judged to be of lesser area, is close to that of the free phosphine *p*-tol_3_P in THF (−8.2 ppm), consistent with the apparent lack of ^195^Pt coupling. Surprisingly, when free *p*-tol_3_P was introduced prior to *n*-BuLi addition, or reaction mixtures were subsequently spiked with *p*-tol_3_P, two ^31^P{^1^H} NMR signals were always observed (e.g., – 8.1 and −7.5 ppm). At all stages, reaction mixtures were homogeneous.

The generation of several phosphorus-containing species was considered. No ^31^P{^1^H} NMR data have been reported for the phosphonium salt derived from *p*-tol_3_P and MeI, *p*-tol_3_PMe^+^ I^–^.^19^ However, the triphenyl analogue Ph_3_PMe^+^ I^–^ exhibits a downfield signal (21.1 ppm, CDCl_3_)^20,21^ far from the −7.8 ppm species. Triarylphosphines can be reduced to alkali metal phosphides by alkyl lithium reagents, but these chemical shifts are far upfield (e.g., Li^+^ Ph_2_P^–^ (C_6_D_6_), – 22.7 ppm^22^). Triarylphosphine oxide signals would be downfield that of **PtC**_**4**_**Me**.^23^ When the reaction in Figure 1 was monitored by ^19^F{^1^H} NMR, some new, slightly shifted signals were apparent but no major changes that might suggest a disrupted C_6_F_5_ ring (Figure S2). Additional analysis is provided in Discussion Section.

During the chromatographic workups of **PtC**_**4**_**Me**, **PtC**_**6**_**Me**, **PtC**_**8**_**Me**, **PtC**_**4**_**Bn**, and **PtC**_**4**_**SiMe**_**3**_, small amounts of a common byproduct eluted after the main product. NMR data suggested that this might be the hydride complex **PtH**, and an isolated yield (11%) was determined for the sequence affording **PtC**_**4**_**Me**. An authentic sample was synthesized from **PtCl**, as shown in eq 1. The complex exhibited a characteristic upfield ^1^H NMR signal (−6.3 ppm, CDCl_3_) and weak IR ν_PtH_ band (2010 cm^–1^). As depicted in Figure S1, a weak ^31^P{^1^H} NMR signal for **PtH** could be detected prior to the isolation of **PtC**_**4**_**Me**. When the reaction of **PtC**_**4**_**H** and *n*-BuLi was simply quenched with methanol, **PtH** was subsequently isolated in 15% yield.^24^ When the reaction was quenched with D_2_O, a ^1^H NMR spectrum showed the **PtH** to be essentially completely protiated.
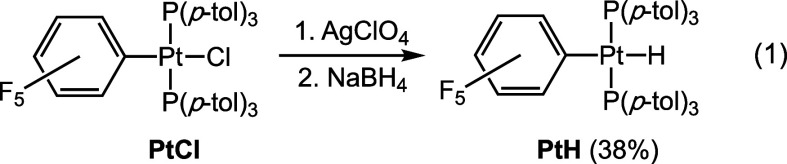
1

### 3. Crystallography

**Figure 2:**
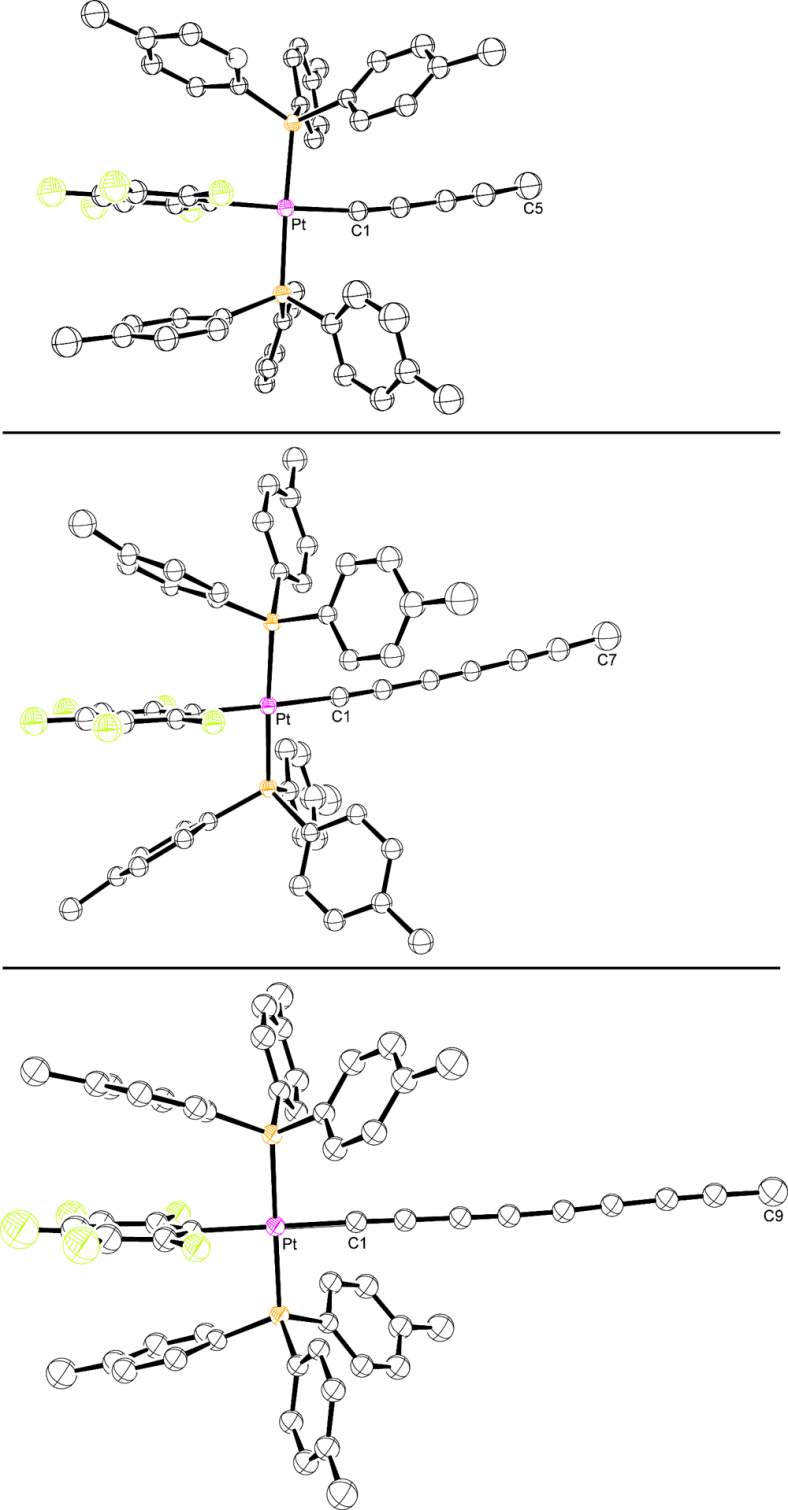
Thermal ellipsoid plots (50% probability level) for **PtC**_**4**_**Me** (top), **PtC**_**6**_**Me**·(CH_2_Cl_2_)_0.08_ (middle), and **PtC**_**8**_**Me** (bottom) with hydrogen atoms and solvent molecules omitted.

Single crystals of the series **PtC**_**4**_**Me**, **PtC**_**6**_**Me**, and **PtC**_**8**_**Me** could be grown. Their structures were solved as outlined in Table S1 and Discussion Section. Thermal ellipsoid diagrams are given in Figure 2, all of which exhibit C_6_H_4_CH_3_/C_6_F_5_/C_6_H_4_CH_3_ π stacking interactions. Key metrical parameters are summarized in Table S3. The average C_6_H_4_CH_3_/C_6_F_5_ centroid/centroid distances (3.60, 3.94, 3.68 Å, respectively) quantify the visually more splayed stacking in **PtC**_**6**_**Me**. Additional features are analyzed in Discussion Section.

**Figure 3:**
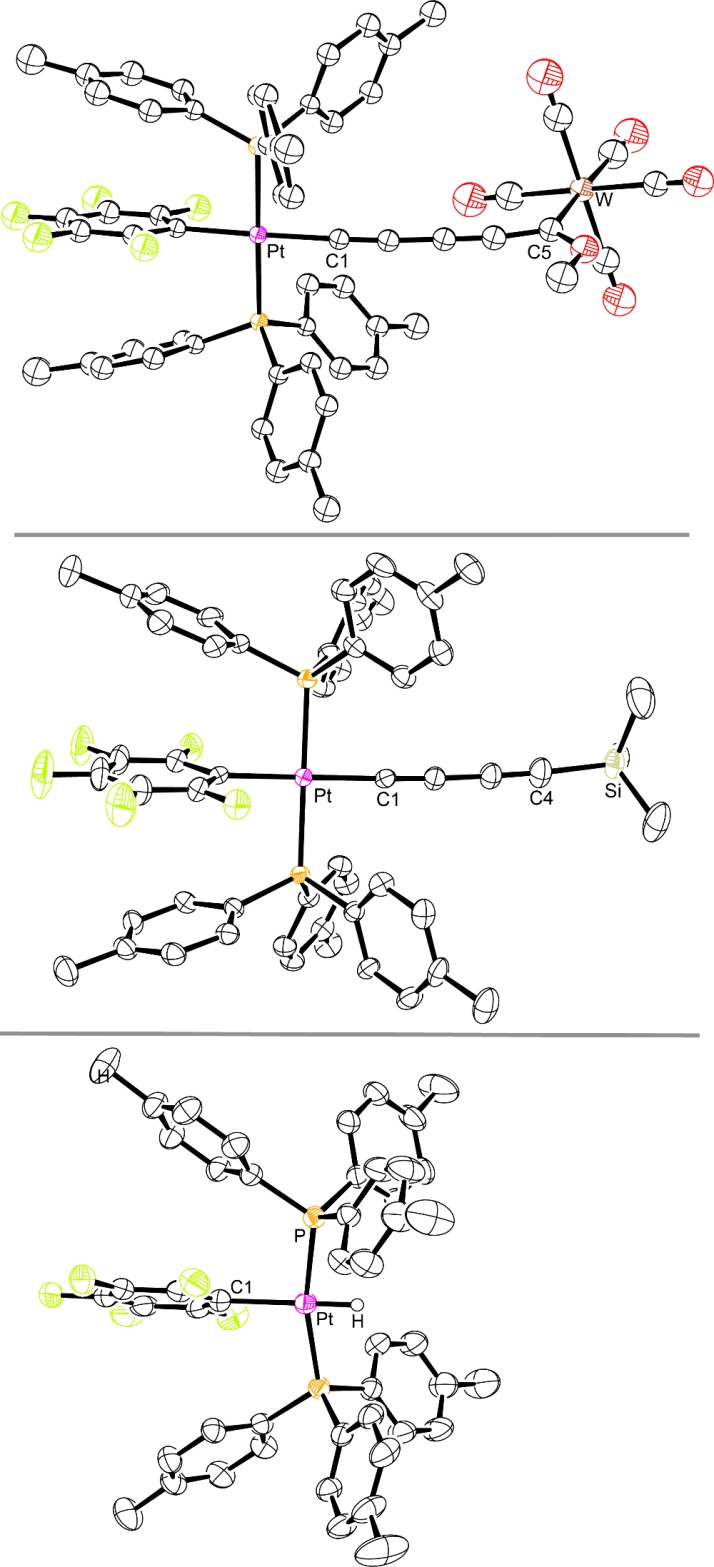
Thermal ellipsoid plots (50% probability level) for **PtC**_**4**_**C(OMe)=W** (top), **PtC**_**4**_**SiMe**_**3**_ (middle, crystal 2), and **PtH**·(CH_2_Cl_2_)_0.6_ (bottom) with carbon-bound hydrogen atoms and solvent molecules omitted. Selected bond lengths/angles (Å/°) for **PtH**·(CH_2_Cl_2_)_0.6_: Pt–H, 1.2421; Pt–P, 2.2746(6); Pt–C1, 2.091(4); P–Pt–P, 166.23(3).

The crystal structures of the inorganic derivatives **PtC**_**4**_**SiMe**_**3**_ and **PtC**_**4**_**C(OMe)=W** were similarly determined. The former was obtained as a solvate from toluene or in unsolvated form from CH_2_Cl_2_/hexane. Two crystals of the unsolvated form were analyzed, and the best of the three structures is depicted in Figure 3. Given the surprise associated with the initial detection of the byproduct **PtH**, it was crystallographically characterized prior to independent synthesis (eq 1). The molecular structure featured a *C*_*2*_ symmetry axis, and some metrical parameters are incorporated into the caption of Figure 3.

## Discussion

Scheme 3 clearly establishes the feasibility of derivatizing **PtC**_**4**_**H**, **PtC**_**6**_**H**, and **PtC**_**8**_**H** with a variety of electrophiles following additions of *n*-BuLi. In principle, it should be possible to directly synthesize the corresponding methylation products **PtC**_*x*_**Me** by Sonogashira-type couplings of **PtCl** with the terminal alkynes H(C≡C)_*n*_Me, analogously to the condensation with 1,3-butadiyne in Scheme 2. However, these alkynes are difficult to access,^25−27^ and we judge it easier to build up the ligands in the metal coordination sphere.

**Scheme 4:**
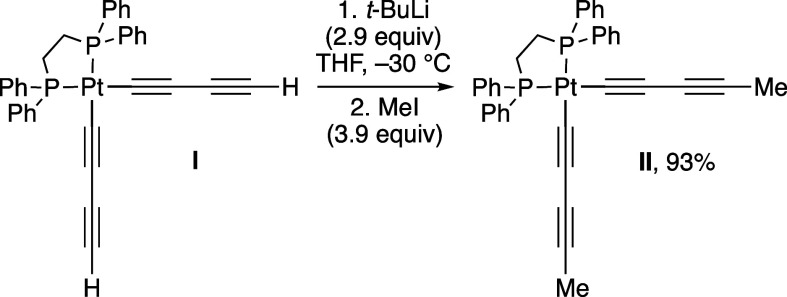
Relevant Literature Data

Bruce has previously reported the similar functionalization of the *cis* bis(1,3-butadiynyl) platinum complex **I** in Scheme 4.^4^ He found that sequential treatment with excess *t*-BuLi and MeI afforded the dimethylated product **II** in 93% yield after workup. However, analogous sequences with Me_3_SiCl and AuCl(PPh_3_) afforded *mono*silyl and *mono*aurated derivatives that retained one (C≡C)_2_H ligand. ^31^P{^1^H} NMR spectra were not recorded during these transformations, but like our experience with Figure 1, might have proved difficult to interpret.

Individually, the crystal structures of **PtC**_**4**_**Me**, **PtC**_**6**_**Me**, and **PtC**_**8**_**Me** are routine, with bond lengths, bond angles, and arene/arene stacking interactions similar to many other alkynyl and polyynyl adducts of *trans*-(C_6_F_5_)(*p*-tol_3_P)_2_Pt.^10,14^ In accordance with computational predictions^28^ and experimental analyses,^29^ the Pt–C≡ bond lengths contract from 2.009(2) to 1.983(3) Å. In principle, the carbon–carbon bond lengths should also exhibit monotonic trends, but as is often the case with lighter atoms, the ESD values are too high for rigorous conclusions. In **PtC**_**4**_**C(OMe)=W**, the bond lengths involving the atoms between platinum and tungsten are potentially influenced by the zwitterionic resonance form ^+^M(=C=C)_*n*_=C(OMe)-W(CO)_5_^–^. However, the Pt–C≡ bond length (2.003(5) Å) is close to that of **PtC**_**4**_**Me**. In contrast, the W=C bond (2.147(5) Å) is slightly shorter than in (η^5^-C_5_Me_5_)Re(NO)(PPh_3_)C≡CC(OMe)=W(CO)_5_ (2.200(8) Å),^16^ which is anchored by a π basic rhenium fragment that should enhance zwitterionic character and reduce the tungsten–carbon bond order.

As noted above, ^31^P{^1^H} NMR data for the family of rhenium complexes in Scheme 1(2) and related ruthenium complexes of Bruce et al.^6a^ suggest that ancillary phosphine ligands in MC_4_Li species should have chemical shifts similar to those of MC_4_H analogues, or a few ppm downfield. No such downfield signals are apparent in Figure 1. However, since the literature data are derived from 18-valence-electon octahedral complexes, it may not be unreasonable that resonances associated with 16-valence-electron square planar **PtC**_**4**_**Li** are among the slightly upfield group of signals at 17.7–17.1 ppm. An “ate complex” derived from *n*-BuLi addition to platinum has been considered, but this has little precedent.^30^ Nucleophilic aromatic substitutions involving fluoroarenes have abundant precedent, but species derived from addition are only rarely observable^31^ and our ^19^F{^1^H} spectra (Figure s2) show only sp^2^ CF signals.^32^

The formation of the byproduct **PtH** in so many of the preceding reactions also poses a puzzle. However, in syntheses of extended polyynes by certain types of oxidative cross- and homocoupling reactions, the loss of C_2_ units is sometimes observed.^11,14,33^ There are currently no rationales for these well-documented minor reaction pathways, which are quite possibly related. In the same vein, we presently have no explanation for the generation and then disappearance of the −7.8 ppm ^31^P{^1^H} NMR signal in Figure 1 (which seems not to be *p*-tol_3_P).

In an effort to further extend this chemistry, the reaction mixtures generated from **PtC**_*x*_**H** and *n*-BuLi were treated with various one-electron oxidants. It was hoped that homocouplings to diplatinum complexes **PtC**_*2x*_**Pt** might be effected. However, complex product mixtures were produced. Nonetheless, transmetalation chemistry remains worthy of exploration. For example, the rhenium analogues (η^5^-C_5_Me_5_)Re(NO)(PPh_3_)(C≡C)_*n*_Li undergo Li/Cu exchange to give species that efficiently condense with brominated alkynes and diynes.^8^ Thus, the deprotonation products of **PtC**_*x*_**H** continue to have considerable promise for sp chain elongation protocols.

In conclusion, this study has extended a class of reactions that we developed for octahedral rhenium terminal polyynyl complexes in 1991 (Scheme 1) to square planar platinum terminal polyynyl complexes *trans*-(C_6_F_5_)(*p*-tol_3_P)_2_Pt(C≡C)_*n*_H (*n* = 2–4; Scheme 3). In the intervening 33 years, Bruce and several additional groups have reported reactions of other L_*y*_M(C≡C)_*n*_H species and strong (usually RLi) bases that generate functional equivalents of the conjugate bases.^4−6^ These can be derivatized by a variety of electrophiles and have promise for various heterocoupling and oxidative homocoupling reactions. Despite the occasional mechanistic puzzle, such sequences have much potential for synthetic organometallic chemistry and continue to receive attention in this research group.

## Experimental Section

All instrumentation and characterization protocols were identical to those in recent full papers in this series.^10c,10,14^ These are summarized, together with chemical sourcing and purification, in the Supporting Information. All reactions were conducted under dry inert atmospheres using conventional Schlenk techniques, but workups were carried out in air.

### *trans*-(C_6_F_5_)(*p*-tol_3_P)_2_Pt(C≡C)_2_Me (PtC_4_Me)

A Schlenk flask was charged with **PtC**_**4**_**H** (0.148 g, 0.145 mmol)^10a^ and THF (30 mL) and cooled to −45 °C (dry ice/acetonitrile). Then, *n*-BuLi (0.087 mL, 2.5 M in hexanes, 0.22 mmol) was added dropwise with stirring. The yellow solution turned orange. After 30 min, the −45 °C bath was replaced by an ice bath. After 15 min, MeI (0.016 mL, 0.26 mmol) was added. After 16 h, the volatiles were removed by oil-pump vacuum and MeOH (10 mL) was added. The creamy white solid was collected by filtration and chromatographed on a silica gel column (3 × 20 cm, packed in hexanes, eluted with a 0:5 → 1:5 v/v CH_2_Cl_2_/hexanes gradient). The solvent was removed from the product-containing fractions by oil-pump vacuum to give **PtC**_**4**_**Me** as an off-white solid (0.098 g, 0.095 mmol, 66%), which started to blacken at 93 °C and melted at 117 °C (open capillary). Anal. calcd for C_53_H_45_F_5_P_2_Pt (1033.94): C, 61.51; H, 4.35. Found: C, 61.73; H, 4.23.

NMR (δ/ppm, CDCl_3_): ^1^H (500 MHz, cryoprobe) 7.54–7.50 (m, 12H, *o* to P),^34^ 7.11 (d, ^3^*J*_HH_ = 10 Hz, 12H, *m* to P),^34^ 2.35 (s, 18H, C*H*_3_, *p* to P), 1.62 (s, 3H, ≡CC*H*_3_); ^13^C{^1^H} (126 MHz, cryoprobe)^35^ 145.7 (dm, ^1^*J*_CF_ = 228 Hz, *o* to Pt), 139.7 (s, *p* to P), 138.0 (dm, *p* to Pt), 135.7 (dm, *m* to Pt), 133.8 (virtual t, ^2^*J*_CP_ = 13 Hz,^36^*o* to P), 127.9 (virtual t, ^3^*J*_CP_ = 11 Hz,^36^*m* to P), 125.8 (virtual t, ^1^*J*_CP_ = 31 Hz,^36^*i* to P), 96.4 (s, ^2^*J*_CPt_ = 264 Hz,^37^ PtC ≡ *C*), 94.1 (s, ^1^*J*_CPt_ = 982 Hz,^37^ Pt*C* ≡ C), 68.7, 67.9 (2 s, PtC≡C*C* ≡ *C*), 21.5 (s, *C*H_3_, *p* to P), 4.8 (s, ≡C*C*H_3_); ^31^P{^1^H} (202 MHz) 17.2 (s, ^1^*J*_PPt_ = 2672 Hz);^37^^31^P{^1^H} (THF, 202 MHz, 0 °C) 16.5 (s, ^1^*J*_PPt_ = 2674 Hz).^37^ IR (powder film, cm^–1^) 2120/2087/2023 (w/m/s, ν_C≡C_). UV–vis (nm, 2.21 × 10^–6^ M in CH_2_Cl_2_, (ε, M^–1^cm^–1^)) 299 (21100), 320 (49000).

### *trans*-(C_6_F_5_)(*p*-tol_3_P)_2_Pt(C≡C)_2_SiMe_3_ (PtC_4_SiMe_3_)

**PtC**_**4**_**H** (0.125 g, 0.123 mmol),^10a^ THF (30 mL), *n*-BuLi (0.074 mL, 2.5 M in hexanes, 0.185 mmol), and Me_3_SiCl (0.028 mL, 0.22 mmol) were combined in a procedure analogous to that for **PtC**_**4**_**Me**. A nearly identical workup (0:6 → 1:6 v/v CH_2_Cl_2_/hexanes gradient) gave **PtC**_**4**_**SiMe**_**3**_ as an off-white solid (0.084 g, 0.077 mmol, 63%), mp 119 °C (open capillary). Anal. calcd for C_55_H_51_F_5_P_2_PtSi (1092.12): C, 60.49; H, 4.71. Found: C, 60.72; H, 4.76.

NMR (δ/ppm, CD_2_Cl_2_): ^1^H (500 MHz, cryoprobe) 7.52–7.48 (m, 12H, *o* to P),^34^ 7.18 (d, ^3^*J*_HH_ = 10 Hz, 12H, *m* to P),^34^ 2.38 (s, 18H, C*H*_3_, *p* to P), −0.01 (s, 9H, Si(C*H*_3_)_3_); ^13^C{^1^H} (126 MHz, cryoprobe)^35^ 146.3 (dm, ^1^*J*_CF_ = 220 Hz, *o* to Pt), 141.6 (s, *p* to P), 138.0 (dm, *p* to Pt), 136.0 (dm, *m* to Pt), 134.7 (virtual t, ^2^*J*_CP_ = 13 Hz,^36^*o* to P), 129.1 (virtual t, ^3^*J*_CP_ = 11 Hz,^36^*m* to P), 127.7 (virtual t, ^1^*J*_CP_ = 31 Hz,^36^*i* to P), 101.6 (br s, ^1^*J*_CPt_ = 999 Hz,^37^ Pt*C*≡C), 96.2 (s, ^2^*J*_CPt_ = 264 Hz,^37^ PtC≡*C*), 92.9 (s, ^2^*J*_CSi_ = 5 Hz,^37^*C*≡CSi), 77.4 (s, ^2^*J*_CSi_ = 32 Hz,^37^ C≡*C*Si), 21.3 (s, *C*H_3_, *p* to P), −0.09 (s, ^1^*J*_SiC_ = 56 Hz,^37^ Si(C*H*_3_)_3_); ^31^P{^1^H} (202 MHz) 16.9 (s, ^1^*J*_PPt_ = 2658 Hz).^37^ IR (powder film, cm^–1^) 2132/2081/2037 (w/m/s, ν_C≡C_). UV–vis (nm, 2.18 × 10^–6^ M in CH_2_Cl_2_, (ε, M^–1^cm^–1^)) 260 (18900), 296 (40300), 324 (51100).

### *trans*-(C_6_F_5_)(*p*-tol_3_P)_2_Pt(C≡C)_2_D (PtC_4_D)

**PtC**_**4**_**H** (0.103 g, 0.101 mmol),^10a^ THF (30 mL), *n*-BuLi (0.061 mL, 2.5 M in hexanes, 0.15 mmol), and D_2_O (0.0033 mL, 0.18 mmol) were combined in a procedure analogous to that for **PtC**_**4**_**Me**. A nearly identical workup (0:4 → 1:4 v/v CH_2_Cl_2_/hexanes gradient) gave **PtC**_**4**_**D** as an off-white solid (0.049 g, 0.048 mmol, 48%).

NMR (δ/ppm, CDCl_3_): ^1^H (500 MHz, cryoprobe) 7.51–7.47 (m, 12H, *o* to P),^34^ 7.11 (d, ^3^*J*_HH_ = 10 Hz, 12H, *m* to P),^34^ 2.35 (s, 18H, C*H*_3_, *p* to P), 1.47 (s, ∼0.15 H, residual ≡C*H*); ^13^C (126 MHz)^35,38^ 145.8 (dm, ^1^*J*_CF_ = 232 Hz, *o* to Pt), 140.8 (s, *p* to P), 137.5 (dm, *p* and *m* to Pt), 134.5 (d, ^1^*J*_CH_ = 163 Hz, *o* to P), 128.8 (d, ^1^*J*_CH_ = 159 Hz, *m* to P), 127.5 (m, overlapping upfield line of preceding signal, *i* to P), 95.1 (s, PtC≡*C*), 72.3 (s, PtC≡C*C*≡C), 59.7 (br, ^1^*J*_CH_ ≅ 250 Hz, ∼ 84:16 ≡*C*D/≡*C*H), 21.5 (q, ^1^*J*_CH_ = 381 Hz, *C*H_3_, *p* to P); ^13^C{^1^H} (126 MHz, cryoprobe)^35^ 146.1 (dm, ^1^*J*_CF_ = 226 Hz, *o* to Pt), 140.9 (s, *p* to P), 137.8 (dm, *p* to Pt), 135.5 (dm, *m* to Pt), 134.5 (virtual t, ^2^*J*_CP_ = 13 Hz,^36^*o* to P), 128.7 (virtual t, ^3^*J*_CP_ = 12 Hz,^36^*m* to P), 127.6 (virtual t, ^1^*J*_CP_ = 30 Hz,^36^*i* to P), 98.1 (br s, Pt*C*≡C),^39^ 95.2 (s, PtC≡*C*), 68.1 (s, PtC≡CC≡C), 59.7 (s, PtC≡CC≡*C*), 21.1 (s, *C*H_3_); ^31^P{^1^H} (202 MHz) 17.3 (s, ^1^*J*_PPt_ = 2652 Hz);^37^ mass spectrum (ESI^+^, m/*Z*, most intense peak of the isotope envelope): 1054 (unknown, 100%), 1039 ([**PtC**_**4**_**D** + NH_4_]^+^), 30%), 1022 ([**PtC**_**4**_**D** + H]^+^), 30%), 970 ([(tol_3_P)_2_Pt(C_6_F_5_)]^+^, 86%).

### *trans*-(C_6_F_5_)(*p*-tol_3_P)_2_Pt(C≡C)_2_CH_2_Ph (PtC_4_Bn)

**PtC**_**4**_**H** (0.133 g, 0.130 mmol),^10a^ THF (30 mL), *n*-BuLi (0.078 mL, 2.5 M in hexanes, 0.195 mmol), and PhCH_2_Br (0.028 mL, 0.23 mmol) were combined in a procedure analogous to that for **PtC**_**4**_**Me**. An identical workup gave **PtC**_**4**_**Bn** as a yellow solid (0.068 g, 0.061 mmol, 47%), mp 129 °C (open capillary). Anal. calcd for C_59_H_49_F_5_P_2_Pt (1110.07): C, 63.84; H, 4.45. Found: C, 63.04; H, 4.36.^40^

NMR (δ/ppm, CDCl_3_): ^1^H (500 MHz, cryoprobe) 7.50–7.47 (m, 12H, *o* to P),^34^ 7.31–7.29 (m, 3H, *m* and *p* to CH_2_), 7.15–7.09 (m, 14H, *m* to P, *o* to CH_2_),^34^ 5.39 (s, 2H, ≡CC*H*_2_), 2.34 (s, 18H, C*H*_3_, *p* to P); ^13^C{^1^H} (126 MHz, cryoprobe)^35^ 145.9 (dm, ^1^*J*_CF_ = 227 Hz, *o* to Pt), 140.9 (s, *p* to P), 137.7 (dm, *p* to Pt), 135.9 (dm, *m* to Pt), 134.5 (virtual t, ^2^*J*_CP_ = 13 Hz,^36^*o* to P), 132.7 (s, *i* to CH_2_), 129.2, 128.9, 128.1 (3 s, *o*/*m*/*p* to CH_2_), 128.8 (virtual t, ^3^*J*_CP_ = 11 Hz,^36^*m* to P), 127.5 (virtual t, ^1^*J*_CP_ = 30 Hz,^36^*i* to P), 106.6 (br s, Pt*C*≡C),^39^ 95.1 (s, PtC≡*C*), 82.1, 58.8 (2 s, PtC≡C*C*≡*C*), 54.2 (s, ≡CCH_2_), 21.5 (s, *C*H_3_, *p* to P); ^31^P{^1^H} (202 MHz) 17.8 (s, ^1^*J*_PPt_ = 2646 Hz).^37^ IR (powder film, cm^–1^) 2120/2087/2054/2023 (w/w/m/w, ν_C≡C_). UV–vis (nm, 2.06 × 10^–6^ M in CH_2_Cl_2_, (ε, M^–1^cm^–1^)) 260 (37800), 278 (51300), 318 (54600).

### *trans*-(C_6_F_5_)(*p*-tol_3_P)_2_Pt(C≡C)_2_C(OCH_3_)=W(CO)_5_ (PtC_4_C(OMe)=W)

**PtC**_**4**_**H** (0.178 g, 0.175 mmol),^10a^ THF (30 mL), *n*-BuLi (0.105 mL, 2.5 M in hexanes, 0.26 mmol), and W(CO)_6_ (0.101 g, 0.289 mmol) were combined in a procedure analogous to that for **PtC**_**4**_**Me**. After 45 min, the purple solution was cooled to −45 °C. A Schlenk flask was charged with Me_3_O^+^ BF_4_^–^ (0.043 g, 0.29 mmol) and cooled −45 °C (dry ice/acetonitrile). The purple solution was transferred to the Schlenk flask by cannula with stirring. After 1 h, the cold bath was removed. After 16 h, the volatiles were removed by oil-pump vacuum. The residue was chromatographed on a Florisil column (3 × 20 cm, packed in hexanes under nitrogen, eluted with a 0:3 → 1:3 v/v CH_2_Cl_2_/ hexanes gradient). The solvent was removed from the product-containing fraction by oil-pump vacuum to give **PtC**_**4**_**C(OMe)=W** as a red-brown oil that solidified to a red-orange powder at −35 °C overnight (0.095 g, 0.069 mmol, 39%) and decomposed at 112 °C without melting (sealed capillary). Anal. calcd for C_59_H_45_F_5_O_6_P_2_PtW (1385.84): C, 51.13; H, 3.27. Found: C, 51.47; H, 3.39.

NMR (δ/ppm, C_6_D_6_): ^1^H (500 MHz, cryoprobe) 7.77–7.73 (m, 12H, *o* to P),^34^ 6.95 (d, ^3^*J*_HH_ = 10 Hz, 12H, *m* to P),^34^ 3.92 (s, 3H, OC*H*_3_), 2.32 (s, 18H, C*H*_3_, *p* to P); ^13^C{^1^H} (126 MHz, cryoprobe)^35^ 289.0 (s, ^1^*J*_CW_ = 112 Hz,^37^ C=W), 206.4 (s, ^1^*J*_CW_ = 123 Hz,^37^ CO_*trans*_), 198.2 (s, ^1^*J*_CW_ = 129 Hz,^37^ CO_*cis*_), 145.7 (dm, ^1^*J*_CF_ = 226 Hz, *o* to Pt), 139.8 (s, *p* to P), 137.9 (dm, *p* to Pt), 135.9 (dm, *m* to Pt), 133.5 (virtual t, ^2^*J*_CP_ = 12 Hz,^36^*o* to P), 127.5 (virtual t, ^3^*J*_CP_ = 10 Hz,^36^*m* to P), 125.6 (virtual t, ^1^*J*_CP_ = 31 Hz,^36^*i* to P), 96.9 (br s, Pt*C*≡C),^39^ 78.0 (s, PtC≡*C*), 63.6, 57.2 (2 s, PtC≡C*C*≡*C*), 52.1 (s, O*C*H_3_), 23.5 (s, *C*H_3_, *p* to P); ^31^P{^1^H} (202 MHz) 17.5 (s, ^1^*J*_PPt_ = 2722 Hz).^37^ IR (powder film, cm^–1^) 2127/2085/2055/2044/2021 (w/w/s/m/w, ν_C≡O_ and ν_C≡C_). UV–vis (nm, 2.21 × 10^–6^ M in CH_2_Cl_2_, (ε, M^–1^cm^–1^)) 356 (162000), 451 (228000), 466 (312000).

### *trans*-(C_6_F_5_)(*p*-tol_3_P)_2_Pt(C≡C)_3_Me (PtC_6_Me)

**PtC**_**6**_**H** (0.121 g, 0.116 mmol), THF (30 mL), *n*-BuLi (0.069 mL, 2.5 M in hexanes, 0.17 mmol), and MeI (0.013 mL, 0.21 mmol) were combined in a procedure analogous to that for **PtC**_**4**_**Me**. An identical workup gave **PtC**_**6**_**Me** as an off-white solid (0.071 g, 0.067 mmol, 58%) that started to blacken at 97 °C and melted at 120 °C (open capillary). Anal. calcd for C_55_H_45_F_5_P_2_Pt (1057.94): C, 62.44; H, 4.29. Found: C, 62.29; H, 4.22.

NMR (δ/ppm, CDCl_3_): ^1^H (500 MHz, cryoprobe) 7.50–7.46 (m, 12H, *o* to P),^34^ 7.11 (d, ^3^*J*_HH_ = 10 Hz, 12H, *m* to P),^34^ 2.36 (s, 18H, C*H*_3_, *p* to P), 1.81 (s, 3H, ≡CC*H*_3_); ^13^C{^1^H} (126 MHz, cryoprobe)^35^ 146.1 (dm, ^1^*J*_CF_ = 224 Hz, *o* to Pt), 140.9 (s, *p* to P), 138.0 (dm, *p* to Pt), 135.8 (dm, *m* to Pt), 134.4 (virtual t, ^2^*J*_CP_ = 13 Hz,^36^*o* to P), 128.8 (virtual t, ^3^*J*_CP_ = 11 Hz,^36^*m* to P), 127.4 (virtual t, ^1^*J*_CP_ = 31 Hz,^36^*i* to P), 100.6 (br s, ^1^*J*_CPt_ = 998 Hz,^37^ Pt*C*≡C), 95.8 (s, PtC≡*C*), 72.4, 66.2, 64.1, 56.0 (4 s, PtC≡C*C*≡*CC*≡*C*), 21.3 (s, *C*H_3_, *p* to P), 4.7 (s, ≡C*C*H_3_); ^31^P{^1^H} (202 MHz) 17.4 (s, ^1^*J*_PPt_ = 2642 Hz).^37^ IR (powder film, cm^–1^) 2167/2083/2065/2051/2019 (w/w/s/m/w, ν_C≡C_).

### *trans*-(C_6_F_5_)(*p*-tol_3_P)_2_Pt(C≡C)_3_SiMe_3_ (PtC_6_SiMe_3_)^10c^

**PtC**_**6**_**H** (0.151 g, 0.145 mmol), THF (30 mL), *n*-BuLi (0.087 mL, 2.5 M in hexanes, 0.22 mmol), and Me_3_SiCl (0.033 mL, 0.26 mmol) were combined in a procedure analogous to that for **PtC**_**4**_**Me**. A nearly identical workup (0:6 → 1:6 v/v CH_2_Cl_2_/hexanes gradient) gave **PtC**_**6**_**SiMe**_**3**_ as an off-white solid (0.077 g, 0.069 mmol, 48%). The NMR data agreed with those reported previously.^10c^

### *trans*-(C_6_F_5_)(*p*-tol_3_P)_2_Pt(C≡C)_4_Me (PtC_8_Me)

**PtC**_**8**_**H** (0.109 g, 0.102 mmol), THF (30 mL), *n*-BuLi (0.061 mL, 2.5 M in hexanes, 0.15 mmol), and MeI (0.011 mL, 0.18 mmol) were combined in a procedure analogous to that for **PtC**_**4**_**Me**. An identical workup gave **PtC**_**8**_**Me** as a pale-yellow solid (0.057 g, 0.053 mmol, 52%), which started to blacken at 94 °C and melted at 122 °C (open capillary). Anal. calcd for C_57_H_45_F_5_P_2_Pt (1082.01): C, 63.27; H, 4.19. Found: C, 62.93; H, 4.21.

NMR (δ/ppm, CDCl_3_): ^1^H (500 MHz, cryoprobe) 7.47–7.44 (m, 12H, *o* to P),^34^ 7.11 (d, ^3^*J*_HH_ = 10 Hz, 12H, *m* to P),^34^ 2.36 (s, 18H, C*H*_3_, *p* to P), 1.89 (s, 3H, ≡CC*H*_3_); ^13^C{^1^H} (126 MHz, cryoprobe)^35^ 146.1 (dm, ^1^*J*_CF_ = 225 Hz, *o* to Pt), 141.1 (s, *p* to P), 138.0 (dm, *p* to Pt), 135.9 (dm, *m* to Pt), 134.4 (virtual t, ^2^*J*_CP_ = 13 Hz,^36^*o* to P), 128.9 (virtual t, ^3^*J*_CP_ = 11 Hz,^36^*m* to P), 127.2 (virtual t, ^1^*J*_CP_ = 31 Hz,^36^*i* to P), 104.7 (br s, Pt*C*≡C),^39^ 95.6 (s, PtC≡*C*), 74.3, 65.9, 65.3, 62.1, 59.8, 56.7 (6 s, PtC≡C*C*≡*CC*≡*CC*≡*C*), 21.5 (s, *C*H_3_, *p* to P), 4.8 (s, ≡C*C*H_3_); ^31^P{^1^H} (202 MHz) 17.4 (s, ^1^*J*_PPt_ = 2642 Hz).^37^ IR (powder film, cm^–1^) 2159/2073/2069/2054/2009 (w/w/s/m/w, ν_C≡C_).

### *trans*-(C_6_F_5_)(*p*-tol_3_P)_2_Pt(C≡C)_4_SiMe_3_ (PtC_8_SiMe_3_)^10c^

**PtC**_**8**_**H** (0.106 g, 0.098 mmol), THF (30 mL), *n*-BuLi (0.059 mL, 2.5 M in hexanes, 0.15 mmol), and Me_3_SiCl (0.022 mL, 0.18 mmol) were combined in a procedure analogous to that for **PtC**_**4**_**Me**. A nearly identical workup (0:6 → 1:6 v/v CH_2_Cl_2_/hexanes gradient) gave **PtC**_**8**_**SiMe**_**3**_ as a yellow solid (0.055 g, 0.048 mmol, 49%). The NMR data agreed with those reported previously.^10c^

### *trans*-(C_6_F_5_)(*p*-tol_3_P)_2_PtH (PtH)

A Schlenk flask was charged with **PtCl** (0.114 g, 0.113 mmol)^10a^ and CH_2_Cl_2_ (20 mL) and shielded from light with aluminum foil. A solution of AgClO_4_ (0.023 g, 0.113 mmol) in methanol (10 mL) was added dropwise with stirring. After 24 h, the mixture was filtered to remove the presumed AgCl. The filtrate was transferred by cannula to another Schlenk flask that had been precooled at 0 °C. Solid NaBH_4_ (0.017 g, 0.452 mmol) was added with stirring. After 1 h, the cold bath was removed and the volatiles removed by oil-pump vacuum. The resulting oil solidified over a period of 16 h at 4 °C. The solid was recrystallized from CH_2_Cl_2_/methanol to give **PtH** (0.042 g, 0.043 mmol, 38%) as off-white flakes, which decomposed at 122 °C without melting (closed capillary). Anal. calcd for C_48_H_43_F_5_P_2_Pt (971.89): C, 59.32; H, 4.46. Found: C, 59.01; H, 4.28.

NMR (δ/ppm, CDCl_3_): ^1^H (500 MHz, cryoprobe) 7.46–7.45 (m, 12H, *o* to P),^34^ 7.08 (d, ^3^*J*_HH_ = 10 Hz, 12H, *m* to P),^34^ 2.35 (s, 18H, C*H*_3_, *p* to P), −6.3 (apparent nonet with apparent *J* = 10 Hz; ^1^*J*_HPt_ = 725 Hz,^37^ 1H, Pt*H*); ^13^C{^1^H} (126 MHz, cryoprobe)^35^ 145.9 (dm, ^1^*J*_CF_ = 224 Hz, *o* to Pt), 140.5 (s, *p* to P), 137.4 (dm, *p* to Pt), 135.4 (dm, *m* to Pt), 134.2 (virtual t, ^2^*J*_CP_ = 14 Hz,^36^*o* to P), 130.0 (virtual t, ^1^*J*_CP_ = 58 Hz,^36^*i* to P), 128.8 (virtual t, ^3^*J*_CP_ = 11 Hz,^36^*m* to P), 21.3 (s, *C*H_3_, *p* to P); ^31^P{^1^H} (202 MHz) 28.1 (s, ^1^*J*_PPt_ = 2961 Hz).^37^ IR (powder film, cm^–1^) 2010 (w, ν_PtH_).

### Crystallography

The following structure solution is representative, and others are detailed in the SI. A CH_2_Cl_2_ solution of **PtC**_**4**_**Me** was layered with hexanes and kept at 4 °C. After 3 days, colorless blocks were collected. Cell parameters were determined from 60 data frames taken at widths of 0.5° and refined with 111,645 reflections using CrysAlisPro.^41^ Numerical absorption corrections were based on Gaussian integrations over a multifaceted crystal model. Empirical absorption corrections were performed using spherical harmonics, implemented in the SCALE3 ABSPACK scaling algorithm. Systematic reflection conditions and statistical tests suggested the space group *P2*_*1*_/*n*, which was confirmed by SHELXT.^42^ Hydrogen atom positions were calculated and refined using a riding model. All non-hydrogen atoms were refined anisotropically. The absence of additional symmetry and voids was confirmed using PLATON (ADDSYM).^43^ The structure was refined (full matrix least-squares refinement on *F*^*2*^) to convergence.^43,44^

## Supporting Information Available

Experimental section; tables; variable-temperature NMR spectra; mass spectrometric analysis of **PtC_4_D**; and NMR spectra of new complexes (PDF)
