# Supplementary material for: Reactions of Platinum Terminal Polyynyl Complexes trans-(C6F5)(p-tol3P)2Pt(C≡C)nH (n = 2–4) and n-BuLi, Generation of Functional Equivalents of Pt(C≡C)nLi Species, and Derivatization with Organic and Inorganic Electrophiles
Source: Organometallics. 2024 Apr 24;43(9):1041–50. doi: 10.1021/acs.organomet.4c00098 (PMC11094795; doi:10.1021/acs.organomet.4c00098)
Supplement: Supplementary file 1 — om4c00098_si_001.pdf [file om4c00098_si_001.pdf]

SUPPORTING INFORMATION FOR

Reactions of Platinum Terminal Polyynyl Complexes *trans*-(C_6F_5)(*p*-tol$_3$P)$_2$Pt(C≡C)$_n$H (*n* = 2-4) and *n*-BuLi; Generation of Functional Equivalents of Pt(C≡C)$_n$Li Species and Derivatization with Organic and Inorganic Electrophiles

Sourajit Dey Baksi[a], Joshua O. Aggrey[b], Nattamai Bhuvanesh[a], and John A. Gladysz[a*]

[a]Department of Chemistry, Texas A&M University, PO Box 30012, College Station, Texas 77842-3012, USA

[b]Department of Chemistry, East Tennessee State University, 1276 Gilbreath Drive, Johnson City, Tennessee, 37614, USA

submitted to *Organometallics*

TABLE OF CONTENTS

	page number
EXPERIMENTAL SECTION (continued)	s2
REFERENCES	s9
TABLES	s10
VARIABLE TEMPERATURE NMR SPECTRA	s14
MASS SPECTROMETRIC ANALYSIS OF **PtC$_4$D**	s15
NMR SPECTRA OF NEW COMPLEXES	s17

■ EXPERIMENTAL SECTION (continued)

General data

Reactions were conducted under dry inert atmospheres using conventional Schlenk techniques, but workups were carried out in air. Chemicals were treated as follows: THF, hexanes, and CH_2Cl_2, passed through a Glass Contour solvent purification system; benzene, MeOH (2 × ACS grade), Me_3SiCl (98+%/Alfa Aesar or 98%/Acros), MeI (Alfa Aesar), *n*-BuLi (2.5 M in hexanes, Sigma-Aldrich), $PhCH_2Br$ (98%/Sigma-Aldrich), $W(CO)_6$ (97%/Sigma-Aldrich), $Me_3O^+ BF_4^-$ (>95%/TCI Chemicals) *n*-$Bu_4N^+ F^-$ (1.0 M in THF, 5 wt% water, Acros), $AgClO_4$ (97%/Sigma-Aldrich), $NaBH_4$ (98%/TCI Chemicals), $CDCl_3$, CD_2Cl_2, C_6D_6, D_2O (4 × Cambridge Isotope Laboratories), $MgSO_4$ (Fisher Chemical), silica gel (Acros, Fluoroflash or 60M Macherey-Nagel), alumina (neutral, Brockmann I, 40-300 μm, Thermo Scientific Chemicals), and Florisil® (Fluka Analytical), used as received.

NMR spectra were obtained on standard 500 MHz spectrometers and referenced as follows (δ/ppm): ^1H, residual $CHCl_3$ (7.24), $CHDCl_2$ (5.32) or C_6D_5H (7.16); $^{13}C\{^1H\}$, internal $CDCl_3$ (77.00), CD_2Cl_2 (54.00) or C_6D_6 (128.06); $^{19}F\{^1H\}$, external C_6F_6 (–164.90); $^{31}P\{^1H\}$, external H_3PO_4 (0.00). IR spectra were recorded on a Shimadzu IRAffinity-1 spectrometer with a Pike MIRacle ATR system (diamond crystal). UV-visible spectra were recorded on a Shimadzu UV-1800 spectrometer. Mass spectra were recorded using Bruker microFlex (MALDI-TOF-MS) or Thermo Scientific Q Exactive Focus (ESI and APCI) instruments. Thin-layer chromatography (TLC) was carried out on EMD Silica Gel 60 F_{254} aluminum plates and visualized using 254 or 365 nm lamps. Microanalyses were conducted by Atlantic Microlab.

Alternative routes to platinum complexes

***trans*-(C_6F_5)(*p*-$tol_3P)_2Pt(C{\equiv}C)_3H$ (PtC_6H).**[s1] modified workup. A Schlenk flask was charged with **PtC_6SiEt_3** (0.350 g, 0.296 mmol)[s1] and CH_2Cl_2 (30 mL). Then *n*-$Bu_4N^+ F^-$ (1.0 M in THF/5 wt% H_2O, 0.100 mL, 0.100 mmol) was added with stirring. The disappearance of **PtC_6-$SiEt_3$** was monitored by alumina TLC (1:3 v/v CH_2Cl_2/hexanes). After 15 min, cold water (40 mL) was added. The CH_2Cl_2 phase was separated, and the aqueous phase was extracted with cold

CH_2Cl_2 (2 × 50 mL). The combined CH_2Cl_2 phases were dried ($MgSO_4$). The solution was concentrated to ca. 3 mL by rotary evaporation at 0 °C and chromatographed on an ice-jacketed alumina column (3 × 25 cm, packed in cold hexane, and eluted with cold 1:4 v/v CH_2Cl_2/hexanes), with the collection flasks also iced. The solvent was removed from the product-containing fractions by rotary evaporation at 0 °C. The off-white powder was washed with cold pentane to give **PtC_6H** (0.210 g, 0.197 mmol, 67%), which was stable at room temperature for 1 d and further used as soon as possible. The ^1H and $^{31}P\{^1H\}$ NMR, IR, and MS data agreed with those reported previously,[s1] and the following are new to this study.

NMR (δ/ppm, $CDCl_3$): $^{13}C\{^1H\}$ (126 MHz, cryoprobe)[s2] 147.1-144.8 (dm, $^1J_{CF}$ = 226 Hz, *o* to Pt), 139.7 (s, *p* to P), 138.0-137.5 (dm, *p* to Pt), 136.6-135.0 (dm, *m* to Pt), 133.8 (virtual t, $^2J_{CP}$ = 12.6 Hz,[s3] *o* to P), 127.9 (virtual t, $^3J_{CP}$ = 11.3 Hz,[s3] *m* to P), 125.8 (virtual t, $^1J_{CP}$ = 30.5 Hz,[s3] *i* to P), 103.3 (br s, Pt<u>*C*</u>≡C),[s4] 95.1 (s, PtC≡<u>*C*</u>), 70.6, 65.2, 63.9, 55.4 (4 s, PtC≡C<u>*C*</u>≡<u>*CC*</u>≡<u>*C*</u>), 21.5 (s, <u>*C*</u>H_3, *p* to P).

***trans*-(C_6F_5)(*p*-tol$_3$P)$_2$Pt(C≡C)$_4$H (PtC_8H).**[s6] *triethylsilyl vs. trimethylsilyl precursor.* A Schlenk flask was charged with **PtC_8SiEt_3** (0.120 g, 0.102 mmol) and CH_2Cl_2 (20 mL).[s1] Then *n*-Bu_4N^+ F^- (1.0 M in THF/5 wt% H_2O, 0.036 mL, 0.036 mmol) was added with stirring. The disappearance of **PtC_8SiEt_3** was monitored by alumina TLC (1:3 v/v CH_2Cl_2/hexanes). After 15 min, cold water (30 mL) was added. The CH_2Cl_2 phase was separated, and the aqueous phase extracted with cold CH_2Cl_2 (2 × 40 mL). The combined CH_2Cl_2 phases were dried ($MgSO_4$). The solution was concentrated to ca. 3 mL by rotary evaporation at 0 °C. The residue was chromatographed on an ice-jacketed alumina column (3 × 25 cm, packed in cold hexane, and eluted with cold 1:4 v/v CH_2Cl_2/hexanes), with the collection flasks also iced. The solvent was removed from the product-containing fractions by rotary evaporation at 0 °C. The pale-yellow powder was washed with cold pentane to give **PtC_8H** (0.065 g, 0.061 mmol, 60%), which was stable at room temperature for 1 d and further used as soon as possible. The NMR data agreed with those reported previously.[s6]

***trans*-(C_6F_5)(*p*-tol$_3$P)$_2$PtH (PtH).** *isolation as a byproduct in the syntheses of* **PtC_xMe** *(x*

= 4, 6, 8). A Schlenk flask was charged with **PtC_4H** (0.117 g, 0.115 mmol)[s1] and THF (30 mL) and cooled to –45 °C (dry ice/acetonitrile). Then *n*-BuLi (0.068 mL, 2.5 M in hexanes, 0.17 mmol) was added dropwise with stirring. The yellow solution turned orange. After 30 min, the –45 °C bath was replaced by an ice bath. After 15 min, MeI (0.016 mL, 0.26 mmol) was added. After 16 h, the volatiles were removed by oil-pump vacuum and MeOH (10 mL) was added. The creamy white solid was collected by filtration and chromatographed on a neutral alumina column (3 × 30 cm, packed in hexanes, eluted with a 0:7 → 1:7 v/v CH_2Cl_2/hexanes gradient). The first fraction was collected and the solvent was removed by oil-pump vacuum to give **PtC_4Me** (see preparation in main text). The column was further eluted with a 1:7 → 1:2 v/v CH_2Cl_2/hexanes gradient. The solvent was removed from these fractions by oil-pump vacuum to give a clear oil that solidified overnight at 4 °C. This was recrystallized from CH_2Cl_2/methanol to give **PtH** (0.012 g, 0.013 mmol, 11%) as off-white flakes. The NMR spectra agreed with those reported in the main text; additional data: $^{31}P\{^1H\}$ (THF, 202 MHz) 27.6 (s, $^1J_{PPt}$ = 2977 Hz).[s5]

Additional NMR data for previously reported compounds (new solvents, decoupling modes)

***trans*-$(C_6F_5)(p\text{-tol}_3P)_2Pt(C{\equiv}C)_2H$ (PtC_4H).**[s1] NMR (δ/ppm, $CDCl_3$): ^{13}C (126 MHz)[s2] 145.8 (dm, $^1J_{CF}$ = 230 Hz, *o* to Pt), 140.8 (s, *p* to P), 136.2 (dm, *p* and *m* to Pt), 134.4 (d, $^1J_{CH}$ = 153 Hz, *o* to P), 130.1 (d, $^1J_{CH}$ = 161 Hz, *m* to P), 127.4 (m, overlapping with upfield line of d, *i* to P), 98.0 (s, Pt$\underline{C}$≡C), 95.1 (s, PtC≡$\underline{C}$), 72.7 (d, $^2J_{CH}$ = 50 Hz, PtC≡C$\underline{C}$≡C), 60.3 (d, $^1J_{CH}$ = 253 Hz, PtC≡CC≡$\underline{C}$), 21.4 (q, $^1J_{CH}$ = 382 Hz, $\underline{C}H_3$, *p* to P); $^{13}C\{^1H\}$ (126 MHz, cryoprobe)[s2] 145.8 (dm, $^1J_{CF}$ = 224 Hz, *o* to Pt), 140.7 (s, *p* to P), 136.8 (m, *p* to Pt), 136.3 (m, *m* to Pt), 134.3 (virtual t, $^2J_{CP}$ = 12 Hz,[s3] *o* to P), 128.6 (virtual t, $^3J_{CP}$ = 10 Hz,[s3] *m* to P), 127.4 (virtual t, $^1J_{CP}$ = 31 Hz,[s3] *i* to P), 97.8 (br s, Pt$\underline{C}$≡C),[s4] 94.9 (s, PtC≡$\underline{C}$), 72.5 (s, PtC≡C$\underline{C}$≡C), 59.6 (s, PtC≡CC≡$\underline{C}$), 21.3 (s, $\underline{C}H_3$, *p* to P). $^{31}P\{^1H\}$ (THF, 202 MHz, RT/–45 °C) 18.0/18.3 (s, $^1J_{PPt}$ = 2663/2658 Hz).[s5]

$^{19}F\{^1H\}$ NMR data (δ/ppm, 470 MHz) for complexes in this study

PtC_6H ($CDCl_3$): −117.6 (m, $^3J_{FPt}$ = 291 Hz,[s5] 2F, *o* to Pt), −165.4 (m, 2F, *m* to Pt), −166.0 (t, $^3J_{FF}$ = 21.1 Hz, 1F, *p* to Pt).

PtC_4Me ($CDCl_3$): −117.6 (m, $^3J_{FPt}$ = 291 Hz,[s5] 2F, *o* to Pt), −165.6 (m, 2F, *m* to Pt),

−166.4 (t, $^3J_{FF}$ = 21 Hz, 1F, *p* to Pt).

PtC_4SiMe_3 (CD_2Cl_2): −117.6 (m, $^3J_{FPt}$ = 291 Hz,[s5] 2F, *o* to Pt), −165.5 (m, 2F, *m* to Pt), −166.0 (t, $^3J_{FF}$ = 21 Hz, 1F, *p* to Pt).

PtC_4D ($CDCl_3$): −117.7 (m, $^3J_{FPt}$ = 291 Hz,[s5] 2F, *o* to Pt), −165.5 (m, 2F, *m* to Pt), −166.0 (t, $^3J_{FF}$ = 21 Hz, 1F, *p* to Pt).

PtC_4Bn ($CDCl_3$): −117.6 (m, $^3J_{FPt}$ = 291 Hz,[s5] 2F, *o* to Pt), −165.4 (m, 2F, *m* to Pt), −166.1 (t, $^3J_{FF}$ = 21.3 Hz, 1F, *p* to Pt).

$PtC_4C(OMe){=}W$ (C_6D_6): −117.2 (m, $^3J_{FPt}$ = 288 Hz,[s5] 2F, *o* to Pt), −164.5 (m, 2F, *m* to Pt), −165.2 (t, $^3J_{FF}$ = 20 Hz, 1F, *p* to Pt).

PtC_6Me ($CDCl_3$): −116.2 (m, $^3J_{FPt}$ = 291 Hz,[s5] 2F, *o* to Pt), −163.4 (m, 2F, *m* to Pt), −165.4 (t, $^3J_{FF}$ = 21 Hz, 1F, *p* to Pt).

PtC_8Me ($CDCl_3$): −116.2 (m, $^3J_{FPt}$ = 291 Hz,[s5] 2F, *o* to Pt), −163.4 (m, 2F, *m* to Pt), −165.5 (t, $^3J_{FF}$ = 21 Hz, 1F, *p* to Pt).

PtH ($CDCl_3$): −116.4 (m, 2F, *o* to Pt), −165.9 (m, 2F, *m* to Pt), −166.9 (t, $^3J_{FF}$ = 38 Hz, 1F, *p* to Pt).

Crystallography

A. A CH_2Cl_2 solution of **PtC_4Me** was layered with hexanes and kept at 4 °C. After 3 d, colorless blocks were collected. Cell parameters were determined from 60 data frames taken at widths of 0.5° and refined with 111645 reflections using CrysAlisPro.[s7] Numerical absorption corrections were based on Gaussian integrations over a multifaceted crystal model. Empirical absorption corrections were performed using spherical harmonics, implemented in SCALE3 ABSPACK scaling algorithm. Systematic reflection conditions and statistical tests suggested the space group $P2_1/n$, which was confirmed by SHELXT.[s8] Hydrogen atom positions were calculated and refined using a riding model. All non-hydrogen atoms were refined anisotropically. The absence of additional symmetry and voids was confirmed using PLATON (ADDSYM).[s9] The structure was refined (full matrix least squares refinement on F^2) to convergence.[s9,s10]

B. A CH_2Cl_2 solution of **PtC_6Me** was layered with hexanes and kept at 4 °C. After several d, yellow blocks were collected. Cell parameters were determined from 60 data frames taken at widths of 0.5° and refined with 237473 reflections using CrysAlisPro.[s7] Numerical absorption

corrections were based on Gaussian integrations over a multifaceted crystal model. Empirical absorption corrections were performed using spherical harmonics, implemented in SCALE3 ABSPACK scaling algorithm. Systematic reflection conditions and statistical tests suggested the space group *R*-3, which was confirmed by SHELXT.[s8] Some CH_2Cl_2 was located and modeled with appropriate restraints. The occupancy was initially refined and then fixed as 0.08 for the final least squares refinement cycles. Hydrogen atom positions were calculated and refined using a riding model. All non-hydrogen atoms were refined anisotropically. The absence of additional symmetry and voids was confirmed using PLATON (ADDSYM).[s9] The structure was refined (full matrix least squares refinement on F^2) to convergence.[s9,s10]

C. A CH_2Cl_2 solution of **PtC$_8$Me** was layered with hexanes and kept at 4 °C. After several d, yellow blocks were collected. Cell parameters were determined from 60 data frames taken at widths of 0.5° and refined with 88991 reflections using CrysAlisPro.[s7] Numerical absorption corrections were based on Gaussian integration over a multifaceted crystal model. Empirical absorption corrections were performed using spherical harmonics, implemented in SCALE3 ABSPACK scaling algorithm. Systematic reflection conditions and statistical tests suggested the space group *I*2/*a*, which was confirmed by SHELXT.[s8] Hydrogen atom positions were calculated and refined using a riding model. All non-hydrogen atoms were refined anisotropically. The absence of additional symmetry and voids was confirmed using PLATON (ADDSYM).[s9] The structure was refined (full matrix least squares refinement on F^2) to convergence.[s9,s10]

D. A benzene solution of **PtC$_4$C(OMe)=W** was layered with hexanes and kept at −35 °C. After 5 d, brown blocks were collected. Cell parameters were determined from 45 data frames taken at widths of 1° and refined with 10396 reflections using APEX3.[s11] Data were corrected for Lorentz and polarization factors, and (using SADABS)[s8] crystal decay and absorption effects. Systematic reflection conditions and statistical tests suggested the space group *P*-1, which was confirmed by SHELXT.[s8] Hydrogen atom positions were calculated and refined using a riding model. All non-hydrogen atoms were refined anisotropically. CHECKCIF indicated the presence of a solvent accessible void of 33 Å^3. However, the absence of additional symmetry and voids was

confirmed using PLATON (ADDSYM).[s9] The structure was refined (weighted least squares refinement on F^2) to convergence.[s9,s10]

E. crystal 1. A CH_2Cl_2 solution of **PtC_4Si** was layered with hexanes and kept at − 35 °C. After several d, colorless plates were collected. Cell parameters were determined from 60 data frames taken at widths of 0.5° and refined with 9447 reflections using CrysAlisPro.[s7] Numerical absorption corrections were based on Gaussian integration over a multifaceted crystal model. Empirical absorption corrections were performed using spherical harmonics, implemented in SCALE-3 ABSPACK scaling algorithm. Systematic reflection conditions and statistical tests suggested the space group $P2_1/c$, which was confirmed by SHELXT.[s8] Hydrogen atom positions were calculated and refined using a riding model. All non-hydrogen atoms were refined anisotropically. The absence of additional symmetry and voids was confirmed using PLATON (ADDSYM).[s9] Elongated thermal ellipsoids were apparent for C28 to C34. This suggested disorder, which was successfully modeled between two positions with an occupancy ratio of 64:36. Appropriate restraints were added to keep the bond distances, angles, and thermal ellipsoids meaningful. The structure was refined (full matrix least squares refinement on F^2) to convergence.[s9,s10]

F. crystal 2. A CH_2Cl_2 solution of **PtC_4Si** was layered with hexanes and kept at 4 °C. After 3 d, colorless plates were collected. Cell parameters were determined from 45 data frames taken at widths of 1° and refined with 18990 reflections using APEX3.[s11] Numerical absorption corrections were based on Gaussian integration over a multifaceted crystal model. Data were corrected for Lorentz and polarization factors, and (using SADABS)[s8] crystal decay and absorption effects. Systematic reflection conditions and statistical tests suggested the space group $P2_1/c$, which was confirmed by SHELXT.[s8] Hydrogen atom positions were calculated and refined using a riding model. All non-hydrogen atoms were refined anisotropically. The absence of additional symmetry and voids was confirmed using PLATON (ADDSYM).[s9] The structure was refined (full matrix least squares refinement on F^2) to convergence.[s9,s10]

G. A CH_2Cl_2 solution of **PtC_4Si** was layered with toluene and kept at − 35 °C. After 4 d, yellow blocks were collected. Cell parameters were determined from 60 data frames taken at

widths of 0.5° and refined with 96792 reflections using CrysAlisPro.[s7] Numerical absorption corrections were based on Gaussian integration over a multifaceted crystal model. Empirical absorption corrections were performed using spherical harmonics, implemented in SCALE3 ABSPACK scaling algorithm. Systematic reflection conditions and statistical tests suggested the space group *P*-1, which was confirmed by SHELXT.[s8] Hydrogen atom positions were calculated and refined using a riding model. All non-hydrogen atoms were refined anisotropically. The absence of additional symmetry and voids was confirmed using PLATON (ADDSYM).[s9] Elongated thermal ellipsoids on Si1 and C5 to C7 suggested disorder which was modeled between two positions with an occupancy ratio of 54:46. Toluene was found in three locations. Elongated ellipsoids and electron density peaks near two of them suggested disorder, which was modeled between two positions. Their occupancies were initially refined independently, and then fixed for the final least squares refinement cycles for convergence. Q peaks near one of the disordered toluene molecules suggested additional minor disorder, but no efforts were made to model this. Appropriate restraints were added to keep the bond distances, angles, and thermal ellipsoids meaningful. The structure was refined (full matrix least squares refinement on F^2) to convergence.[s9,s10]

H. A CH_2Cl_2 solution of **PtH** was layered with hexanes and kept at −35 °C. After 7 d, colorless plates were collected. Cell parameters were determined from 60 data frames taken at widths of 0.5° and refined with 88991 reflections using CrysAlisPro.[s7] Numerical absorption corrections were based on Gaussian integration over a multifaceted crystal model. Empirical absorption corrections were performed using spherical harmonics, implemented in SCALE3 ABSPACK scaling algorithm. Systematic reflection conditions and statistical tests suggested the space group *R*-3c, which was confirmed by SHELXT.[s8] Residual electron density peaks indicated the presence of (1) a hydrogen atom on platinum, which was set riding, and (2) partially occupied and disordered solvent molecules. Efforts to model the solvent were unsuccessful. For the final least square refinement cycles, a solvent mask was calculated indicating 11 electrons. This is consistent with 0.26 molecules of CH_2Cl_2 per asymmetric unit. The structure was refined (full matrix least squares refinement on F^2) to convergence.[s9,s10] Each molecule of **PtH** exhibited a C_2 symmetry axis.

■ REFERENCES

(s1) Mohr, W.; Stahl, J.; Hampel, F.; Gladysz, J. A. Synthesis, Structure, and Reactivity of sp Carbon Chains with Bis(phosphine)Pentafluorophenylplatinum Endgroups: Butadiynediyl (C_4) through Hexadecaoctaynediyl (C_{16}) Bridges, and Beyond. *Chem. Eur. J.* **2003**, *9*, 3324-3340.

(s2) The *ipso* C_6F_5 signal was not observed.

(s3) Hersh W. H. False AA'X Spin-Spin Coupling Systems in ^{13}C NMR: Examples Involving Phosphorus and a 20-Year-Old Mystery in Off-Resonance Decoupling, *J. Chem. Educ*. **1997**, *74*, 1485-1488. The $^{n}J_{CP}$ values represent the distance between adjacent peaks in the apparent triplet, with *n* the number of intervening bonds in a first-order analysis.

(s4) The expected first order couplings were not resolved due to poor signal/noise ratio.

(s5) This coupling represents a satellite (d; ^{195}Pt = 33.8%) and is not reflected in the peak multiplicity given.

(s6) Amini, H.; Baranová, Z.; Weisbach, N.; Gauthier, S.; Bhuvanesh, N.; Reibenspies, J. H.; Gladysz, J. A. Syntheses, Structures, and Spectroscopic Properties of 1,10-Phenanthroline-Based Macrocycles Threaded by PtC_8Pt, $PtC_{12}Pt$, and $PtC_{16}Pt$ Axles: Metal-Capped Rotaxanes as Insulated Molecular Wires. *Chem. Eur. J.* **2019**, *25*, 15896-15914.

(s7) *CrysAlisPRO* Software System, Rigaku Oxford Diffraction/Agilent Technologies UK Ltd, Yarnton, England (2023).

(s8) (a) Sheldrick, G. M. *SADABS, Program for Absorption Correction for Data from Area Detector Frames*. Bruker AXS Inc., Madison, WI 53711-5373 USA. (b) Sheldrick, G. M. SHELXT – Integrated space-group and crystal structure determination. *Acta Cryst*. **2015**, *A71*, 3-8. (c) Sheldrick, G. M. Crystal structure refinement with *SHELXL*. *Acta Cryst*. **2015**, *C71*, 3-8.

(s9) Spek, A. L. Single-crystal structure validation with the program *PLATON*. *J. Appl. Cryst.* **2003**, *36*, 7-13.

(s10) Dolomanov, O. V, Bourhis, L. J., Gildea, R. J., Howard, J. A. K., Puschmann, H. OLEX2: A Complete Structure Solution, Refinement and Analysis Program. *J. Appl. Cryst*. **2009**, *42*, 339-341.

(s11) *APEX3, Program for Data Collection on Area Detectors.* Bruker AXS Inc., Madison, WI 53711-5373 USA.

Table s1. Crystallographic data for complexes with Pt(C≡C)$_n$Me and PtH linkages.

	**PtC$_4$Me**	**PtC$_6$Me**·$(CH_2Cl_2)_{0.08}$	**PtC$_8$Me**	**PtH**·$(CH_2Cl_2)_{0.52}$
empirical formula	$C_{53}H_{45}F_5P_2Pt$	$C_{55.08}H_{45.16}Cl_{0.16}F_5P_2Pt$	$C_{57}H_{45}F_5P_2Pt$	$C_{48.52}H_{44.2}Cl_{1.2}F_5P_2Pt$
formula weight	1033.92	1064.73	1081.96	1016.01
temperature [K]	100.0	100.0	100.0	100.0
diffractometer	XtaLAB Synergy	XtaLAB Synergy	XtaLAB Synergy	XtaLAB Synergy
wavelength [Å]	1.54178	0.71073	1.54184	1.54184
crystal system	monoclinic	trigonal	monoclinic	trigonal
space group	$P2_1/n$	*R*-3	*I*2/*a*	*R*-3*c*
unit cell dimensions				
a [Å]	10.79695(4)	23.89780(10)	25.52930(10)	24.38440(10)
b [Å]	11.83200(5)	23.89780(10)	12.24910(10)	24.38440(10)
c [Å]	36.48946(15)	43.1239(2)	32.6667(2)	40.35190(10)
α [°]	90	90	90	90
β [°]	95.7521(4)	90	106.7930(10)	90
γ [°]	90	120	90	120
volume [Å^3]	4638.04(3)	21328.70(2)	9779.60(12)	20778.71(18)
Z/Z′	4/1	18/1	8/1	18/0.5
ρ_{calc} [Mg/m^3]	1.481	1.492	1.470	1.462
μ [nm^{-1}]	6.765	3.093	6.446	7.323
F(000)	2064	9564	4320	9105
crystal size [mm]	0.09 × 0.06 × 0.04	0.28 × 0.14 × 0.06	0.19 × 0.12 × 0.03	0.32 × 0.23 × 0.08
Θ range [°]	2.434 to 80.051	2.566 to 37.903	2.826 to 80.068	3.029 to 80.026
index ranges	$-9 \le h \le 13$	$-40 \le h \le 41$	$-32 \le h \le 32$	$-30 \le h \le 31$
	$-14 \le k \le 15$	$-41 \le k \le 40$	$-15 \le k \le 12$	$-31 \le k \le 31$
	$-46 \le l \le 46$	$-73 \le l \le 74$	$-40 \le l \le 41$	$-51 \le l \le 49$
reflections collected	108792	237250	88991	197583
independent reflections	10010 [R(int) = 0.0301]	25197 [R(int) = 0.0369]	10419 [R(int) = 0.0299]	5042 [R(int) = 0.0432]
data/restraints/parameters	9684/0/557	23447/3/594	9757/0/593	4957/0/258
goodness-of-fit on F^2	1.081	1.063	1.093	1.075
final R indices [I>2σ(I)]	$R1 = 0.0197$, $wR^2 = 0.0508$	$R1 = 0.0271$, $wR^2 = 0.0534$	$R1 = 0.0264$, $wR^2 = 0.0655$	$R1 = 0.0253$, $wR^2 = 0.0619$
R indices (all data)	$R1 = 0.0204$, $wR^2 = 0.0504$	$R1 = 0.0314$, $wR^2 = 0.0544$	$R1 = 0.0281$, $wR^2 = 0.0668$	$R1 = 0.0253$, $wR^2 = 0.0621$
largest diff. peak/hole [eÅ^{-3}]	0.356 and –0.751	1.664 and –0.503	1.646 and –1.513	0.778 and –1.025

Table s2. Crystallographic data for complexes derived from **PtC_4H** and inorganic electrophiles.

	**PtC_5WOMe**	**PtC_4Si** (crystal 1)	**PtC_4Si** (crystal 2)	**PtC_4Si**·$(C_7H_8)_{2.7}$
empirical formula	$C_{59}H_{45}F_5O_6P_2PtW$	$C_{55}H_{51}F_5P_2PtSi$	$C_{55}H_{51}F_5P_2PtSi$	$C_{73.9}H_{72.6}F_5P_2PtSi$
formula weight	1385.83	1092.07	1092.07	1340.84
temperature [K]	110.0	100.0	110.0	110.0
diffractometer	Bruker Venture	XtaLAB Synergy	Bruker Quest	XtaLAB Synergy
wavelength [Å]	1.54178	1.54184	0.71073	0.56087
crystal system	triclinic	monoclinic	monoclinic	triclinic
space group	*P*-1	$P2_1/c$	$P2_1/c$	*P*-1
unit cell dimensions				
a [Å]	11.7302(3)	13.12536(5)	13.1452(6)	13.8418(2)
b [Å]	16.0387(4)	13.04271(6)	13.0485(6)	16.4378(2)
c [Å]	16.4636(4)	29.09194(12)	29.0842(14)	17.1743(2)
α [°]	64.5180(10)	90	90	74.2800(10)
β [°]	86.7780(10)	92.8404(4)	92.828(2)	67.8540(10)
γ [°]	77.2450 (10)	90	90	68.9490(10)
volume [$Å^3$]	2724.45(12)	4974.14(4)	4982.6(4)	3336.04(8)
Z/Z′	2/1	4/1	4/1	2/1
ρ_{calc} [Mg/m^3]	1.689	1.458	1.456	1.335
μ [nm^{-1}]	9.698	6.562	2.958	1.204
F(000)	1348	2192	2192	1366
crystal size [mm]	0.24 × 0.12 × 0.03	0.26 × 0.24 × 0.02	0.26 × 0.24 × 0.02	0.26 × 0.07 × 0.05
Θ range [°]	2.976 to 70.870	3.042 to 70.073	2.568 to 33.178	2.152 to 25.559
index ranges	$-14 \leq h \leq 12$	$-11 \leq h \leq 15$	$-20 \leq h \leq 20$	$-21 \leq h \leq 21$
	$-19 \leq k \leq 19$	$-15 \leq k \leq 15$	$-20 \leq k \leq 20$	$-25 \leq k \leq 25$
	$-20 \leq l \leq 20$	$-35 \leq l \leq 35$	$-43 \leq l \leq 44$	$-26 \leq l \leq 24$
reflections collected	51721	131832	105942	96792
independent reflections	10396 [R(int) = 0.0424]	9447 [R(int) = 0.0361]	18990 [R(int) = 0.0760]	22182 [R(int) = 0.0370]
data/restraints/parameters	9733/0/673	9234/345/651	13576/0/586	13576/911/887
goodness-of-fit on F^2	1.028	1.190	1.036	1.050
final R indices [I>2σ(I)]	$R1 = 0.0349$, $wR^2 = 0.0931$	$R1 = 0.0252$, $wR^2 = 0.0579$	$R1 = 0.0439$, $wR^2 = 0.0829$	$R1 = 0.0290$, $wR^2 = 0.0694$
R indices (all data)	$R1 = 0.0370$, $wR^2 = 0.0944$	$R1 = 0.0260$, $wR^2 = 0.0583$	$R1 = 0.0750$, $wR^2 = 0.0923$	$R1 = 0.0368$, $wR^2 = 0.0727$
largest diff. peak/hole [$eÅ^{-3}$]	1.586 and –2.277	0.672 and –0.510	1.521 and –1.512	2.467 and –0.734

Table s3. Key crystallographic distances [Å] and angles [°][a]

	**PtC_4Me**	**PtC_6Me**·$(CH_2Cl_2)_{0.08}$	**PtC_8Me**	**$PtC_4C(OMe)$=W**	**PtC_4Si** (crystal 1)	**PtC_4Si** (crystal 2)	**PtC_4Si·**$(C_7H_8)_{2.7}$
Pt-C1	2.009(2)	1.993(18)	1.983(3)	2.003(5)	2.010(3)	2.004(3)	1.999(17)
C1≡C2	1.209(3)	1.225(3)	1.210(4)	1.205(7)	1.205(4)	1.207(5)	1.217(2)
C2-C3	1.382(3)	1.365(3)	1.369(4)	1.366(7)	1.384(4)	1.380(5)	1.374(3)
C3≡C4	1.203(3)	1.206(3)	1.212(4)	1.214(8)	1.209(4)	1.217(5)	1.214(3)
C4-C5	–	1.371(3)	1.356(4)	1.415(7)	–	–	–
C5≡C6	–	1.200(3)	1.215(4)	–	–	–	–
C6-C7	–	–	1.367(4)	–	–	–	–
C7≡C8	–	–	1.197(4)	–	–	–	–
Cω[b]-C or Si	1.468(3)	1.457(3)	1.455(4)	–	1.839(3)	1.832(4)	1.830(7)[c]
Pt-P1	2.298(5)	2.308(4)	2.309(6)	2.315(11)	2.305(7)	2.301(8)	2.310(4)
Pt-P2	2.303(5)	2.305(4)[d]	2.307(6)	2.304(11)	2.299(6)	2.307(9)	2.302(4)
C5[e]=W1	–	–	–	2.147(5)	–	–	–
C5[e]-O1	–	–	–	1.325(6)	–	–	–
C6[e]-O1	–	–	–	1.467(7)	–	–	–
C7[e]-W1	–	–	–	2.041(6)	–	–	–
C8[e]-W1	–	–	–	2.040(7)	–	–	–
C9[e]-W1	–	–	–	2.044(6)	–	–	–
C10[e]-W1	–	–	–	2.046(5)	–	–	–
C11[e]-W1	–	–	–	2.051(7)	–	–	–
C7-O2	–	–	–	1.147(8)	–	–	–
C8-O3	–	–	–	1.144(8)	–	–	–
C9-O4	–	–	–	1.134(8)	–	–	–
C10-O5	–	–	–	1.132(7)	–	–	–
C11-O6	–	–	–	1.125(8)	–	–	–
C_{ipso}[f]-Pt-C1	177.1(8)	178.4(7)	178.6(10)	174.8(18)	179.2(11)	179.1(13)	178.7(7)
Pt-C1-C2	172.1(2)	177.6(16)	178.4(2)	174.0(4)	178.3(2)	178.0(3)	179.2(15)
C1-C2-C3	175.8(3)	178.2(19)	179.3(3)	176.8(6)	176.9(3)	177.2(4)	178.2(2)
C2-C3-C4	177.4(3)	178.9(2)	179.2(3)	177.9(6)	178.3(4)	178.0(3)	178.1(2)
C3-C4-C5	–	178.5(2)	177.1(3)	170.8(6)	–	–	–
C4-C5-C6	–	178.6(6)	177.6(3)	–	–	–	–

C5-C6-C7	–	–	178.7(3)	–	–	–	–
C6-C7-C8	–	–	178.6(3)	–	–	–	–
C(ω–1)-Cω[b]-C or Si	177.5(3)	179.0(3)	178.5(3)	–	176.3(3)	176.8(4)	171.5(3)[c]
P1-Pt-P2	177.6(16)	176.5(16)[d]	177.6(2)	178.0(4)	177.6(2)	177.6(3)	175.9(16)
C5-O1-CH_3	–	–	–	120.2(4)	–	–	–
C4-C5-O1-CH_3	–	–	–	−1.0(8)	–	–	–
W=C5-O1-CH_3	–	–	–	178.9(5)	–	–	–
π-stacking distances[g]	3.73/3.48	3.98/3.91	3.81/3.56	3.43/3.50	3.69/3.58	3.66/3.59	3.56/4.01
stacking angle[h]	151.5	158.2	158.1	162.6	162.0	162.2	154.5
C_{ipso}-Pt-P-C_{ipso}'[i,j]	−12.4, 11.8	8.8, 7.9	20.5, 13.8	−6.7, 0.6	−7.8, −7.1	−7.1, −3.9	5.2, −8.9

[a]Those for **PtH**·$(CH_2Cl_2)_{0.52}$ are given in the caption of Figure 3. [b]The sp carbon atom connected to the methyl or trimethylsilyl group. [c]This value is for the dominant conformation in the crystal. [d]For this complex, P2 is denoted P5 in the cif file. [e]The carbon atom numbering of **$PtC_4C(OMe)=W$** parallels those of the platinum complexes through C4, but then diverges. [f]The ligating carbon of the C_6F_5 ring. [g]Distances between the centroids of the C_6F_5 and two $C_6H_4CH_3$ rings. [h]The angle of the centroids of the three rings in *g*. [i]The phosphorus-bound carbon atom of the $C_6H_4CH_3$ ring. [j]When the torsion angle is 0°, the C_6F_5 and $C_6H_4CH_3$ groups are positioned directly above/below each other (although some independent tilting remains possible).

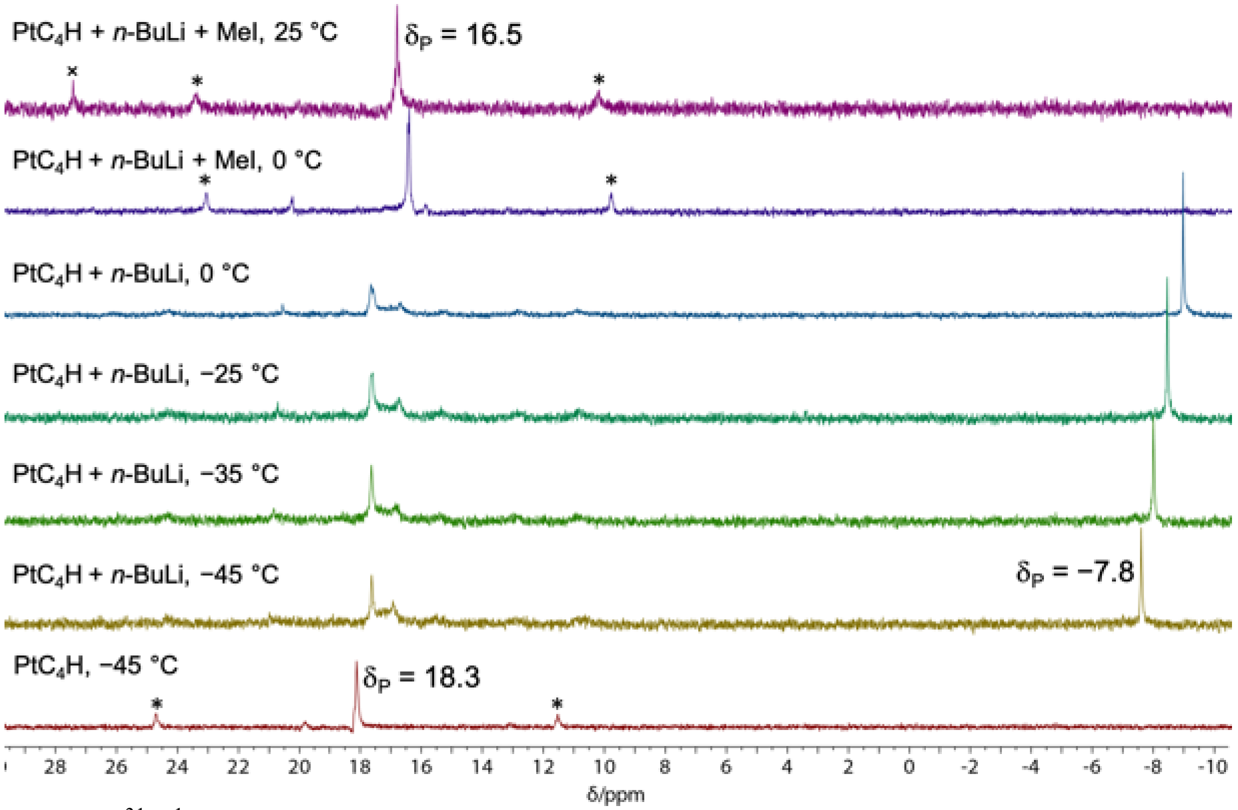

Figure s1. $^{31}P\{^{1}H\}$ NMR monitoring of the deprotonation/methylation sequence for **PtC$_4$H** (THF; Scheme 3). This is the full data set from which Figure 1 was extracted. * denotes a satellite due to ^{195}Pt coupling (33.8% abundance) and × denotes the peak of **PtH** (δ_P = 27.6). No signals were observed outside of the –10 to 30 ppm window.

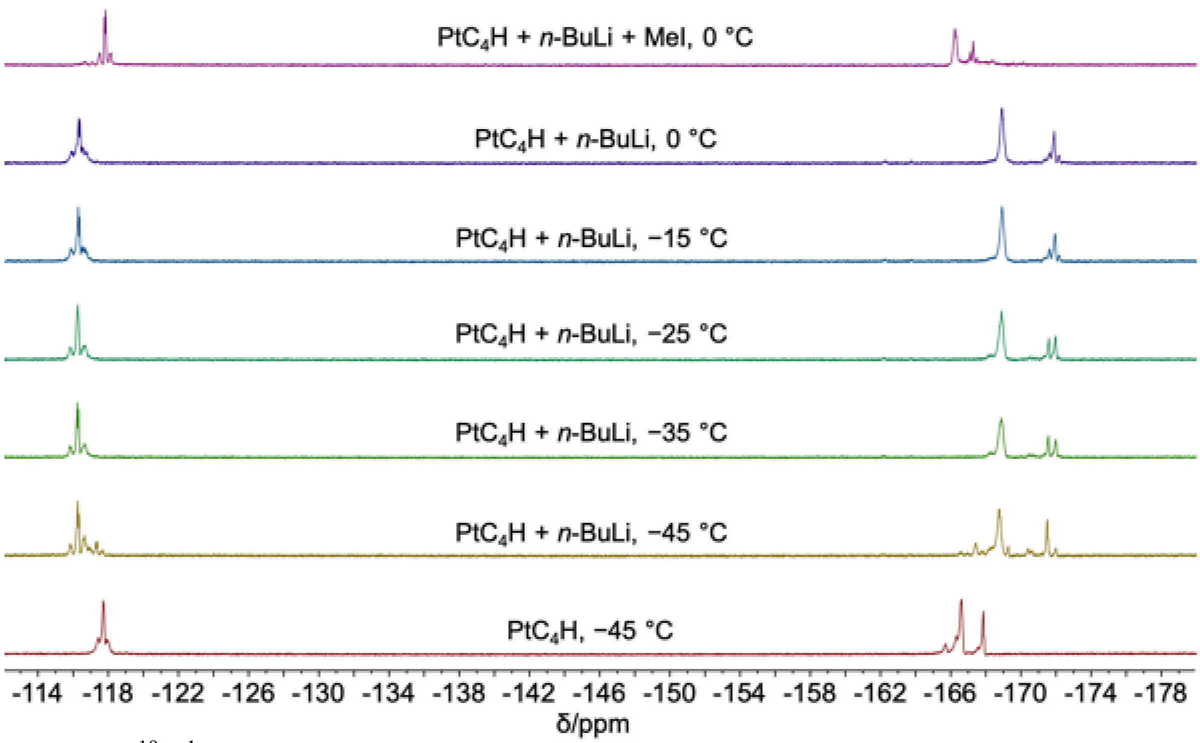

Figure s2. $^{19}F\{^{1}H\}$ NMR monitoring of the deprotonation/methylation sequence of **PtC$_4$H** under conditions analogous to those used in Figure s1. No signals were observed outside of the –178 to –144 ppm window.

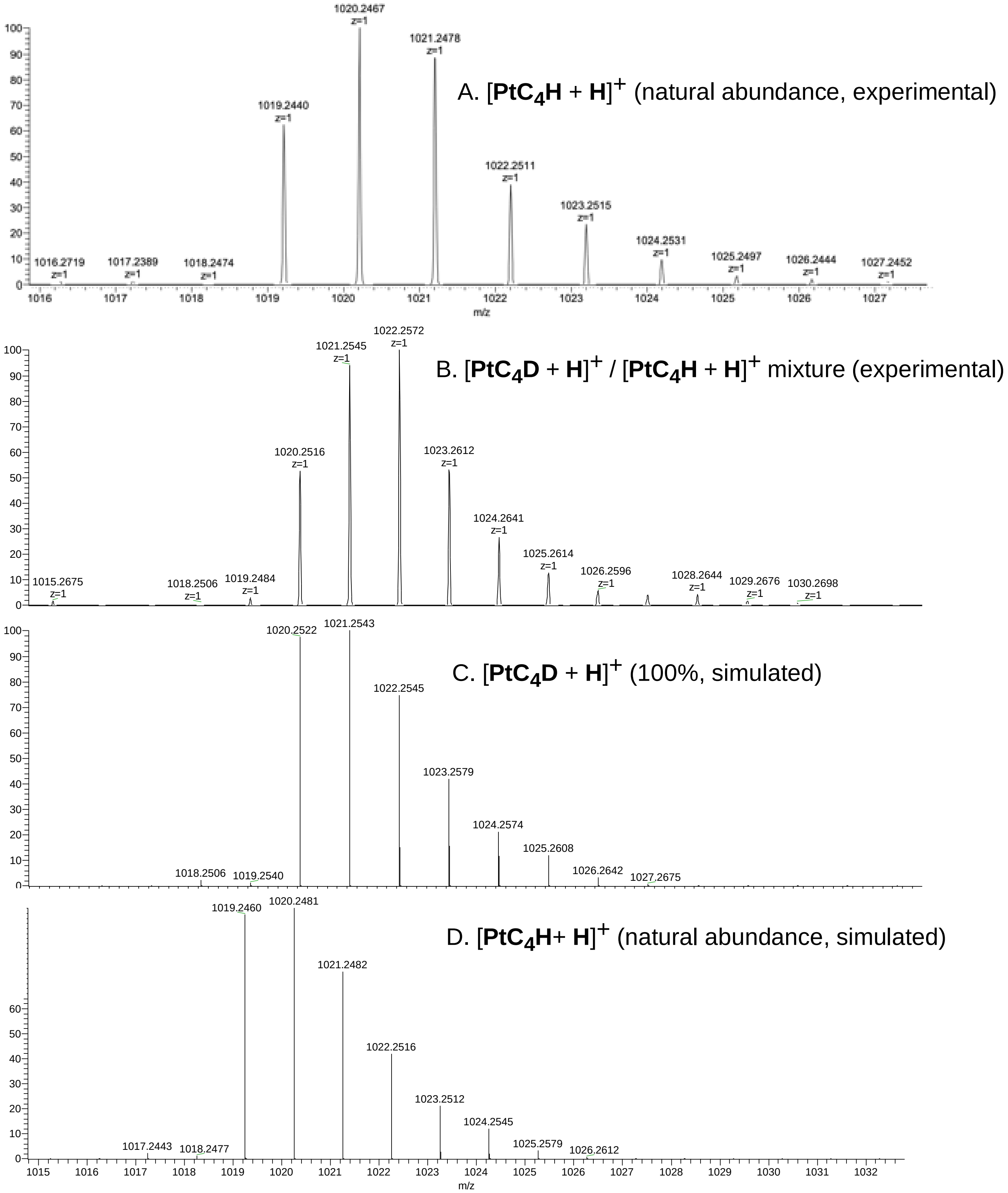

Figure s3. ESI^{+} mass spectra used to analyze the deuterium level in **PtC$_4$D**.

Analysis of deuterium content of isolated PtC_4D (Scheme 3).

Figure s3 shows a mass spectrum (A) of $\mathbf{PtC_4H}$ (natural deuterium abundance) and that of a sample produced from $\mathbf{PtC_4H}$, *n*-BuLi, and D_2O (B), which in the desired limit would consist only of $\mathbf{PtC_4D}$. However, the ^{1}H NMR spectrum shows that it contains some residual $\mathbf{PtC_4H}$. Thus, the mass spectra of $\mathbf{PtC_4D}$ (100% C-deuterated) and $\mathbf{PtC_4H}$ (natural abundance) were simulated as shown in C and D. In every spectrum, the ion intensities were normalized to 100.%

The intensity ratios in the experimental spectrum B were modeled by taking a linear combination of those in spectra C and A, corresponding to mol fractions x_1 ($\mathbf{PtC_4D}$) and $1\text{-}x_1$ (natural abundance $\mathbf{PtC_4H}$). The best fit for the experimental 1020:1021:1022:1023:1024 ion ratio in spectrum B (relative intensities 53:91:100:52:26) was found for $x_1 = 0.82$ and $1\text{-}x_1 = 0.18$. This corresponds to 82% deuterium labeling in the sample produced from $\mathbf{PtC_4H}$, *n*-BuLi, and D_2O (spectrum B).

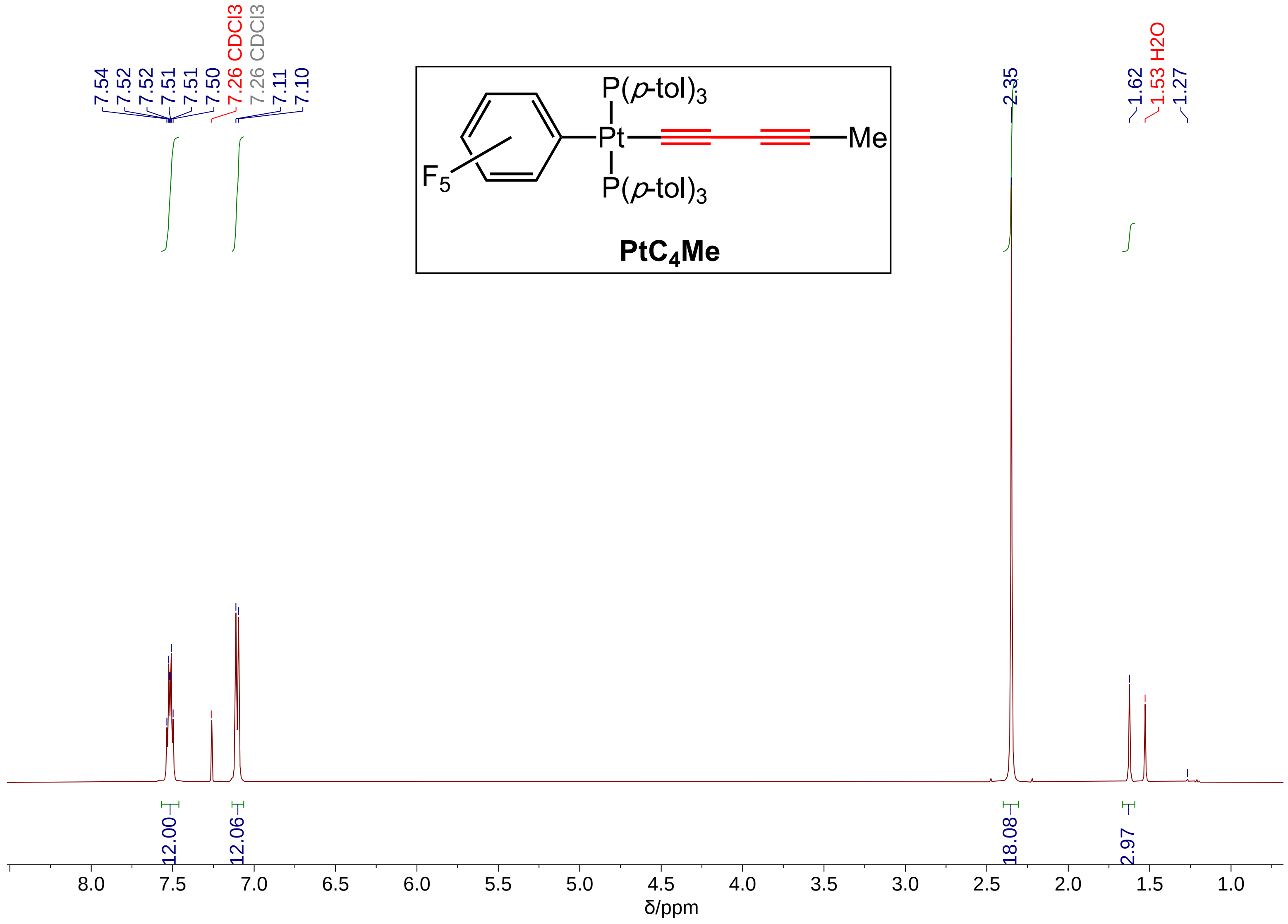

Figure s4. ^{1}H NMR spectrum of **PtC_4Me** ($CDCl_3$, 500 MHz).

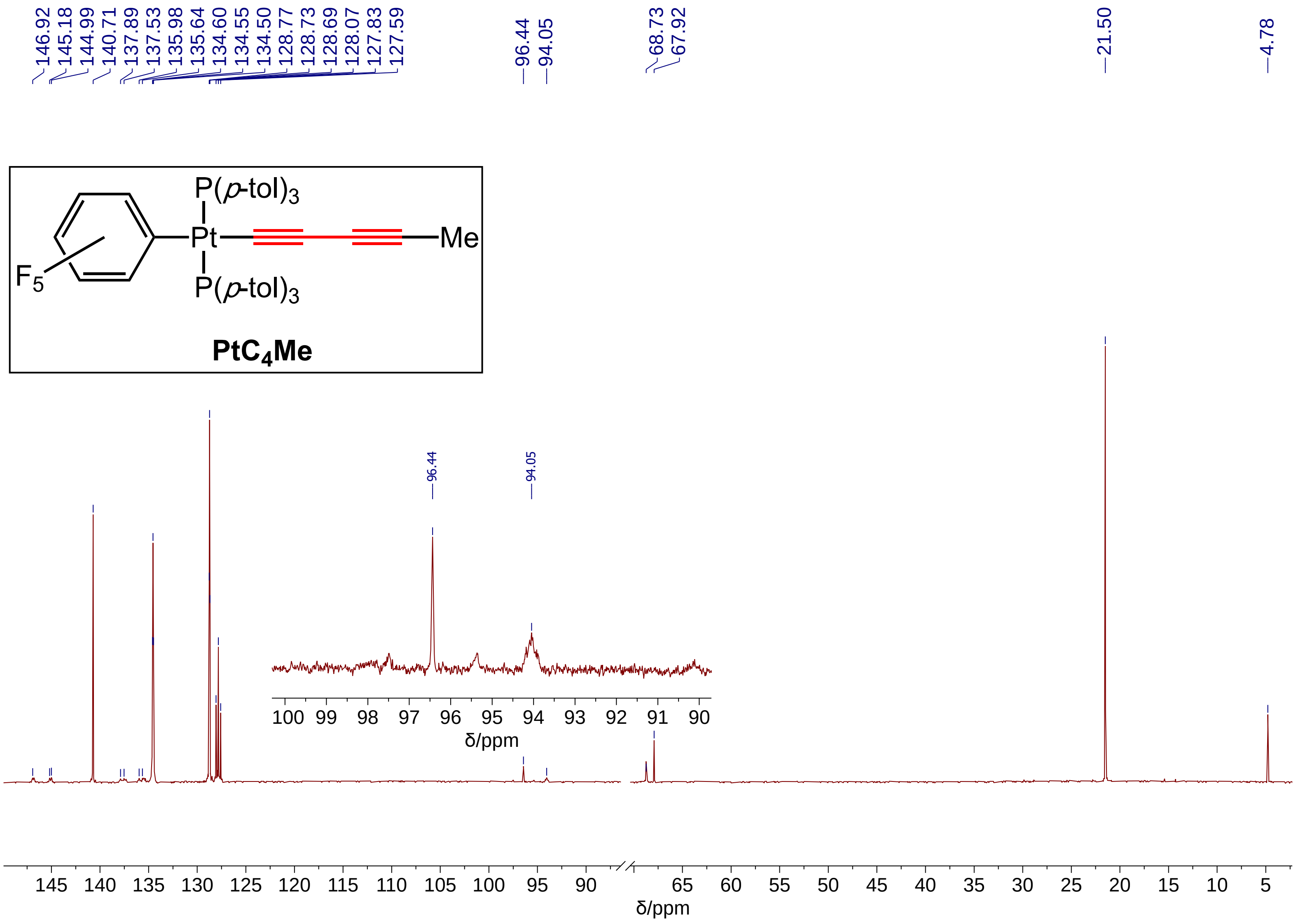

Figure s5. $^{13}C\{^{1}H\}$ NMR spectrum of **PtC_4Me** ($CDCl_3$, 126 MHz). The intense $CDCl_3$ solvent peak (77.00 ppm) has been excised.

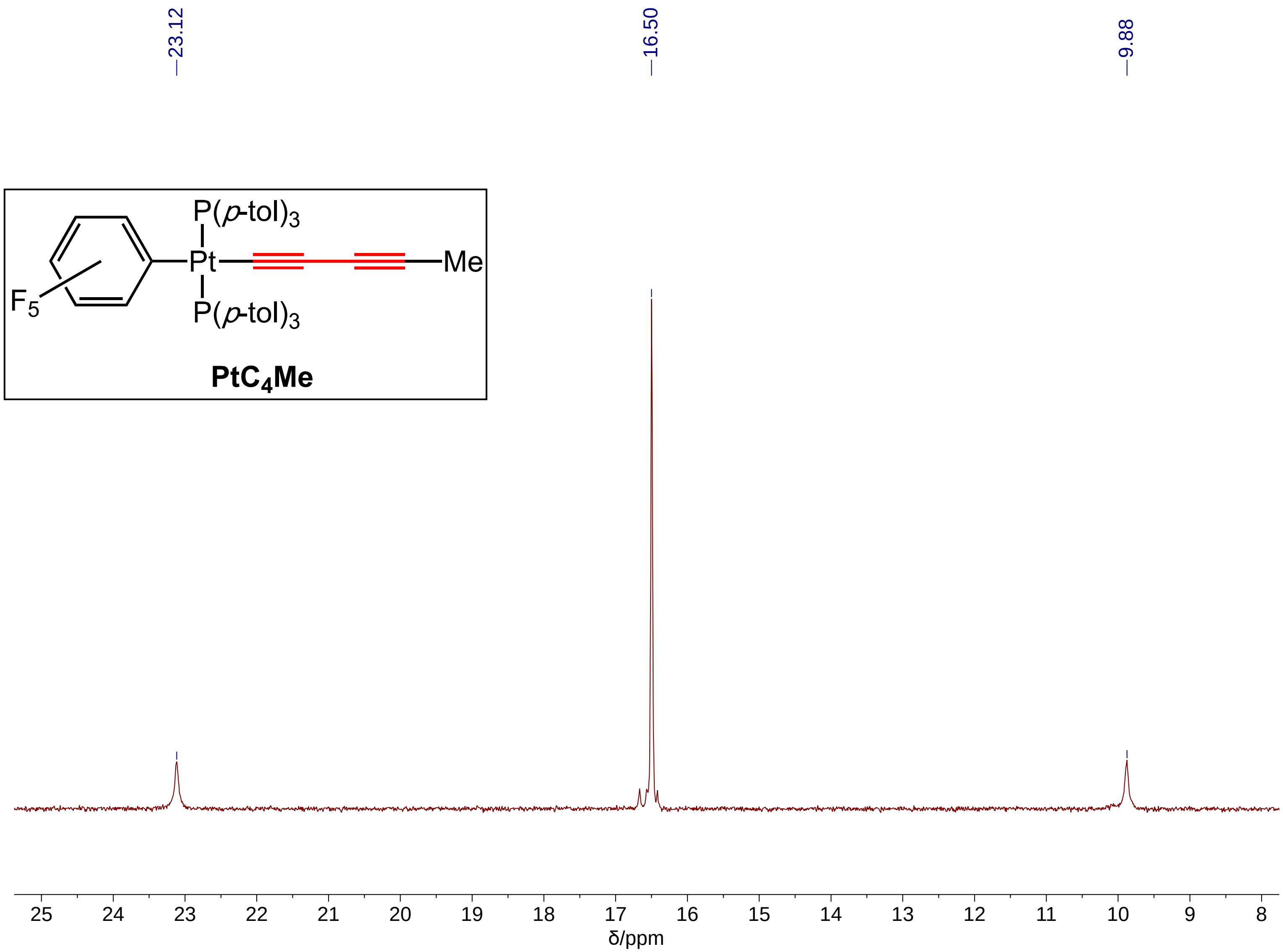

Figure s6. $^{31}P\{^{1}H\}$ NMR spectrum of **PtC$_4$Me** ($CDCl_3$, 202 MHz).

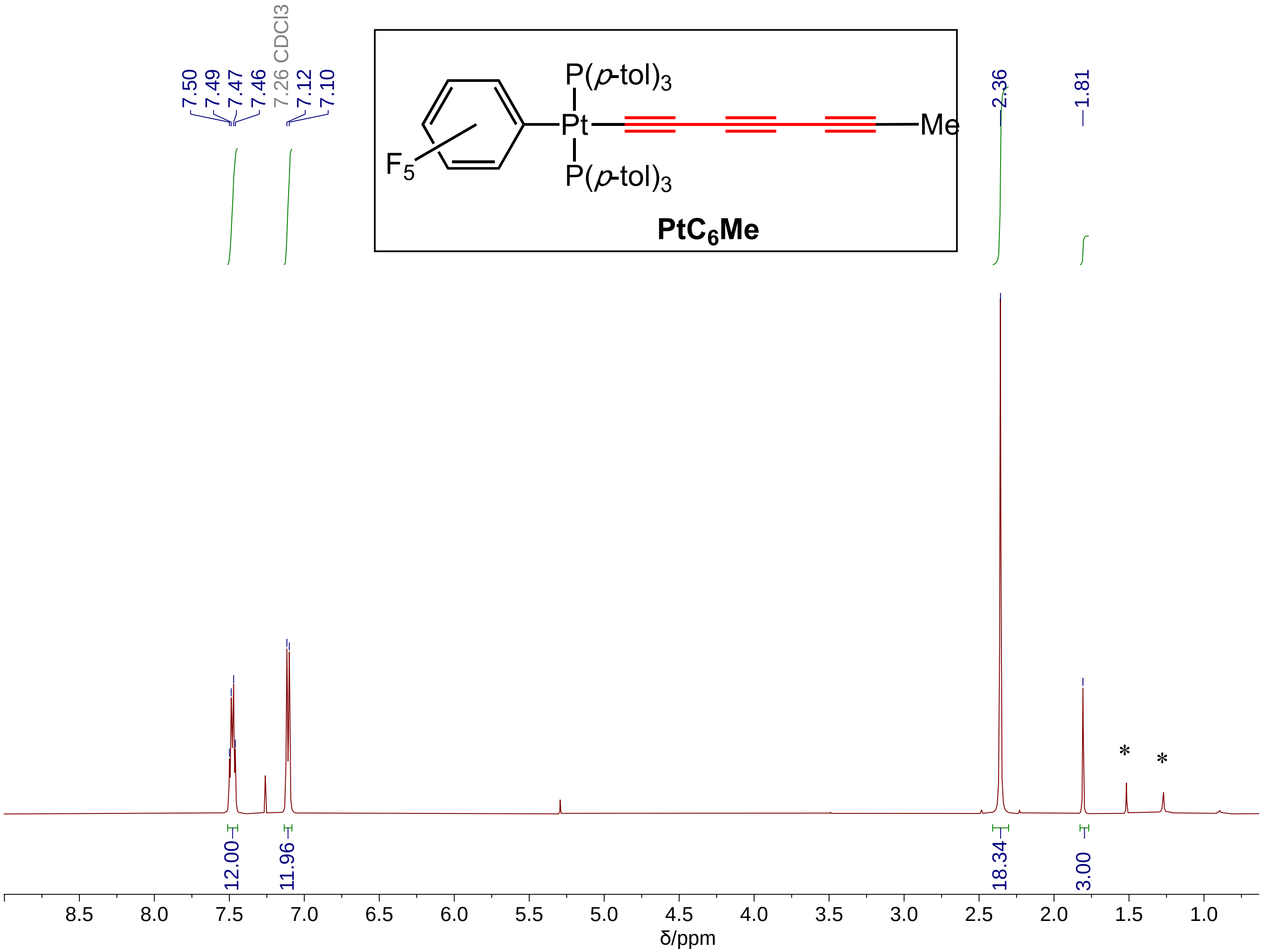

Figure s7. ^{1}H NMR spectrum of **PtC$_6$Me** ($CDCl_3$, 500 MHz). * denotes a solvent based impurity.

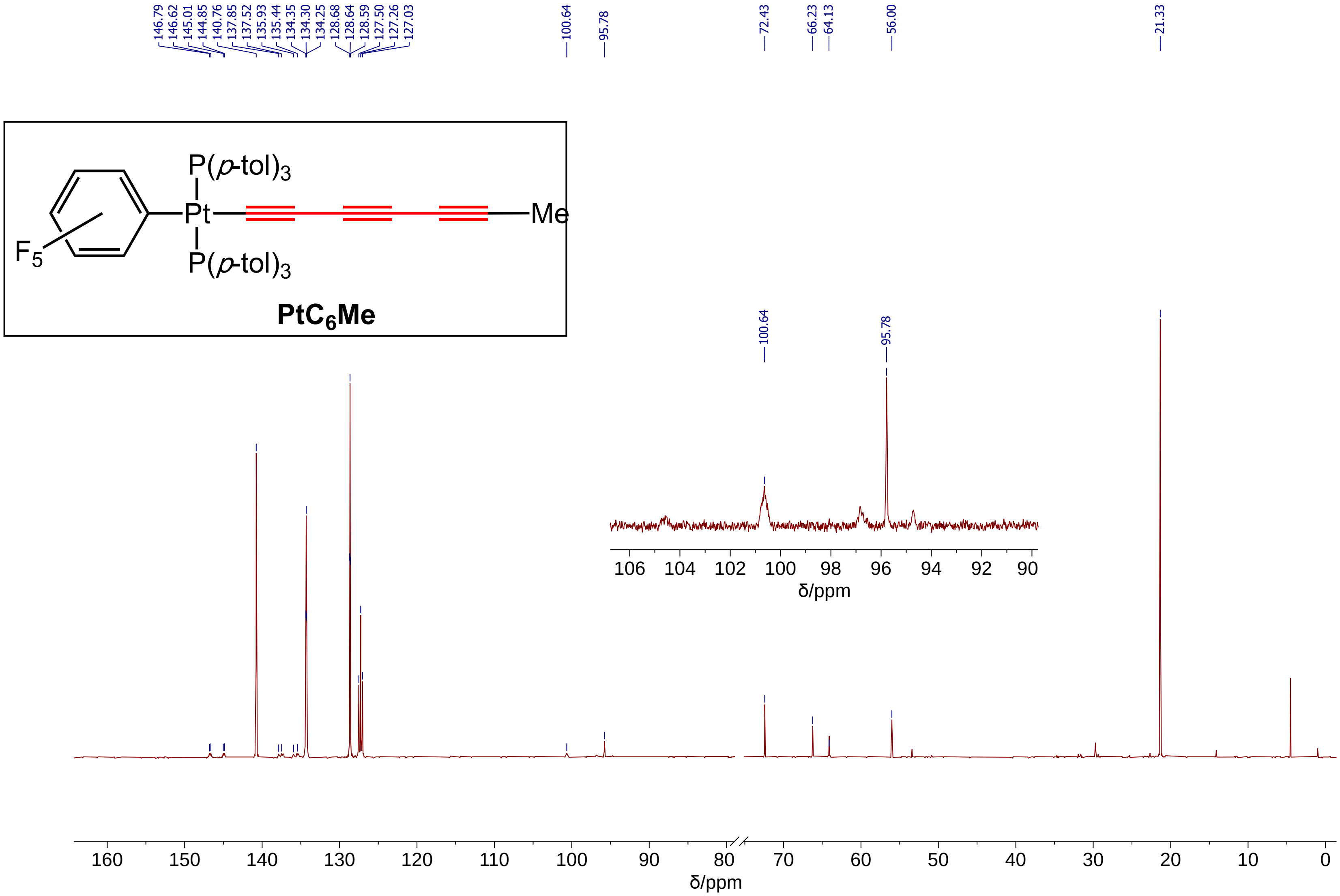

Figure s8. $^{13}C\{^1H\}$ NMR spectrum of $\mathbf{PtC_6Me}$ ($CDCl_3$, 126 MHz). The intense $CDCl_3$ solvent peak (77.00 ppm) has been excised.

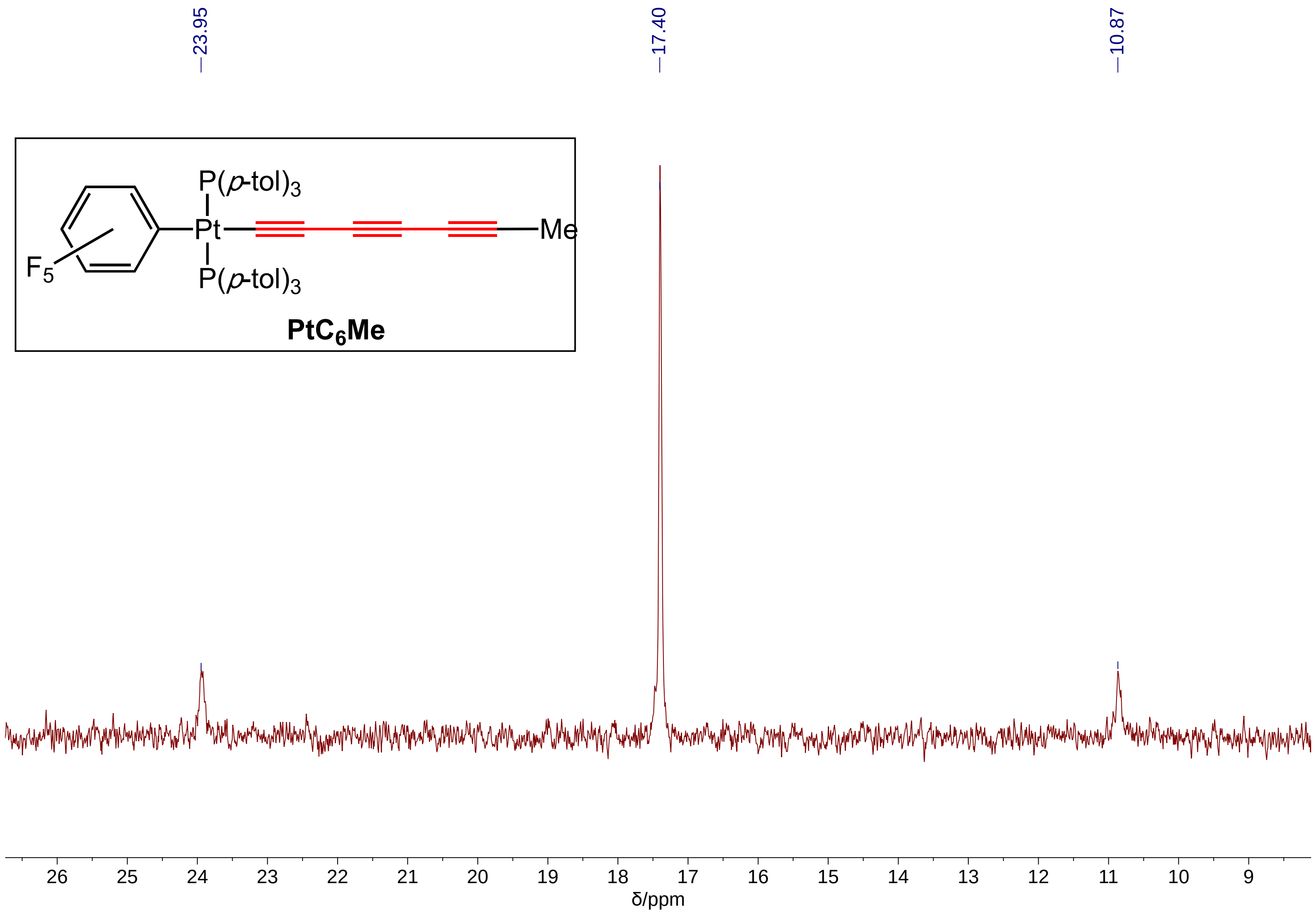

Figure s9. $^{31}P\{^{1}H\}$ NMR spectrum of **PtC_6Me** ($CDCl_3$, 202 MHz).

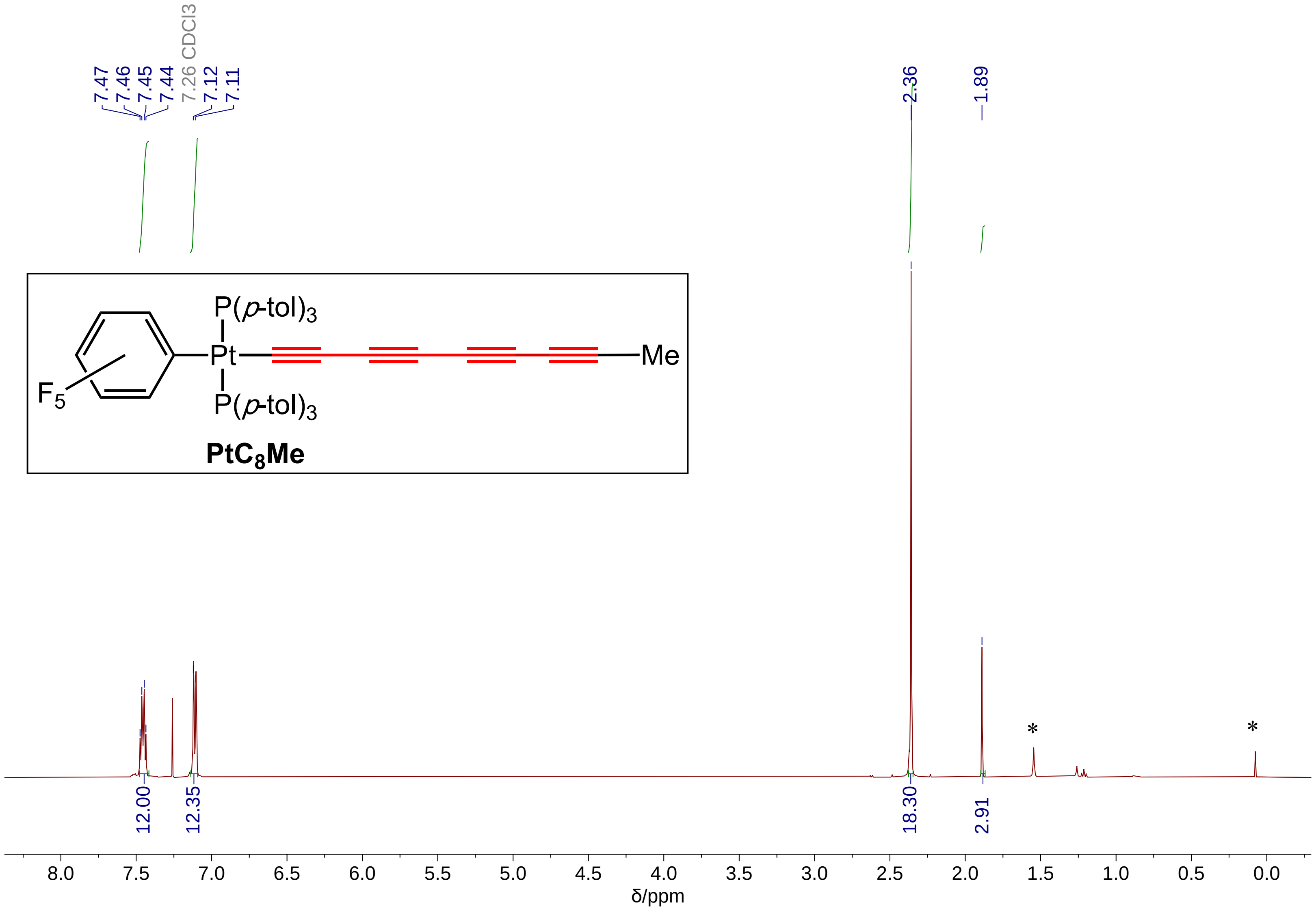

Figure s10. ^{1}H NMR spectrum of **PtC_8Me** ($CDCl_3$, 500 MHz). * denotes a solvent based impurity.

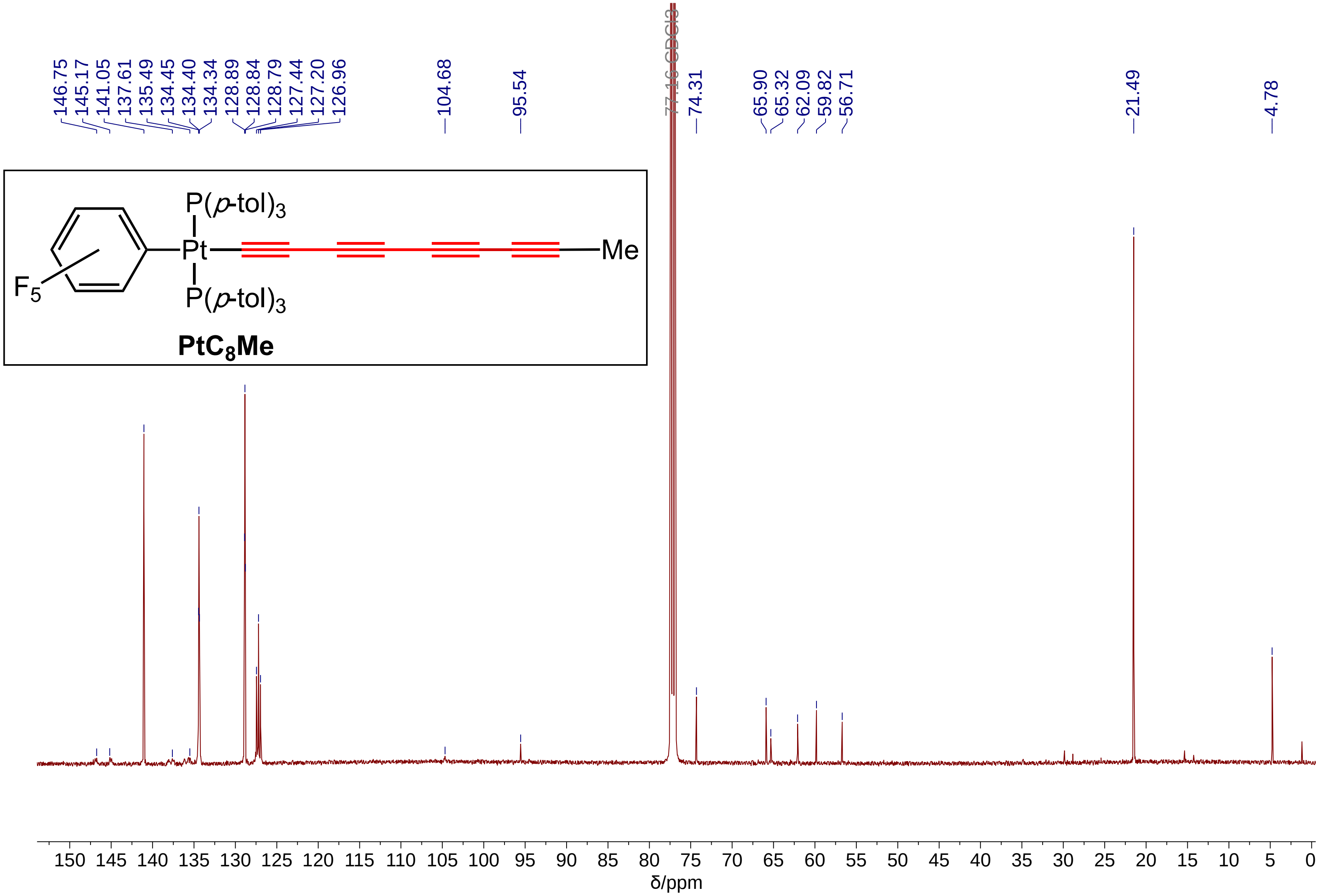

Figure s11. $^{13}C\{^1H\}$ NMR spectrum of **PtC$_8$Me** ($CDCl_3$, 126 MHz).

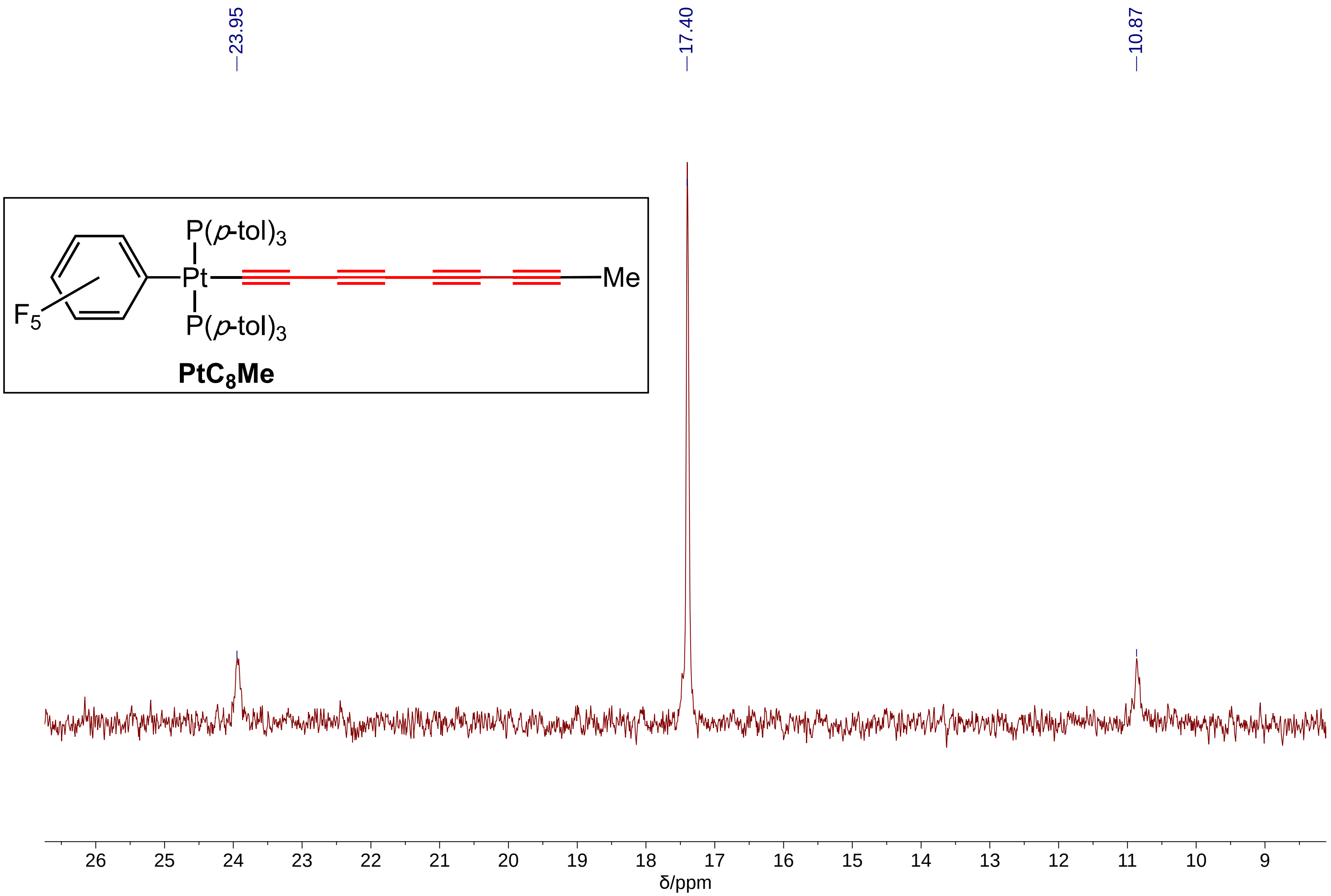

Figure s12. $^{31}P\{^1H\}$ NMR spectrum of **PtC$_8$Me** ($CDCl_3$, 202 MHz).

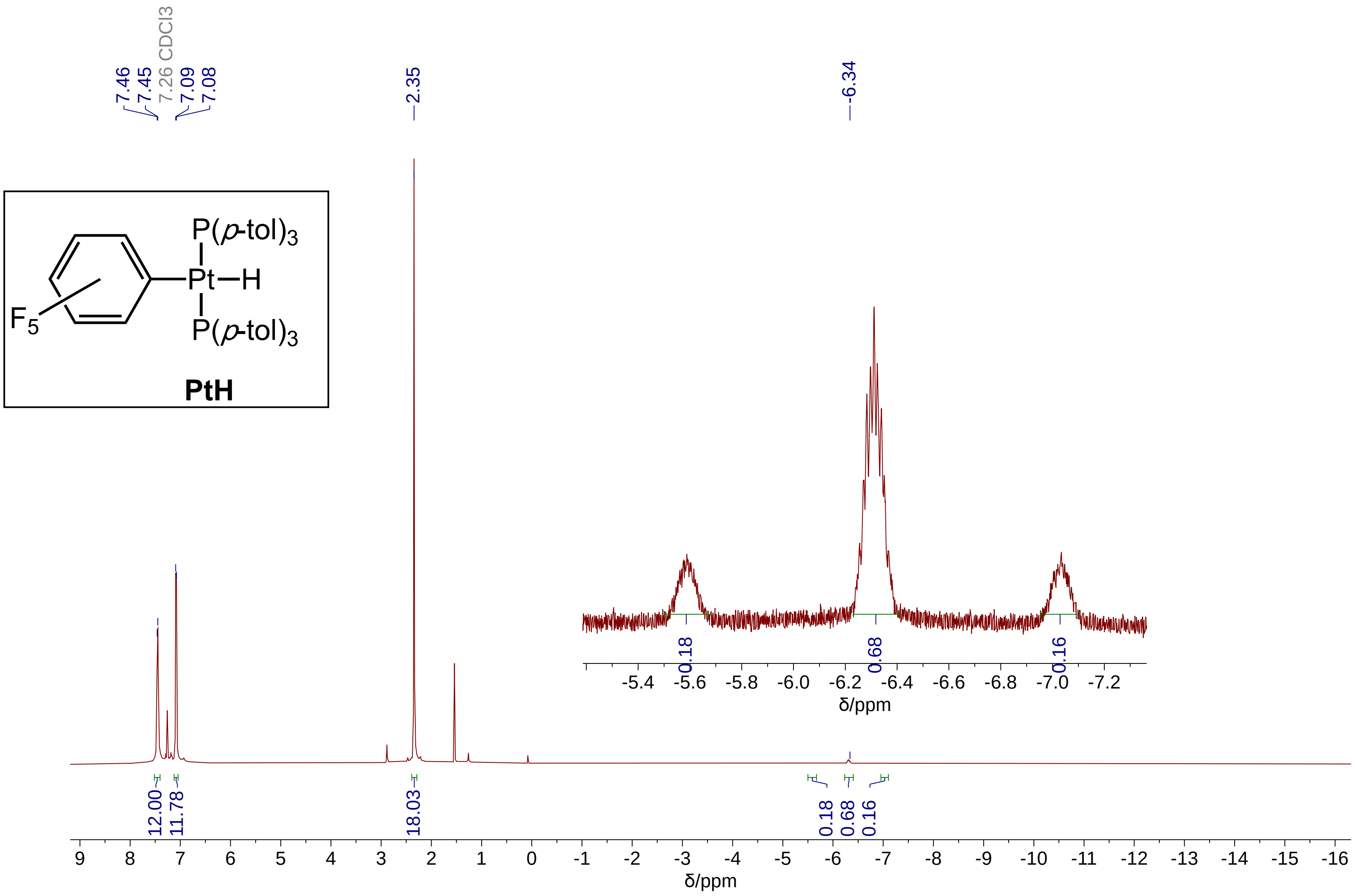

Figure s13. ^{1}H NMR spectrum of **PtH** ($CDCl_3$, 500 MHz).

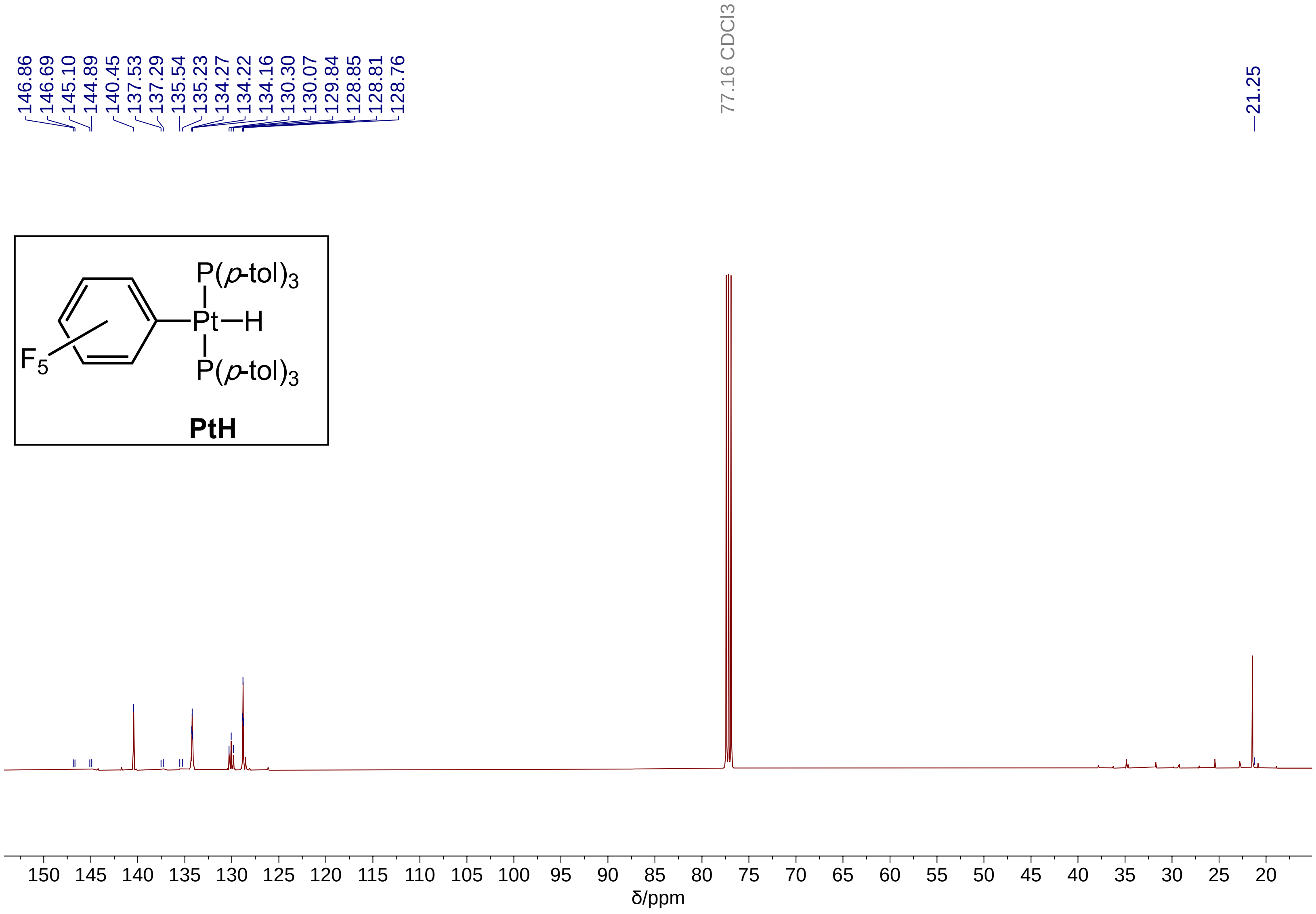

Figure s14. $^{13}C\{^{1}H\}$ NMR spectrum of **PtH** ($CDCl_3$, 126 MHz).

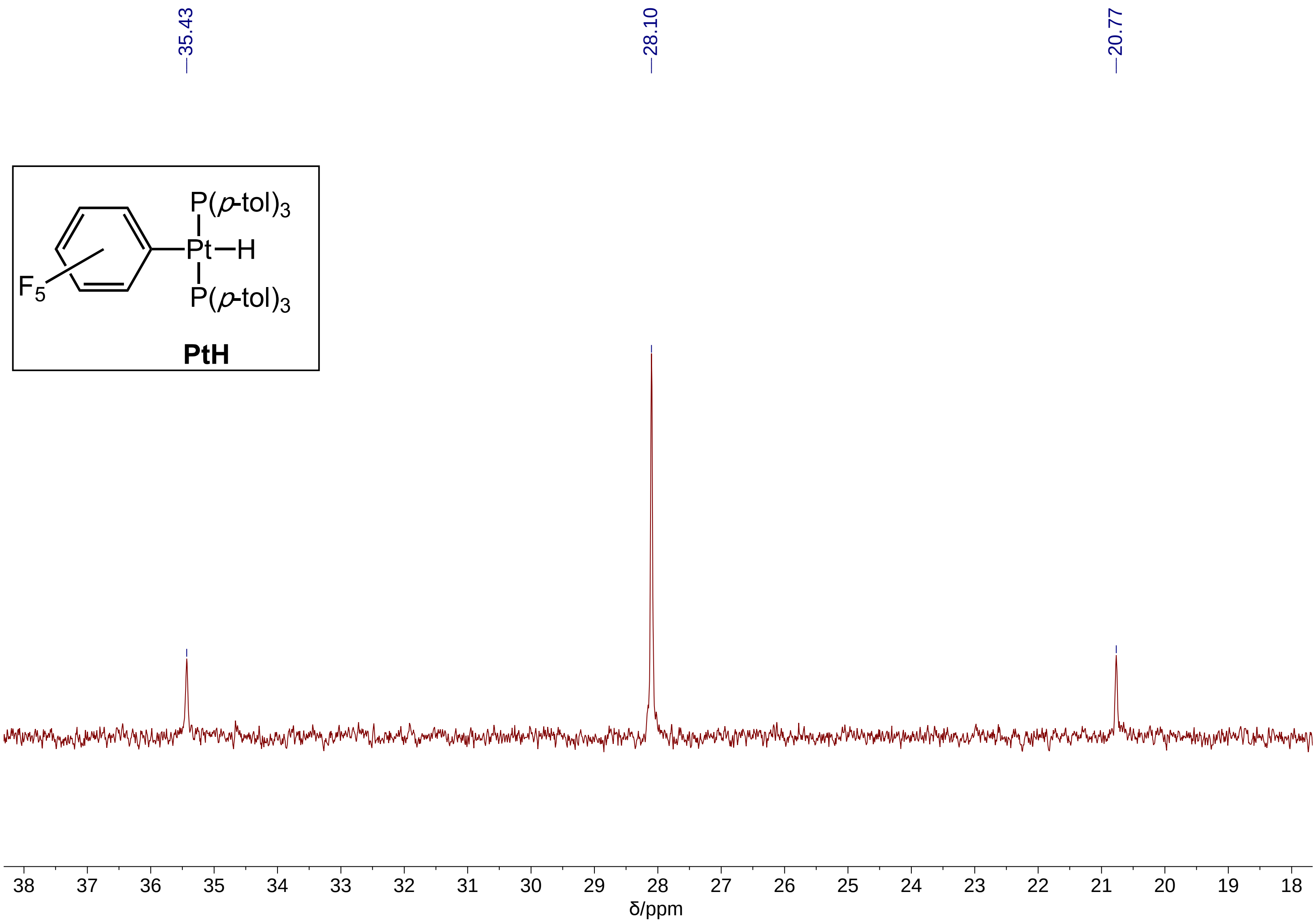

Figure s15. $^{31}P\{^{1}H\}$ NMR spectrum of **PtH** ($CDCl_3$, 202 MHz).

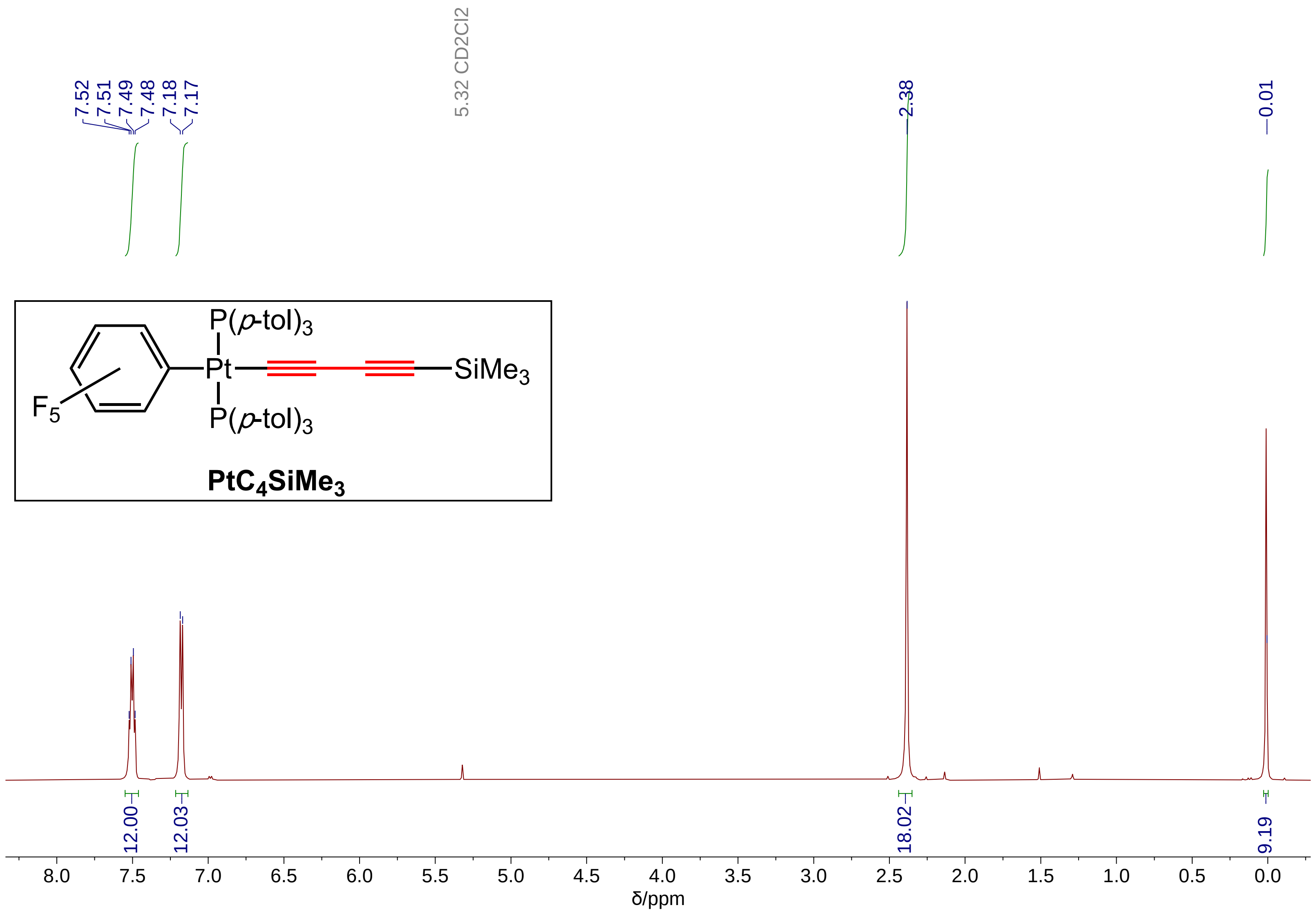

Figure s16. ^{1}H NMR spectrum of **PtC_4SiMe_3** (CD_2Cl_2, 500 MHz).

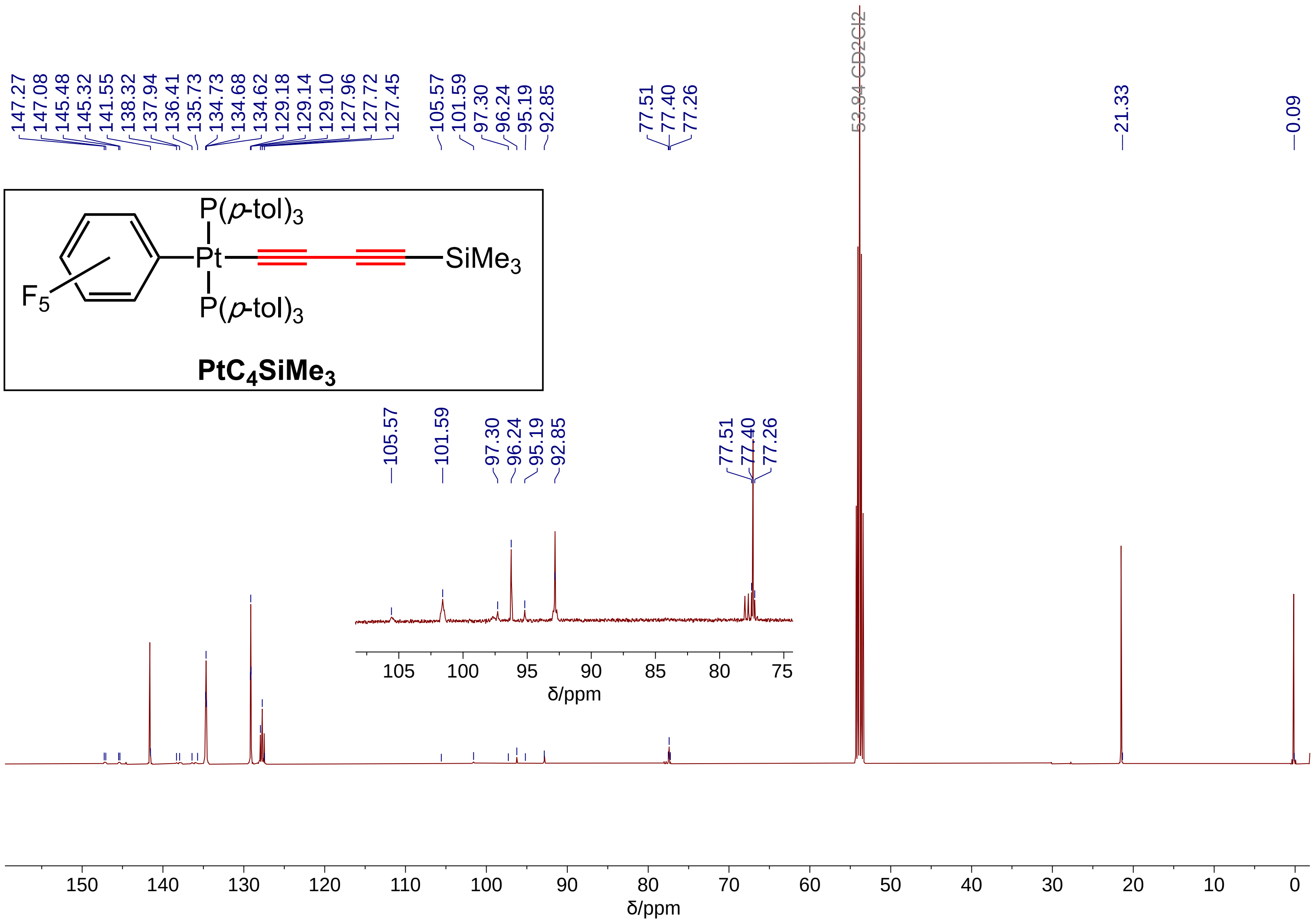

Figure s17. $^{13}C\{^1H\}$ NMR spectrum of **PtC_4SiMe_3** (CD_2Cl_2, 126 MHz).

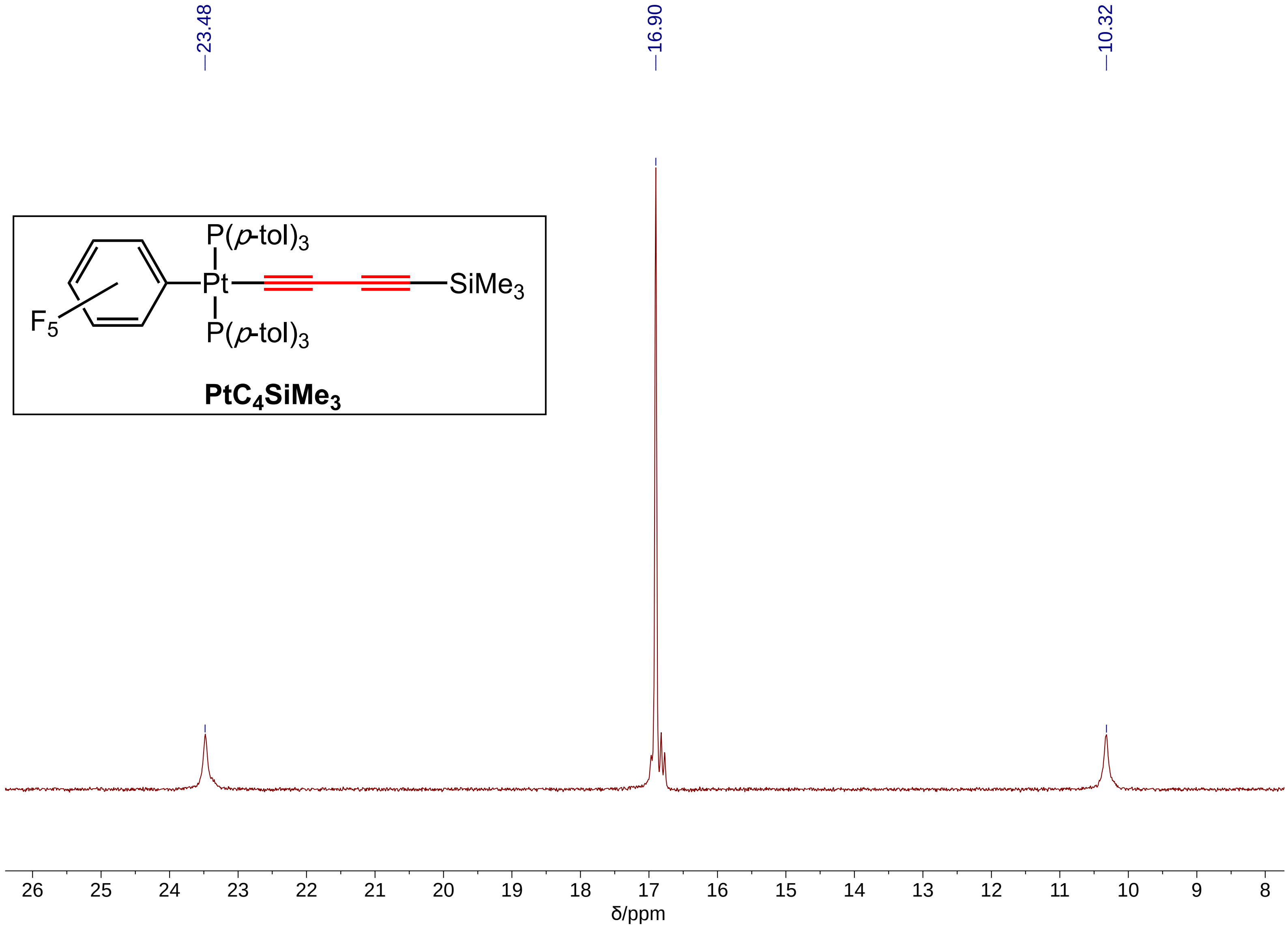

Figure s18. $^{31}P\{^1H\}$ NMR spectrum of $\mathbf{PtC_4SiMe_3}$ (CD_2Cl_2, 202 MHz).

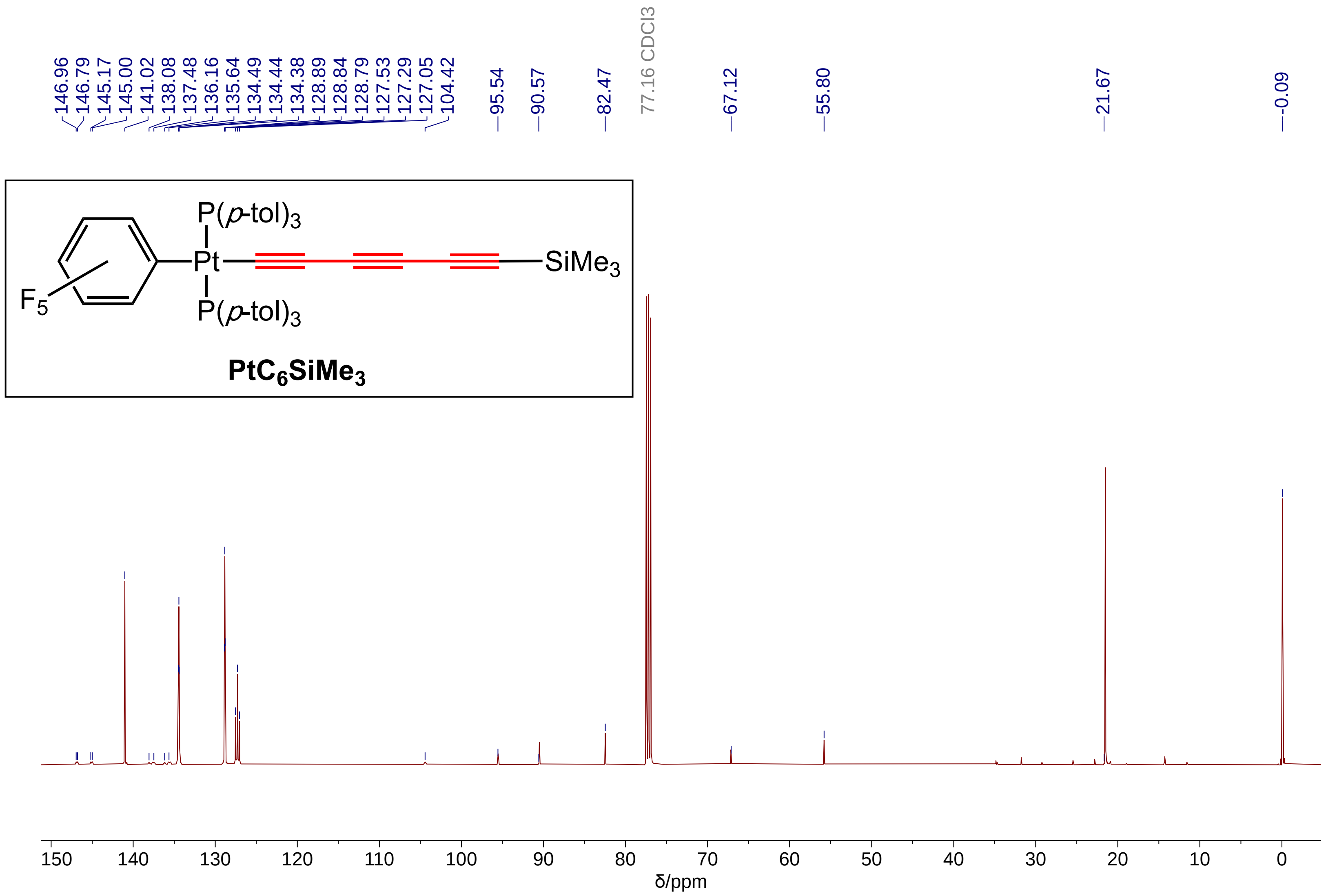

Figure s19. $^{13}C\{^{1}H\}$ NMR spectrum of **PtC_6SiMe_3** ($CDCl_3$, 126 MHz).

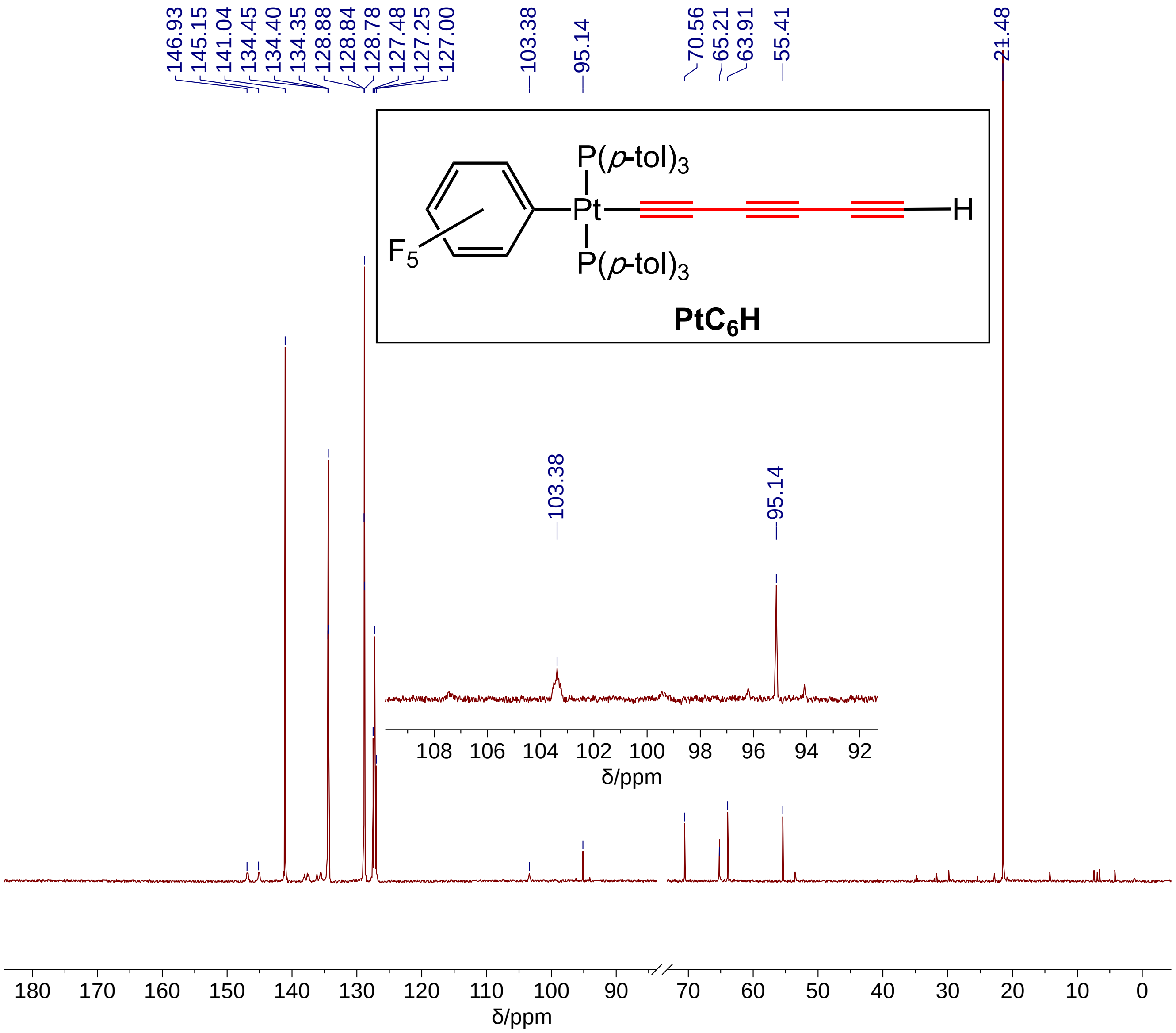

Figure s20. $^{13}C\{^1H\}$ NMR spectrum of **PtC$_6$H** ($CDCl_3$, 126 MHz). The intense $CDCl_3$ solvent peak (77.00 ppm) has been excised.

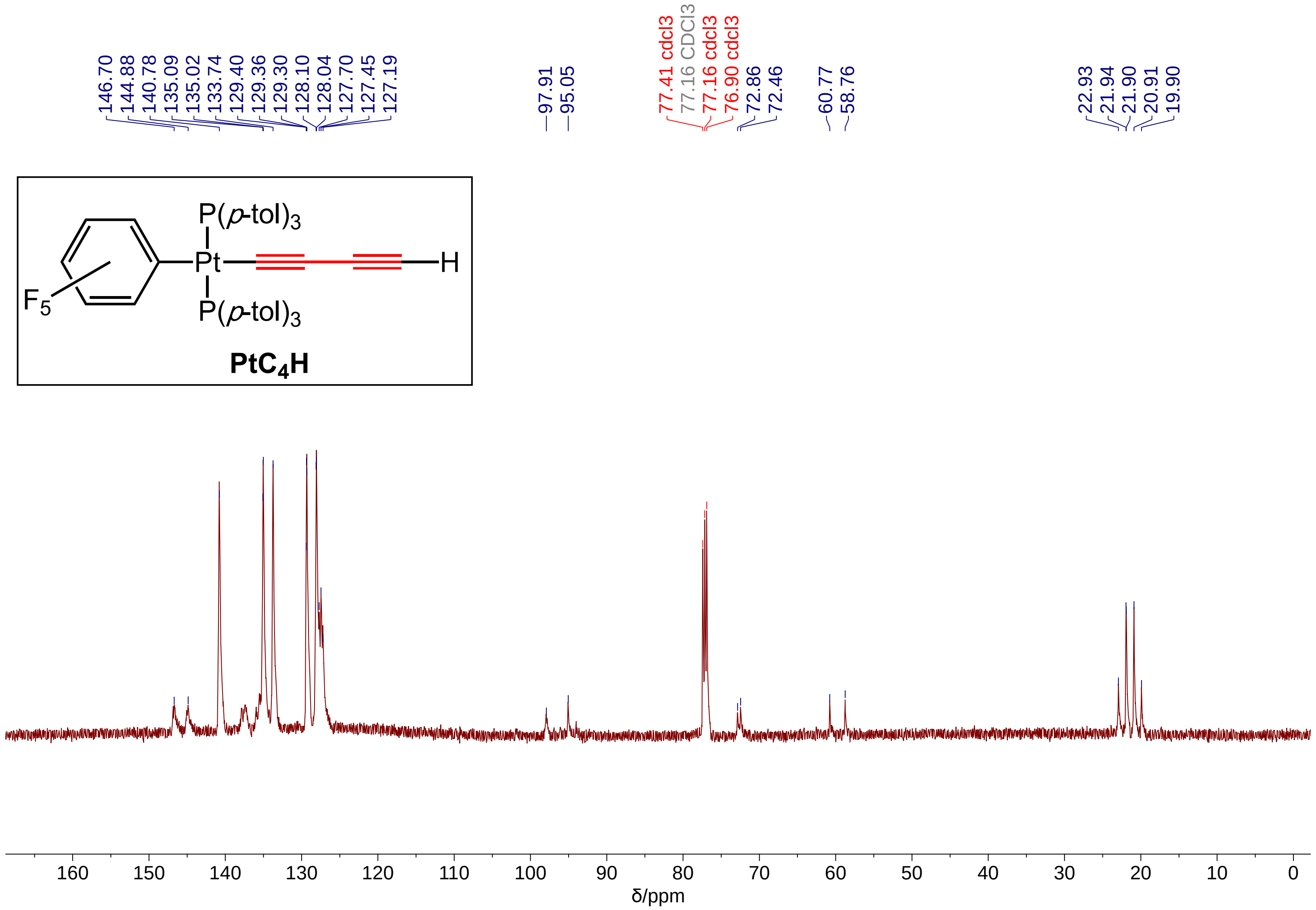

Figure s21. ^{13}C NMR spectrum of **PtC$_4$H** ($CDCl_3$, 126 MHz).

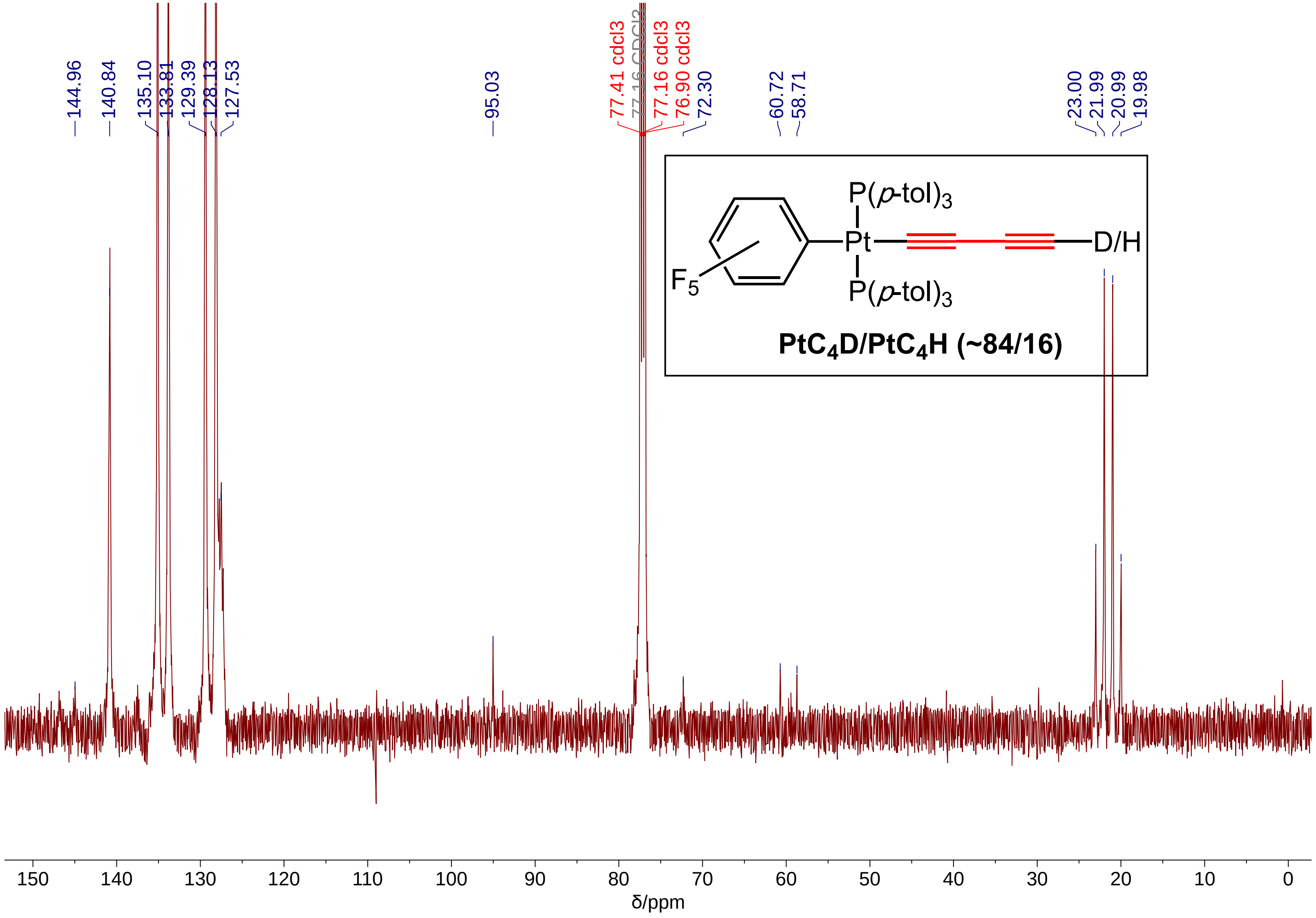

Figure s22. ^{13}C NMR spectrum of **PtC_4D** ($CDCl_3$, 126 MHz).

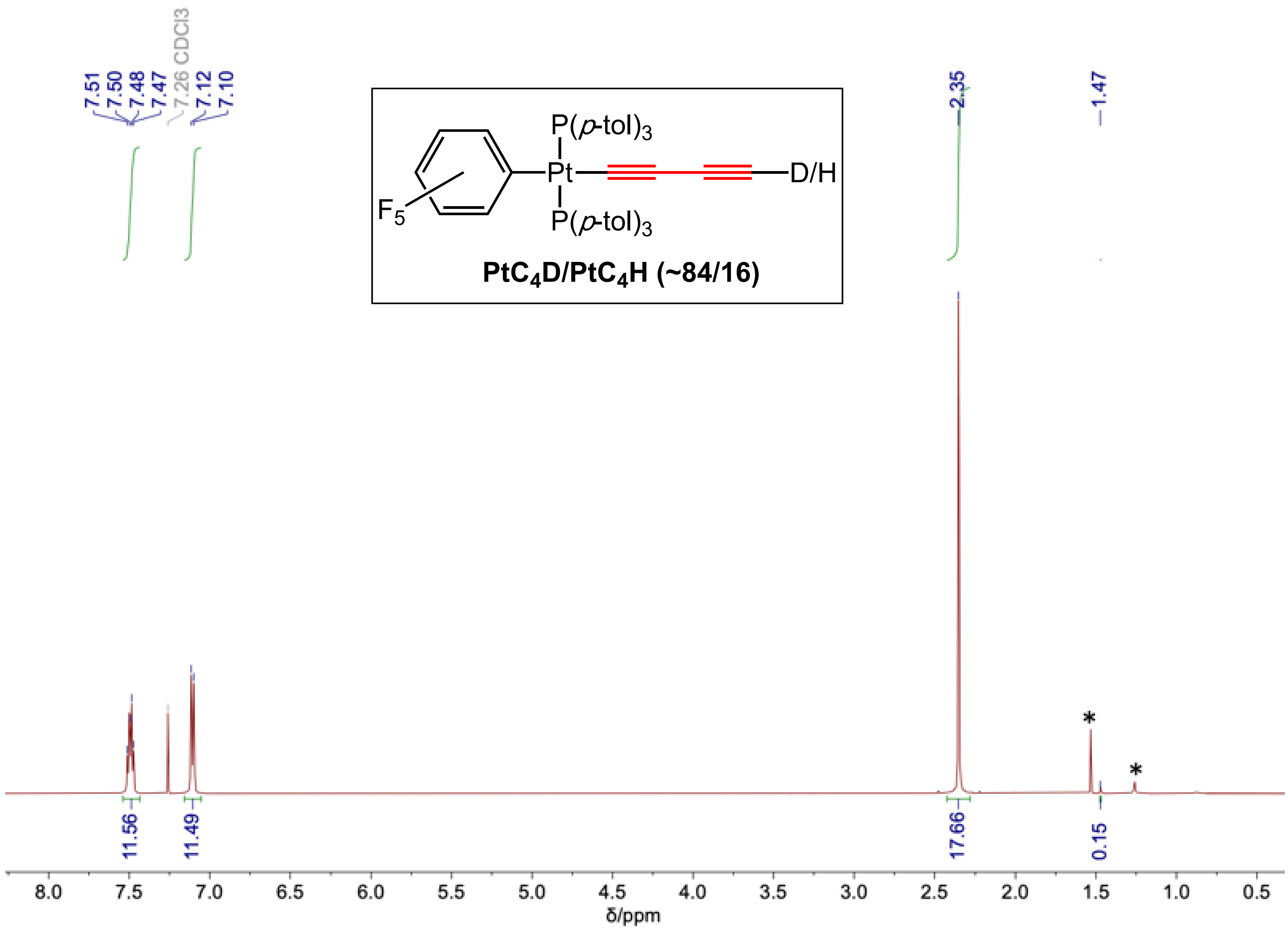

Figure s23. ^{1}H NMR spectrum of **PtC_4D** ($CDCl_3$, 500 MHz). The 1.47 ppm signal denotes residual ≡C*H* as further analyzed in Figure s3. * denotes a solvent based impurity.

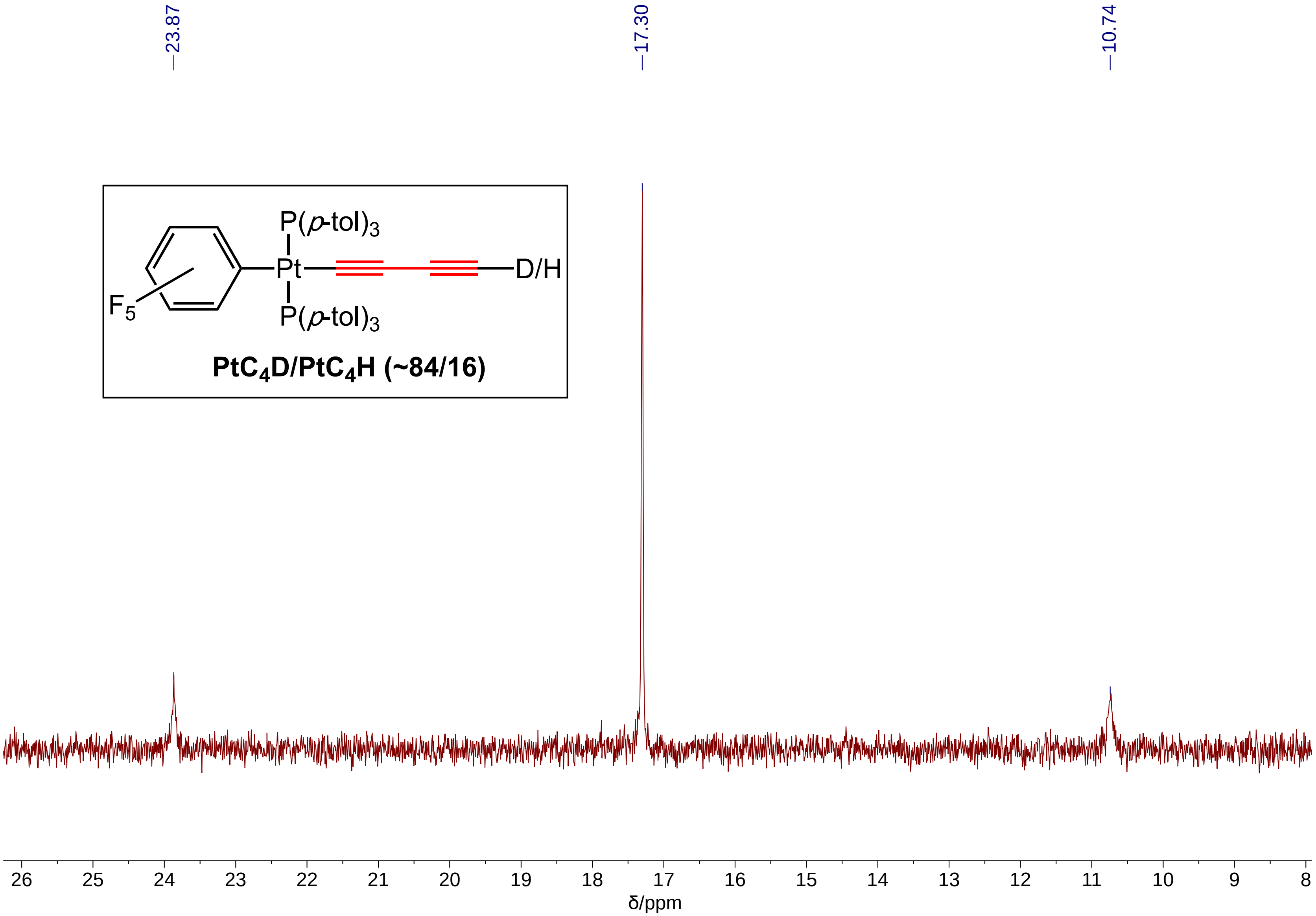

Figure s24. $^{31}P\{^{1}H\}$ NMR spectrum of **PtC_4D** ($CDCl_3$, 202 MHz).

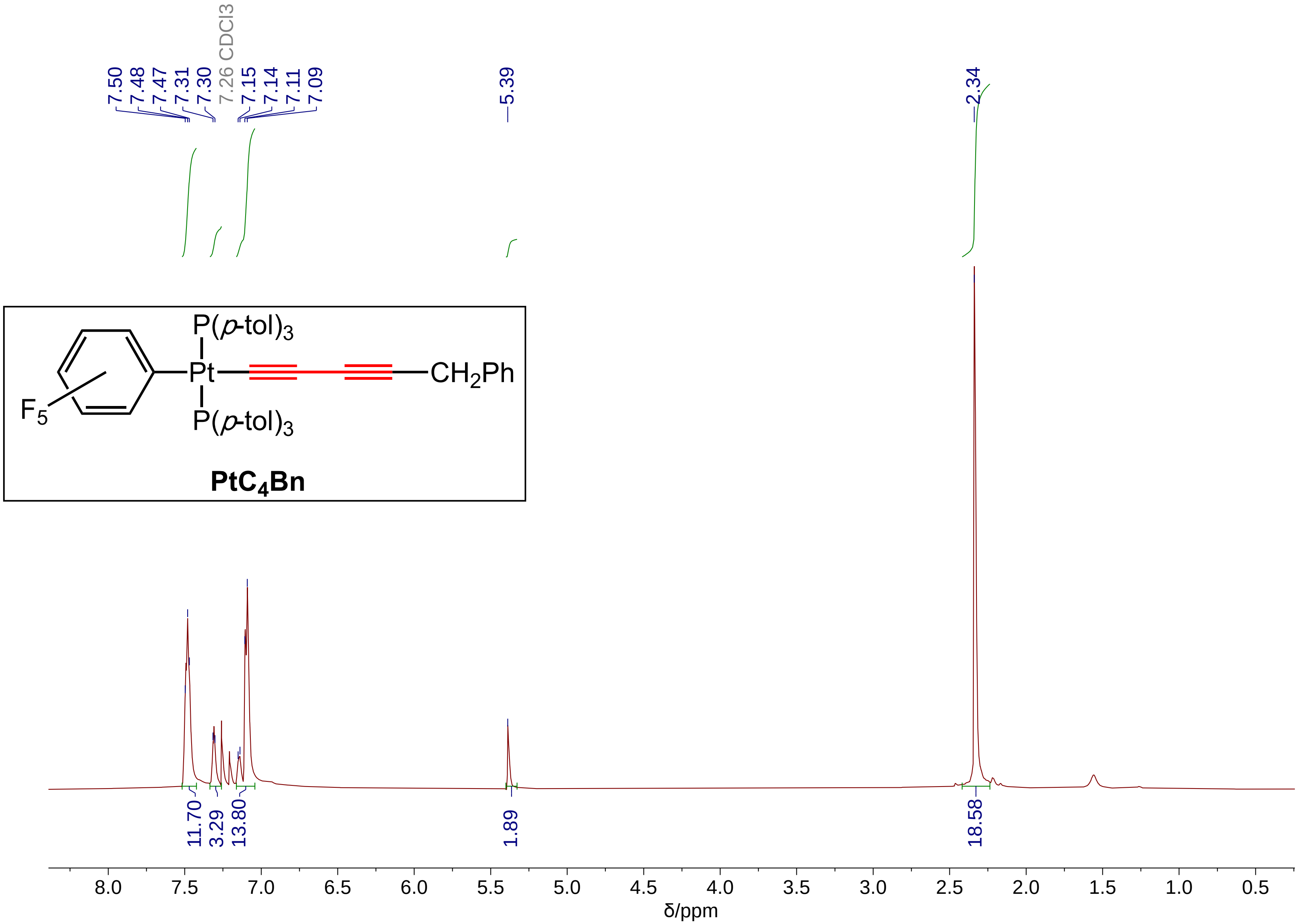

Figure s25. ^{1}H NMR spectrum of **PtC_4Bn** ($CDCl_3$, 500 MHz).

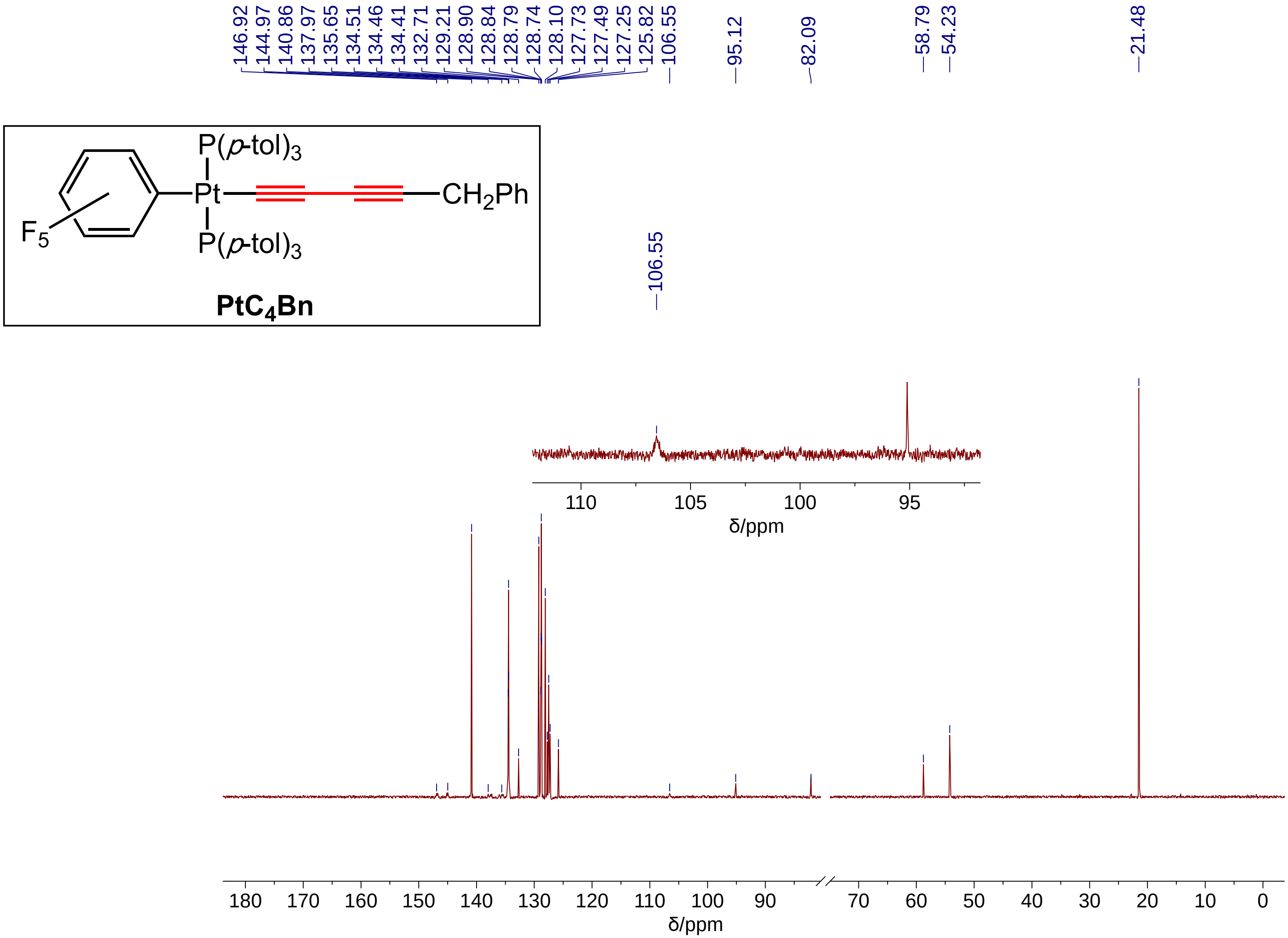

Figure s26. $^{13}C\{^{1}H\}$ NMR spectrum of **PtC$_4$Bn** ($CDCl_3$, 126 MHz). The intense $CDCl_3$ solvent peak (77.00 ppm) has been excised.

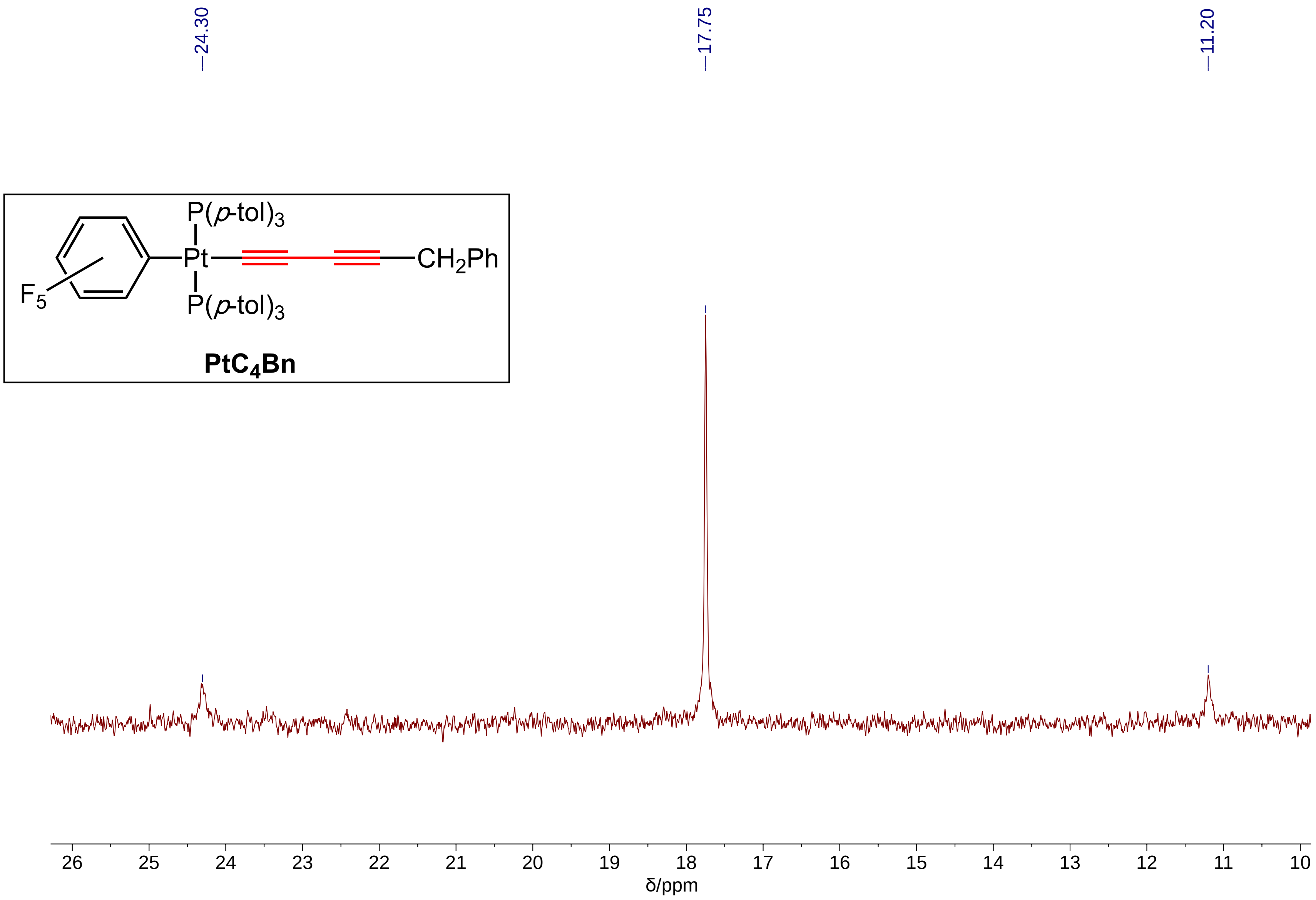

Figure s27. $^{31}P\{^1H\}$ NMR spectrum of **PtC_4Bn** ($CDCl_3$, 202 MHz).

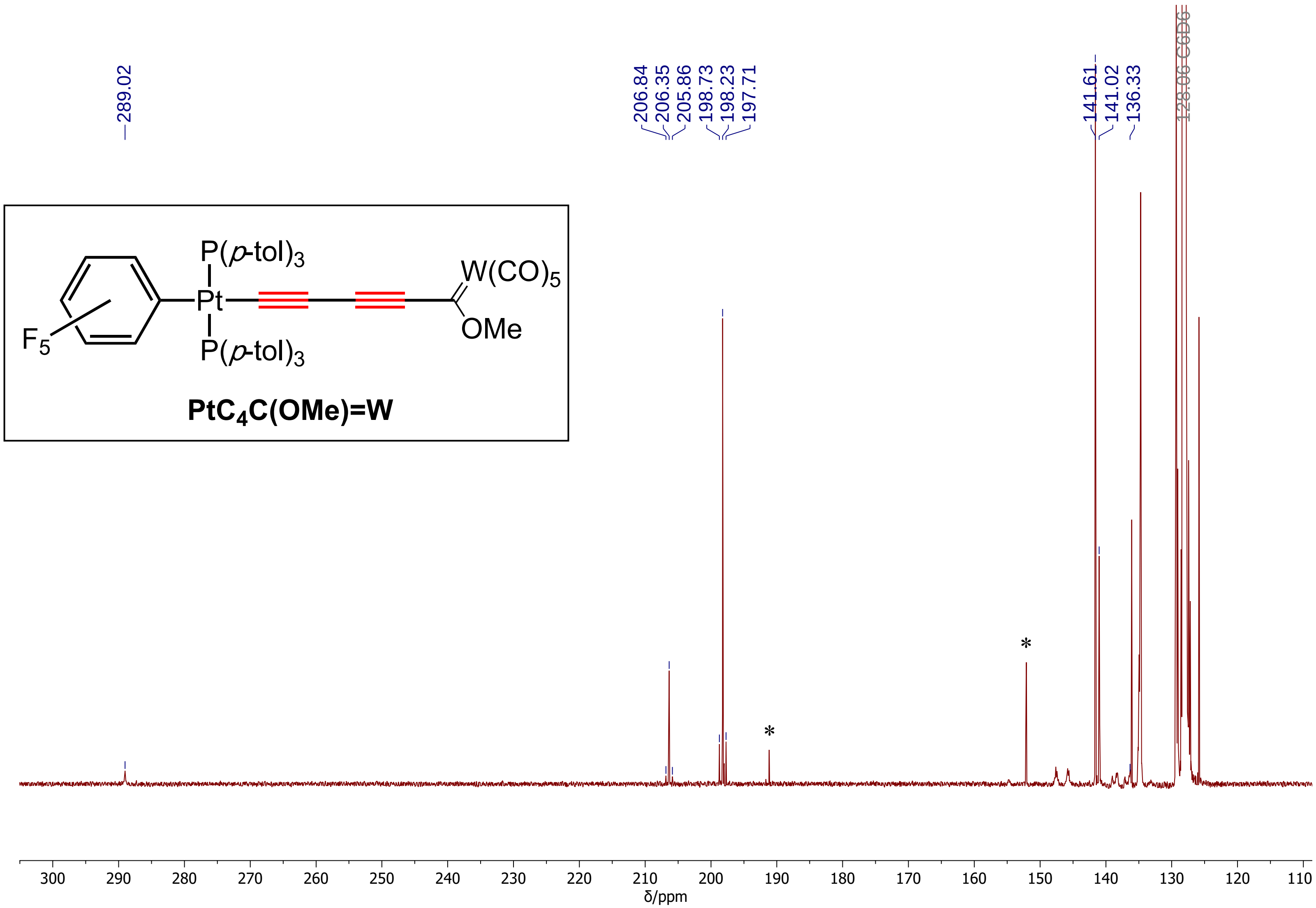

Figure s28. Downfield region of the $^{13}C\{^{1}H\}$ NMR spectrum of **PtC$_4$C(OMe)=W** (C_6D_6, 126 MHz). * denotes impurity peak.

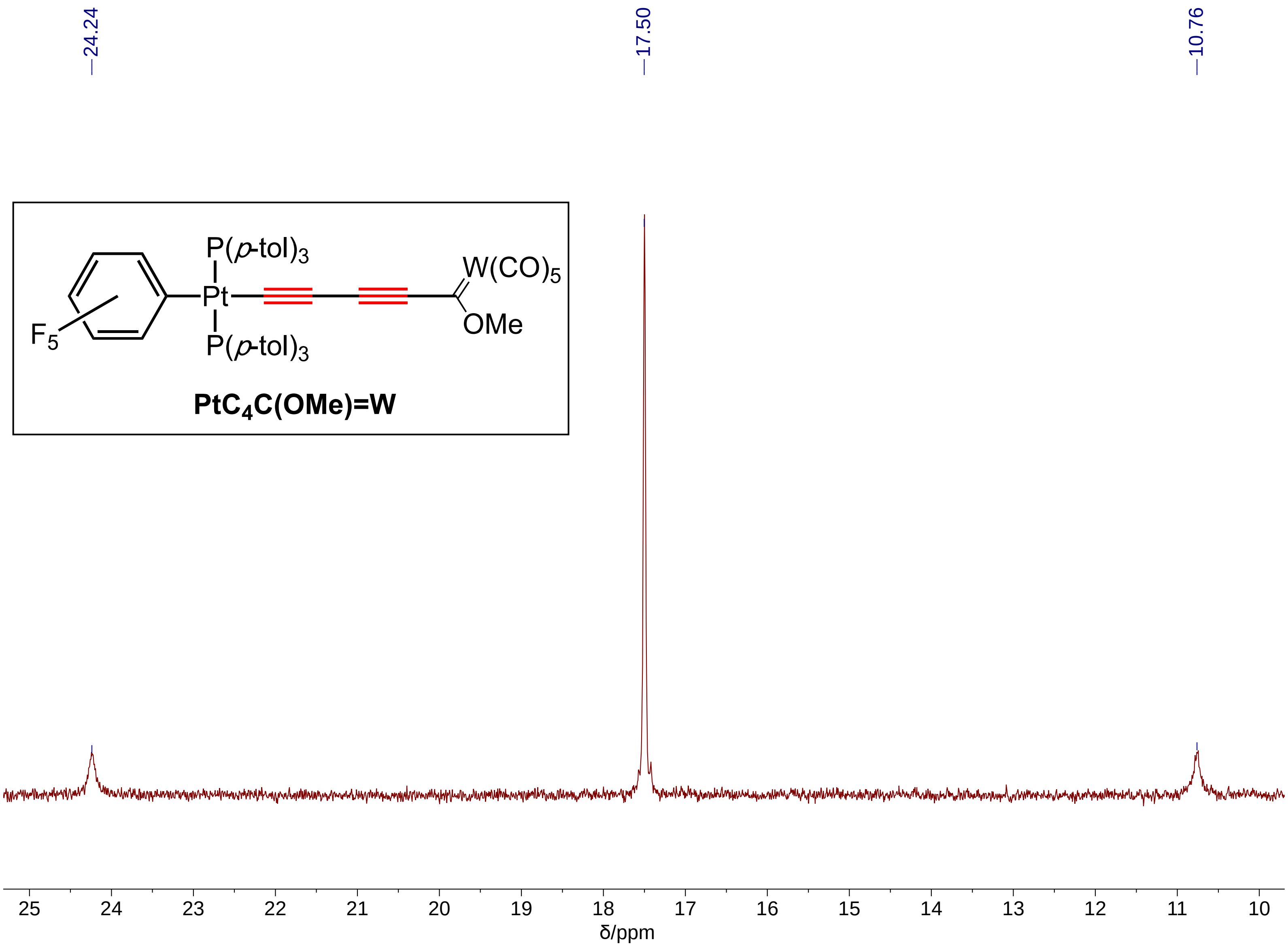

Figure s29. $^{31}P\{^1H\}$ NMR spectrum of **$PtC_4C(OMe)$=W** (C_6D_6, 202 MHz).
